# Theoretical foundations and implications of augmented reality, virtual reality, and mixed reality for immersive learning in health professions education

**DOI:** 10.1186/s41077-024-00311-5

**Published:** 2024-09-09

**Authors:** Maryam Asoodar, Fatemeh Janesarvatan, Hao Yu, Nynke de Jong

**Affiliations:** 1 School of Health Professions Education, Department of Educational Development and Research, Faculty of Health, Medicine and Life sciences, Maastricht University, Universiteitssingel 60, Maastricht, 6229 MD The Netherlands; 2 Department of Health Services Research, Faculty of Health, Medicine and Life Sciences, Maastricht University, Maastricht, The Netherlands; 3 School of Business and Economics, Educational Research and Development Maastricht University, Maastricht, The Netherlands

**Keywords:** Immersive learning, AR VR MR, Instructional design models or theories, Health professions education

## Abstract

**Background:**

Augmented Reality (AR), Virtual Reality (VR) and Mixed Reality (MR) are emerging technologies that can create immersive learning environments for health professions education. However, there is a lack of systematic reviews on how these technologies are used, what benefits they offer, and what instructional design models or theories guide their use.

**Aim:**

This scoping review aims to provide a global overview of the usage and potential benefits of AR/VR/MR tools for education and training of students and professionals in the healthcare domain, and to investigate whether any instructional design models or theories have been applied when using these tools.

**Methodology:**

A systematic search was conducted in several electronic databases to identify peer-reviewed studies published between and including 2015 and 2020 that reported on the use of AR/VR/MR in health professions education. The selected studies were coded and analyzed according to various criteria, such as domains of healthcare, types of participants, types of study design and methodologies, rationales behind the use of AR/VR/MR, types of learning and behavioral outcomes, and findings of the studies. The (Morrison et al. John Wiley & Sons, 2010) model was used as a reference to map the instructional design aspects of the studies.

**Results:**

A total of 184 studies were included in the review. The majority of studies focused on the use of VR, followed by AR and MR. The predominant domains of healthcare using these technologies were surgery and anatomy, and the most common types of participants were medical and nursing students. The most frequent types of study design and methodologies were usability studies and randomized controlled trials. The most typical rationales behind the use of AR/VR/MR were to overcome limitations of traditional methods, to provide immersive and realistic training, and to improve students’ motivations and engagements. The most standard types of learning and behavioral outcomes were cognitive and psychomotor skills. The majority of studies reported positive or partially positive effects of AR/VR/MR on learning outcomes. Only a few studies explicitly mentioned the use of instructional design models or theories to guide the design and implementation of AR/VR/MR interventions.

**Discussion and conclusion:**

The review revealed that AR/VR/MR are promising tools for enhancing health professions education, especially for training surgical and anatomical skills. However, there is a need for more rigorous and theory-based research to investigate the optimal design and integration of these technologies in the curriculum, and to explore their impact on other domains of healthcare and other types of learning outcomes, such as affective and collaborative skills. The review also suggested that the (Morrison et al. John Wiley & Sons, 2010) model can be a useful framework to inform the instructional design of AR/VR/MR interventions, as it covers various elements and factors that need to be considered in the design process.

**Supplementary Information:**

The online version contains supplementary material available at 10.1186/s41077-024-00311-5.

## Introduction

Health professions education is a dynamic and complex field that requires constant adaptation to the changing needs of society and the health care system [20, 71]. One of the emerging trends in this field is the use of virtual technologies, such as augmented reality (AR), virtual reality (VR), and mixed reality (MR), to enhance the teaching and learning of various skills and competencies. These technologies offer the potential to create immersive, interactive, and realistic environments that can facilitate learning through feedback, reflection, and practice, while reducing the risks and costs associated with real-life scenarios. However, the effective integration of these technologies into health professions education depends on the sound application of instructional design principles and theories, as well as the evaluation of learning outcomes and impacts. This scoping review aims to provide a comprehensive overview of the current state of the art of using AR/VR/MR in health professions education, with a focus on the instructional design aspects and the learning and behavioral outcomes reported in the literature.

Current educational methods in health professions training encompass various approaches. These include problem-based learning [70], team-based learning [1], eLearning (Van Nuland et al. [19]), and simulation-based medical education (SBME) [19]. Recently, virtual technologies have emerged in alignment with educational trends. Augmented Reality (AR, Virtual Reality (VR, and Mixed Reality (MR are increasingly utilized not only in general education but also specifically in health professions education (Van Nuland et al. [19],). These technologies offer a range of potential strategies for comprehensive and practical training, contributing to safer patient care [19].

In the field of healthcare, diverse AR/VR/MR applications are already in use to train healthcare professionals, primarily assisting in surgical procedures for enhanced navigation and visualization [9, 62]. These applications aim to facilitate learning through immersion, reflection, feedback, and practice, all while mitigating the inherent risks of real-life experiences. Simulators play a pivotal role in introducing novel teaching methods for complex medical content [16, 21, 27, 29, 35]. They allow repeated practice across a wide spectrum of medical disciplines [39, 59], Peterson et al. [61] and may address challenges encountered in traditional health training programs.

VR creates an artificial environment where users interact with computer-generated sights and sounds. It immerses them in a simulated world using devices like headsets and motion sensors [69]. AR is an interactive overlay onto a real environment, where it offers an extra layer on top of the environment and the user experiences an immersive, interactive setting [13, 27]. In MR, elements of VR and AR are combined, and computer graphics interact with elements of the real world, allowing users to interact with both virtual and physical elements simultaneous [29]. Extended Reality (XR) serves as an umbrella term that unifies Augmented Reality (AR), Virtual Reality (VR), and Mixed Reality (MR) into a single category, reducing public confusion [6].

In short, AR/VR/MR technologies create digital environments that closely resemble real-world features. These environments enable trainees to learn tasks safely, whether within the bounds of realism or in entirely new experiences beyond traditional constraints [41]. Notably, in healthcare, the use of computer-enhanced learning has led to positive outcomes such as improved patient safety, enhanced training experiences, and cost reduction [34].

Investigating prior research in the field of AR/VR/MR in healthcare is important, as this reveals the current state of the field and offers guidance to researchers who are seeking suitable topics to explore and educationists who want to improve the teaching and learning at their institutes [34]. Currently, there is a lack of insight on the effective application of AR/VR/MR particularly in health professions education and their added value based on instructional design models or theories as most reviews have focused on the technological aspects on AR/VR/MR for medical education, or on comparison with other methods.

This review takes a global perspective to identify the usage and potential benefits of including AR/VR/MR tools for education and training of students and professionals in the health domain. Technologies are constantly evolving and there is a need for obtaining an overview of current trends in an educational context. No review, however, was found that had considered to study whether and how instructional design theories or models guided the use of AR/VR/MR for teaching in health professions education to optimize complex learning within a recent time frame. An important aspect in this regard is the theoretical grounding on which the use of methods, technological or otherwise, is based. Already four decades ago, Reigeluth [65] argued for the grounding of instructional design in sound theoretical models, stating that instruction is often ineffective and knowledge about instructional design needs to be taken into account in order to remedy this problem. In other words, in addition to focusing on what is taught, *how* it is taught is also of critical importance [65]. Unfortunately, interventions are often insufficiently or inconsistently grounded in such theoretical models [38], Reigeluth & Carr-Chellman [66].

By now, numerous instructional design models exist that can serve as the basis for determining how content should be taught [32]. The model that is of particular interest to the topic of this review is the model proposed by Morrison et al. [55]. This model provides instructional designers with flexibility in determining the design steps to be taken and places significant emphasis on selecting the delivery mode, including considering technology’s potential role Obizoba et al. [58].

**Fig. 1:**
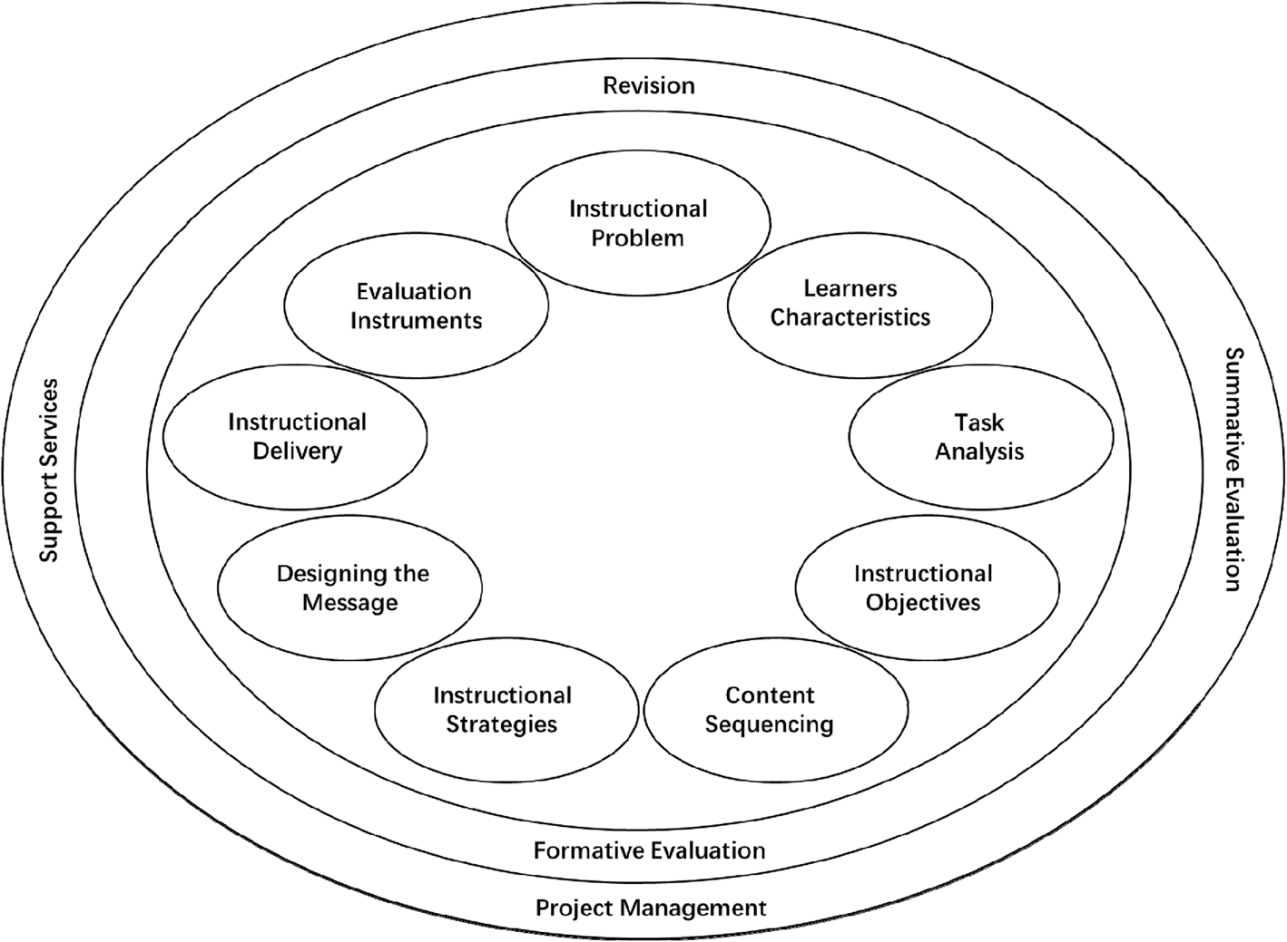
Instructional Design by Morrison et al. [55]

Starting from essential elements to be taken into account when planning instructional design (learners, objectives, methods and evaluation), the Morrison et al. [55] stipulates a circular design process consisting of nine elements: instructional problems, learner characteristics, task analysis, instructional objectives, content sequencing, instructional strategies, designing the message, instructional delivery, and evaluation instruments (Fig. 1).

In Table 1, the elements of this models have been set alongside the ADDIE model showing analyze, design, develop, implement and evaluate. The design of the Morrison et al. [55] model is purposefully circular, signaling flexibility in terms of the order of elements on which to work on rather than prescribing a rigid linear process. Furthermore, the nine elements are considered to be interdependent Obizoba et al. [58] [3]F. Placed around these nine elements are formative evaluation and revision, as well as planning, project management, summative evaluation and support services [55].

**Table 1:** Key Elements of the Morrison et al. [55] Instructional Design Model set alongside the ADDIE model

**Analyze**	**Instructional problems**	Determine the specific goals, and also identify potential instructional issues
	**Learner characteristics**	Identify characteristics of learners that should be taken into account during the planning process
	**Task analysis**	Clarify course content, and analyze the proposed task components in relation to the stated goals and purposes of the course
	**Instructional objectives**	Define instructional objectives and desired learning outcomes
**Design**	**Content sequencing**	Ensure that content for each instructional unit is structure sequentially and logically to facilitate learning
	**Instructional strategies**	Design instructional strategies to enable individual learners to master the content, and achieve desired learning outcomes
	**Designing the message**	Plan the instructional message and the appropriate mode of delivery
**Develop**	**Development of instruction (instructional delivery)**	Develop evaluation instruments suitable for measuring and assessing learners’ progress towards achieving course objectives
**Evaluate**	**Evaluation instruments**	Choose the appropriate resources that will support both teaching and learning activities

### The purpose of the study

There are a number of review studies that explore the application of AR/VR/MR in healthcare education and training. These studies primarily concentrate on evaluating the effectiveness of these technologies in learning [10], comparing their effectiveness with conventional or other teaching methods (as studied by [45]), and examining the prevailing trends in this field (as reviewed by [31]). Currently, there is lack of insight on the application of an instructional design model or instructional theories for the design of education with the integration of AR/VR/MR into education, particularly in health professions education. The first objective of this scoping review is to identify the usage and the potential benefits of including AR/VR/MR tools for education and training of students and professionals in the health domain. Therefore, we will provide a global overview of how AR/VR/MR tools are applied in health professions education and training with regard to the distribution over time, domains, methodologies, rational, outcomes, and findings. The second objective is to investigate whether any instructional design models or instructional theories have been applied when using these tools in designing education. We mapped the results based on the Morrison et al. [55] model. No other review was found that had considered instructional design theories or models guiding the use of AR/VR/MR for teaching in health professions education considering the recent time frame. To fill that gap in the literature, in this study we located and then analyzed all of the peer-reviewed studies in the mentioned databases in the methods section. The purpose is to present a review of the literature on how AR/VR/MR are used in healthcare educational settings from 2015 until 2020. Therefore, with regard to the use of AR/VR/MR in healthcare education and training, the following research questions (RQ) are addressed:

- **RQ1:** What is the distribution over time of the selected studies?
- **RQ2:** Which domains of healthcare and what types of participants are addressed?
- **RQ3:** What type of (instructional) design/methodologies are used? **(**Instructional design aspects + educational theories), how do they map on the Morrison et al. [55] model?
- **RQ4:** What is the rationale behind the exposure to AR/VR/MR?
- **RQ5:** What types of learning and behavioral outcomes (based on Blooms taxonomy) are encouraged?
- **RQ6: **What are the findings of the selected studies?

## Method

In this study, we have conducted a scoping review following the framework proposed by Arksey and O’Malley [7] . The purpose of this scoping review is to map the existing literature on the topic, identify key concepts, sources of evidence, and gaps in the research. The process began with identifying the research question, followed by identifying relevant studies through a comprehensive search of databases such as PubMed, Web of Science, and other publishers. An iterative selection process was used to determine the inclusion and exclusion criteria, and the selected studies were charted based on their key characteristics and findings. The results were then gathered, summarized, and reported.

This scoping review specifically aims to explore the benefits of using AR/VR/MR tools in health education and training. It will also investigates the application of instructional design models or theories in designing education with these tools.

### Databases searched

The electronic databases searched in this review were a set of databases accessible through Libsearch, which is the search engine available through our University library. The databases available through this search engine are: WorldCat.org, Web of science, MEDLINE, SpringerLink, ScienceDirect, Wiley Online Library, Taylor and Francis Journals, ERIC, BMJ Journals, and Sage journals.

Our research focused on papers published from 2015 through the end of 2020. We selected only peer-reviewed papers written in English.

Our data collection was completed before the COVID-19 outbreak, and due to the significant impact of the pandemic on the nature of studies conducted, we deliberately excluded papers published in 2021 and beyond. A preliminary review revealed that the methodologies of studies during this period underwent significant changes. This would have necessitated substantial modifications to our research questions. Consequently, we made the decision to confine our research to the year 2020.

### Search terms

The databases were searched using key terms related to virtual, augmented and mixed-reality as well as terms for possible usage of these devices in medicine, health and bio-medical education. The following search string was used:

[("virtual reality" OR "augment* reality" OR "mixed reality") AND (health OR health science* OR medicine OR "medical science*" OR biomed* OR "biomed* science" OR “life science*”)].

### Search for education and training in medical, biomedical and health sciences

**Fig. 2:**
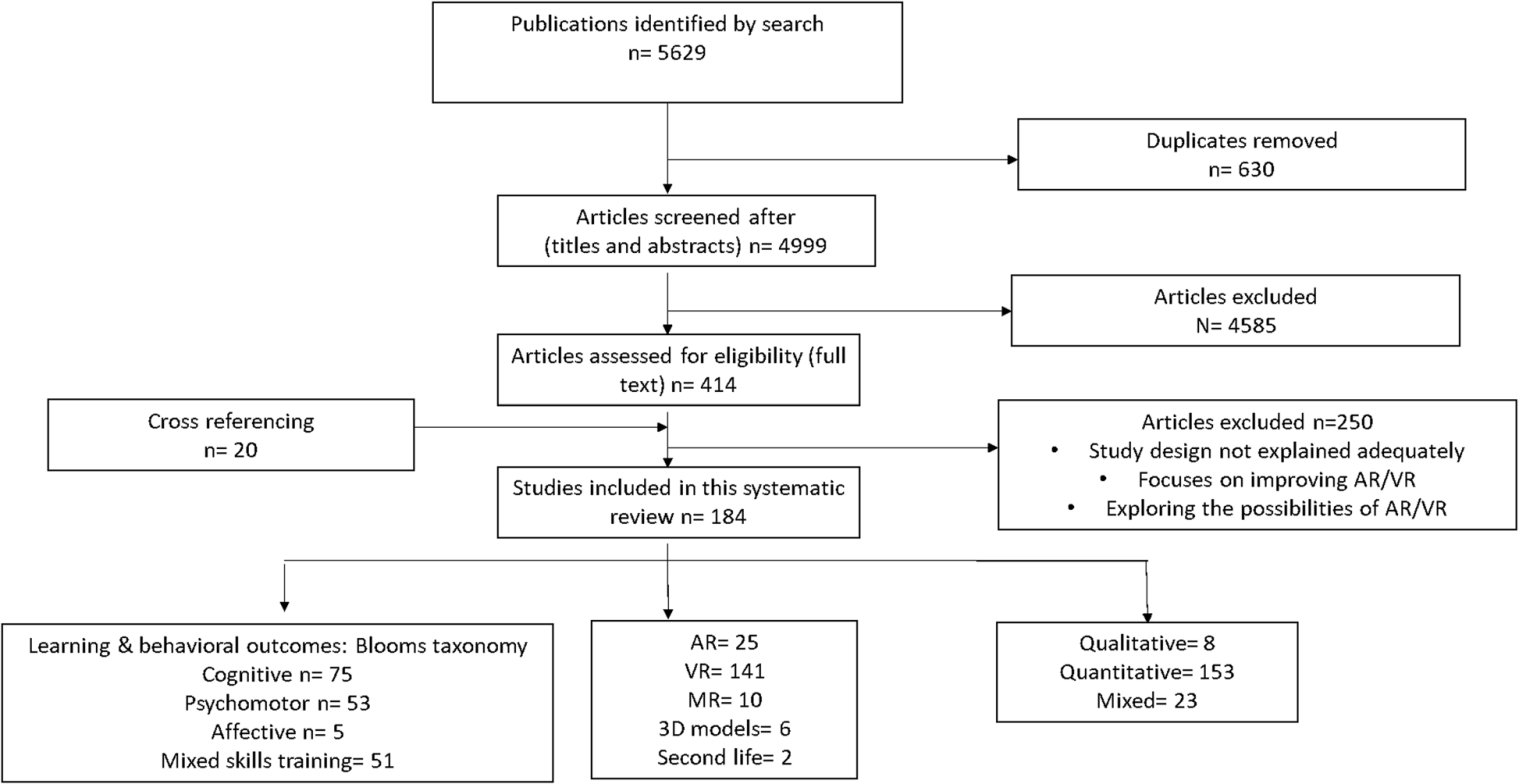
Flow chart showing the screening process

The search returned a large number of papers *n* = 5629 (Fig. 2). This set was further screened by manually going through all titles and abstracts for relevant terminology like “AR, VR or MR,” “training,” “education,” “medical,” “biomedical,” and “health sciences”. Papers selected on this basis were collated and duplicates removed (*n* = 414).

### Selection of papers for inclusion in the review

To select the appropriate studies for inclusion in the review, the full papers (*n* = 414) and the additional papers (*n* = 20) retrieved via cross referencing were screened and a number of further criteria were applied. Selected papers had to (a) include empirical evidence related to the use of AR/VR/MR in education and training, (b) the training had to be in the field of medicine, biomedical sciences or health sciences. The PICOS (population, intervention, comparison, outcome, study design) framework [54] guided the inclusion and exclusion criteria of this study (Table 2).

**Table 2:** Inclusion and exclusion criteria

	**Inclusion Criteria**	**Exclusion Criteria**
Date Range	Between 2015–2020	literature published before Jan 1, 2015, and after Dec 31, 2020
Literature Type	Peer-review (original) studies published, Empirical papers	Literature not in English
Study Methodology	No restrictions	Literature reviews, meta-analyses, opinion papers; non-English literature;
Study Design	No restrictions	
Study Method	Empirical papers	Tool use and testing tool reliability and validity
Setting	Medicine, biomedical sciences or health sciences	Not higher education, not in the field of Health
Participants/Population	Health professionals who received education	Non- education design teams Co-creation teams without participants
Educational Outcome	The article describes educational outcome measure(s)	No description of an educational outcome measures
Publication	Peer-reviewed journals, proceedings of conference posters and presentations, book chapters	Documents from unknown/ unreliable sources

### Coding of selected papers

The papers selected on the basis of the inclusion criteria were coded. To summarize, papers were coded with respect to:

- the publication year;
- the type of participants addressed in the study;
- which one of the AR/VR/MR was used for teaching/learning;
- the country and continent where the first author of the paper was based;
- behavioral outcomes based on Bloom’s taxonomy: cognitive, affective or psychomotor skills;
- the domain of healthcare that AR/VR/MR has been used: neurosurgery, endoscopic surgery, etc.;
- what type of (instructional) design/methodologies are used? (Instructional design aspects + educational theories);
- the rationale behind using AR/VR/MR for training: whether the AR/VR/MR could offer an environment that could overcome the current limitation. For examples, overcoming limitations on teaching surgical steps, or teaching and practicing psychomotor and cognitive skills, etc.;
- variables related to the study: the research design used in the study, categorized as a randomized control trial (RCT); quasi-experimental; survey; correlational or qualitative design; and
- the findings of the selected studies.

### Quality of the studies

Papers were assessed according to the following criteria: (1) quality of research design: RCT; quasi-experimental controlled study, pre-test/post-test design (an explicit research design had to be present, not just reports on a tool); (2) relevance of the aim of the study for using AR/VR/MR and (3) findings of the study (did the findings of the paper really relate to education/some sort of learning? Were the participants really doing something to learn, rather than for example only testing the tool? Was it used to teach someone to do something?

### Consistency and reliability of coding

All authors took part in the identification, coding and quality coding of papers but, for consistency, one of the researchers (MA) oversaw all the coding. A first sample of articles was taken to discuss and align the coding. Subsequently, regular meetings were scheduled between the authors to discuss the papers and their coding.

## Results

**Fig. 3:**
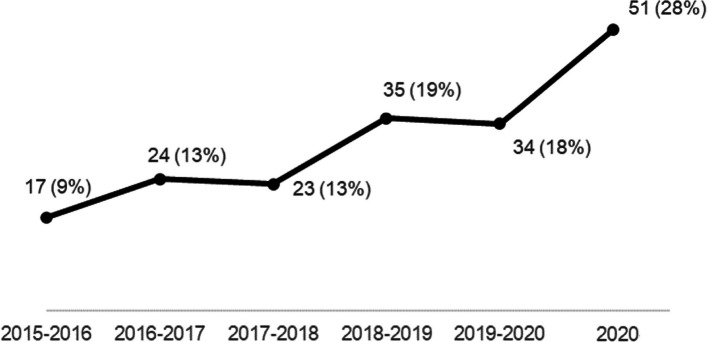
Distribution of the studies from 2015 till end of 2020

The systematic search identified a total of 5629 articles (Fig. 3). After removing duplicates, 4999 articles were screened for relevance based on title and abstract. As a result, 4585 articles were excluded, leaving 414 articles for full-text review. Cross-reference search identified 20 more articles to be eligible. After full-text review, a total of 184 articles remained relevant for inclusion.

### Distribution of studies over time

Overall, the number of studies including AR/VR/MR in health education, seems to be increasing. A total of 17 (9%) of the 184 articles included in our review were published in 2015; 24 (13%) of the articles were published in 2016, and 23 (12%) in 2017. In 2018, 35 (19%) articles were published, in 2019, 34 (18%) and in 2020 there were 51 (27%) articles. Figure 3, depicts a rise in the number of studies per year from 2015 till end of 2020.

### Domains of healthcare and types of participants

**Fig. 4:**
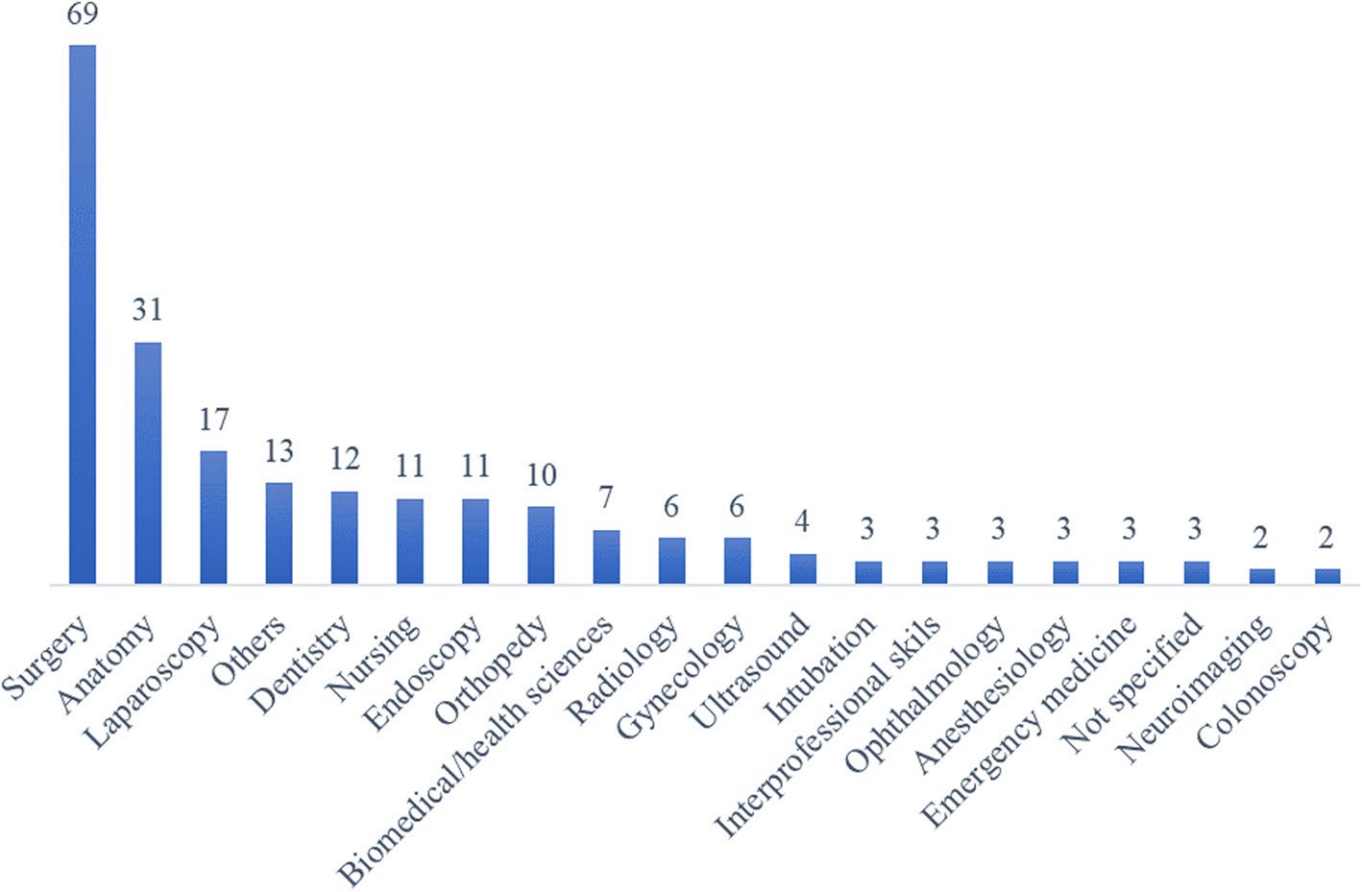
Domains of healthcare—categories mentioned here are not mutually exclusive, they can overlap and intersect with one another

Most research studies primarily explored the application of AR/VR/MR technology in the medical field, specifically for training medical and nursing students in surgical procedures and anatomy courses. However, a limited number of studies investigated other healthcare domains. For instance, twelve studies specifically examined dentistry, while seven studies included biomedical and health sciences students alongside medical students. For the studies focusing on medicine, the majority of uses for AR/VR/MR in teaching was for training surgical skills (Fig. 4). Most the surgeries were mainly related to minimally invasive surgeries, like endoscopy, laparoscopy, etc. When counting all the research related to AR/VR/MR in surgery, which also included the research in fields like endoscopy, laparoscopy, etc. we ended up with 69 papers (Fig. 4). A second common use for AR/VR/MR in medical education was to teach anatomy, *n* = 31 papers (Fig. 4). The focus of these studies were on neuroanatomy, 3D learning structures, and improving visual ability on anatomical understandings.

In the comprehensive analysis of the studies included, a diverse spectrum of student levels is addressed. This encompasses bachelor students, master’s students, residents, and specialized continuous education. Notably, certain studies also delve into student training programs and multi-level training sessions, which involve a combination of students, residents, and expert specialists (Table 3).

**Table 3:** Study population (studies may involve more than one type of population)

Study population	Studies (*N* = 184), n (%)
Students	95 (51.7%)
Bachelor	84 (45.7%)
Master	11 (6%)
Residents	14 (7.6%)
Physicians/ specialists	53 (28.8%)
Mixed training levels	22 (12%)

**Fig. 5:**
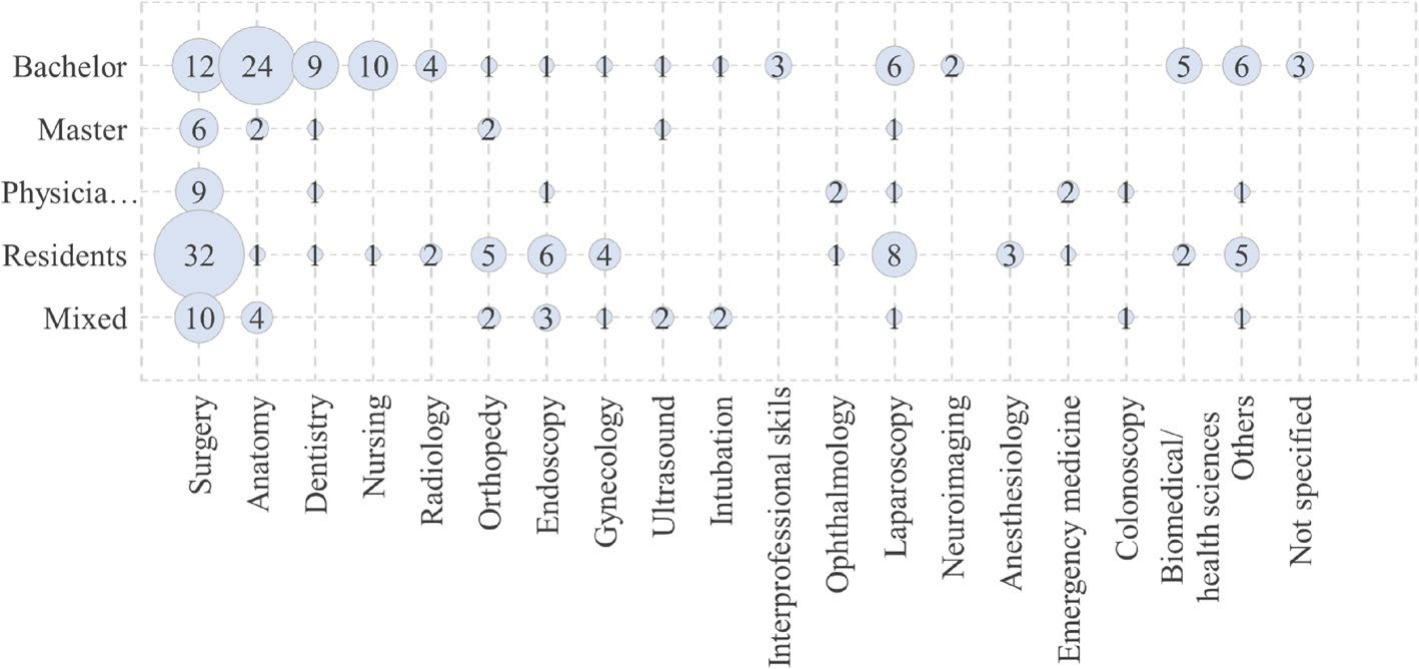
A visual representation of the study domains and population

The bubble chart in Fig. 5 links study domains and population. As evident, most studies are related to training residents’ surgery skills (*n* = 32) and to teaching anatomy to bachelor students (*n* = 24). The coded number of papers based on the domain and population can be found in Appendix 1, Table A. The reference to the codes can be found in Appendix 2.

### Types of Study design/methodologies

**Fig. 6:**
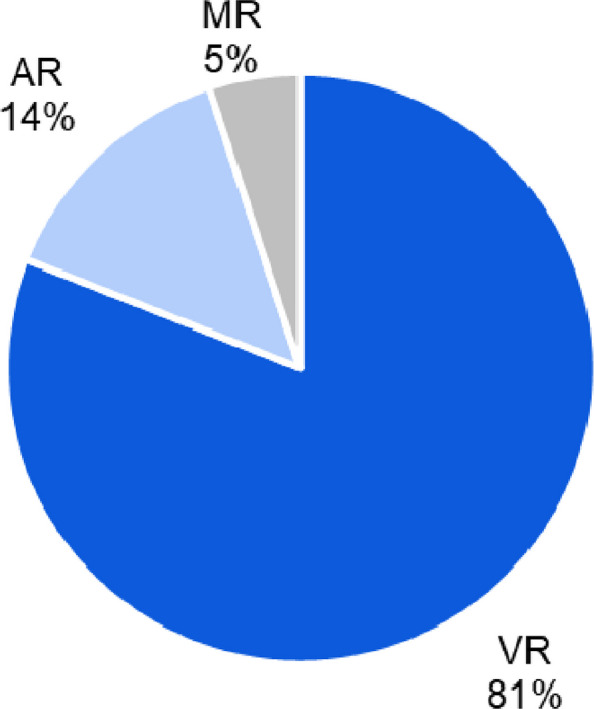
Distribution of research focus across VR, AR, and MR

For consistency, we took the terms AR, VR or MR used by the authors of the original papers to make our classification. As shown in Fig. 6, the large majority of studies (*n* = 149; 81%) focused in VR, followed by AR (*N3* = 25; 14%) and MR (*n* = 10; 5%).

**Fig. 7:**
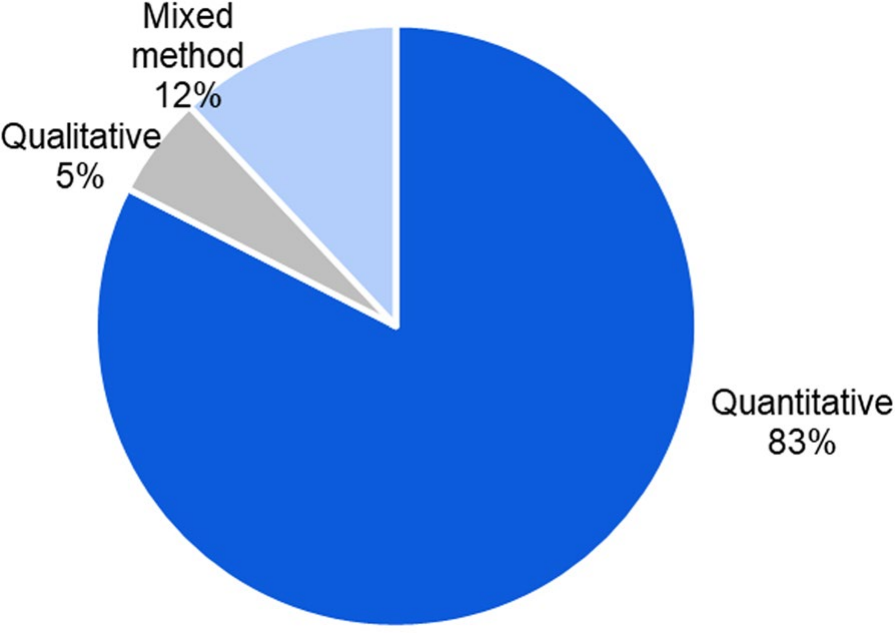
Distribution of research methodologies: quantitative, mixed-methods, and qualitative

We divided the articles and distinguished between studies with qualitative, quantitative or mixed designs. Large majority of studies used a quantitative methodology (*n* = 152; 83%), followed by mixed-methods designs (*n* = 22; 12%), and there were only a very small number of qualitative studies (*n* = 10; 5%) (Fig. 7).

**Fig. 8:**
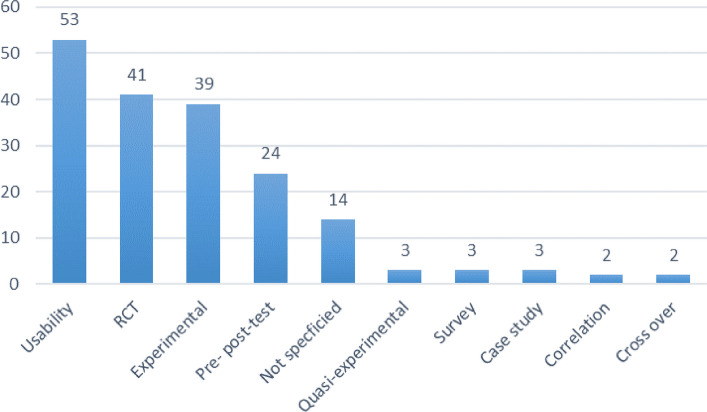
Types of study methodologies in percentages

In Fig. 8, you see that most studies focused on usability aspects of AR/VR/MR (*n* = 53, 29%). Their purpose was typically to see if these tools could be used for a particular purpose, and mostly to check all the functions of the tool. The second most common study methodology is Randomized Controlled Trial (RCT) (*n* = 41, 22%).

**Fig. 9:**
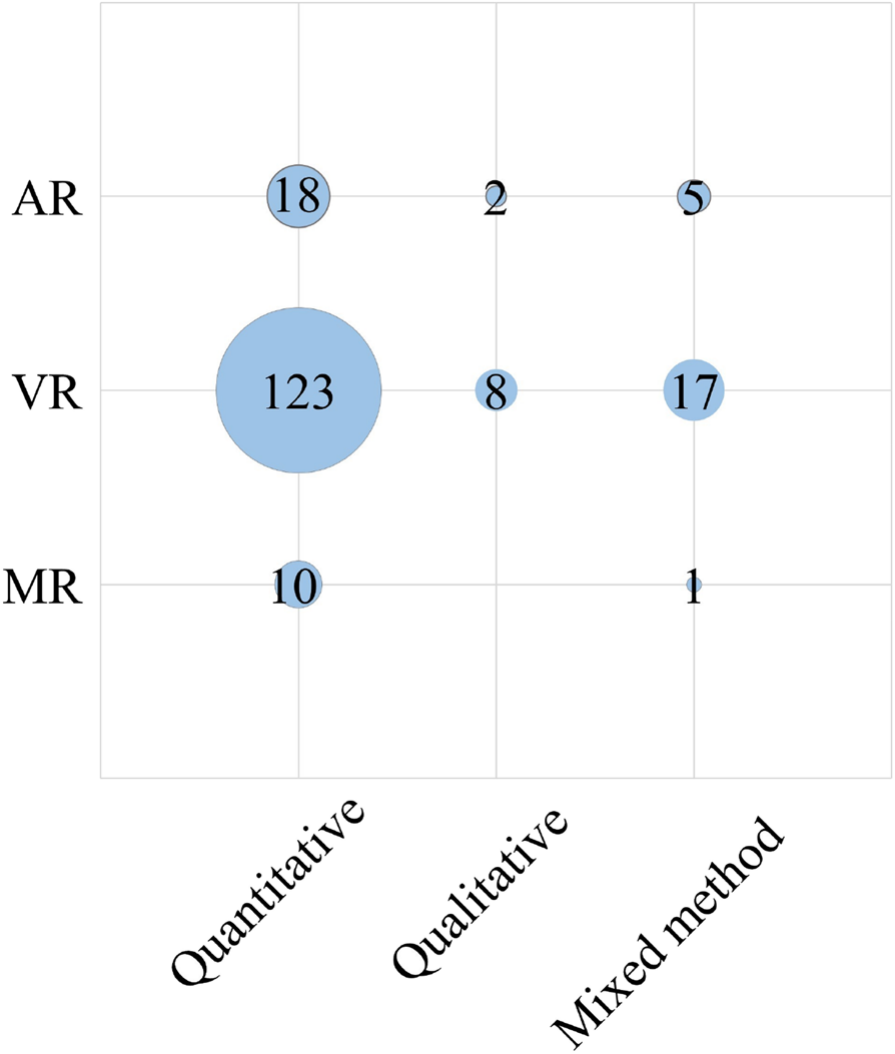
Distribution of study designs by technology mode

To plot the study design against the mode of technology used, Table B in Appendix1, was prepared. The reference to the coded papers can be found in Appendix 2. Figure 9, clearly shows that 123 papers used VR in quantitative study designs. Eighteen papers used AR in quantitative study designs and 17 studies used VR in mixed method research designs.

**Fig. 10:**
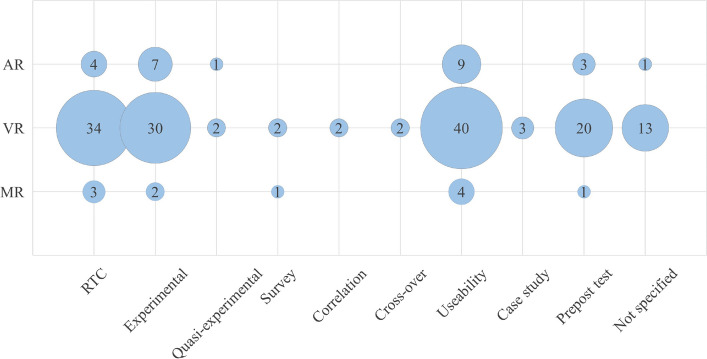
Plotting distribution of study methodologies by technology mode

To plot the study methodology against the mode of technology used, Table C in Appendix 1 was prepared. Coded papers in Table C can be found in appendix 2. Figure 10 clearly shows that 40 studies used VR in usability studies, 34 studies used VR in RCT research methodologies and there were 30 experimental studies with VR.

### Instructional design aspects and educational theories used in these studies

Looking at instructional design and educational theories in combination with AR/VR/MR, we see that only 44 studies out of the total of 184 had something mentioned about theories or instructional designs that they used for designing their teaching and learning. Interestingly, some studies specifically investigated usability aspects of AR, VR, or MR in medical education but did not incorporate any explicit educational design theory. This underscores the need for intentional integration of instructional design principles and educational theories when implementing these immersive technologies in educational settings. Table 4 displays the different theories that some studies applied for their educational design. These theories have literally been mentioned in the studies by the authors (Table 4). Among them, self-directed, competency-based and PBL, and evidence-based learning were most commonly used.

**Table 4:** Educational design theories applied in studies

**Instructional design aspects/educational theories**	**Study ID**
Serious game with a learner-center teaching approach	5,103,181,159
Proficiency-based training	2,108
Procedure-based training	3
Motivation & self-regulated learning	6,23,61,70
Self-paced and self-directed learning	16,85,145,176,183,169
Autonomous learning	21
Cognitive theory of multimedia learning	24,98,173
Cognitive load (CL) theory	24,37,127,150
Mastery learning	59
Skill acquisition theory Fitts and Posner	49
Persistent active learning	95
Experiential learning	71,167
Self-determination theory (SDT)	120,119
Evidence based learning	107,131,121,184,149
Collaborative Learning	113
Competency-based/PBL	29,124,110,172,141,169
	**Total 44 (184)**

In Table 5, we tried to link the already existing theories to the underlying elements in an instructional design theory. Here the Morrison et al. [55] was a good match. The purpose was to show how an instructional design model and, in this case, the different elements of the Morrison et al. [55] model, could be used as guidelines in designing courses with AR/VR/MR in medical education. We especially looked at the design element in the Morrison et al. [55] model. We hope to reveal some guidelines for including instructional design aspects when planning to use AR/VR/MR in medical education. While Table 5 clearly indicates that only a limited number of studies have taken instructional design elements into account, it’s worth noting that a small subset of studies did indeed consider these aspects. For example, code 141 is a study by Chheang, et al. [15], they are relying on instructional strategies like problem-based learning, hoping that these strategies would open new directions for operating room training during surgery. We also see, in the study by Liaw, et al. [47], (code 113), that VR has been used as an instructional strategy for collaborative learning across different healthcare courses and institutions in preparing for future collaborative-ready workforces. Another example can be the way VR is used in course design and in relation to cognitive load. Vera, et al. [75], (code 127), show that a certain VR operating tool can be integrated in the residency program which is sensitive to residents' task load, and it could be used as a new index to easily and rapidly assess task (over)load in healthcare scenarios. In another research, (code 24), Küçük, et al. [44] designed a study to determine the effects of learning anatomy via mobile AR on medical students' academic achievement and cognitive load.

**Table 5:** Instructional design aspects/educational theories that were used in the different studies, plotted against Morrison et al [55]

**ID aspects/educational theories**	Competency-based/PBL		141		29,110
	Collaborative Learning		113		
	evidence based learning		131	184,149	
	self-determination theory (SDT)				120
	experiential learning			71,167	
	persistent active learning		95		
	skill acquisition theory Fitts and Posner			49	
	Mastery learning				
	Cognitive load (CL) theory		37	24,127,	
	cognitive theory of multimedia learning		173	24	
	autonomous learning		21		
	self-paced and self-directed learning		145	85,176,	
	Motivation & Self-regulated learning	70			6
	Proficiency-based training				2
	Serious game (learner-center teaching approach)			5	
		**Content sequencing**	**Instructional strategies**	**Designing the message**	**Instructional delivery**
		**Design**			**Delivery**

### Rationale behind using AR/VR/MR in healthcare education

The predominant motivation behind incorporating AR/VR/MR (Augmented Reality, Virtual Reality, and Mixed Reality) in healthcare education was to address specific limitations. These common limitations included factors such as the absence of realism, the financial burden associated with maintaining real-life props, time constraints, the need to simulate complex scenarios, ensuring a safe and controlled practice environment, managing cognitive load, and facilitating repetitive training opportunities (Table 6). For example, VR was used as an alternative to plastic or cadaver models, which were mentioned as being subject to a lack of realism and pertaining high maintenance costs, respectively [1, 8]. Furthermore, learners in the wider healthcare field, often needed many hours of practice to master a skill, AR/VR/MR were good examples to provide an efficient field for practice. In some specialties, VR was specifically used because it provided the possibility to set up highly complex scenarios at a low cost. Through the use of VR, these limitations could be overcome and practice could be provided in a safe, controlled setting [29]. In a similar vein, some studies mentioned that they would use VR to reduce students’ cognitive load [16, 44], by manipulating some aspects of the task over others. The ability to manipulate aspects of the task can be useful for both training and assessment.

**Table 6:** Rationale behind using AR/VR/MR

Rationale behind using AR/VR/MR -Overcoming the common limitation:	Study ID
Lack of realism (Immersive training)	1,2,8,20,28,32,36,50,51,58,65,76,83,85,86,91,117,118,120,158,168,171,176
High maintenance costs (Increased cost-efficiency)	5,8,15,24,26,32,50,60,147,153,164
Time limitation (Increased time-efficiency)	24,29,30,35,39,47,61,89,99,101,105,112,114,166,169
Practicing high complex scenarios (Feasible education tool)	5,13,26,29,30,34,64,66,67,75,99,102,107,113,128,132,147,155,
Providing the safe/controlled setting	2,4,9,13,19,55,56,65,74,79,87,88,89,91,108,152,160,163,179
Reduce cognitive load	22,24,37,41
Reduce errors	12,19,57,
Possibility of repetitive training	74,82,95,170,
Face and content validity	43,58,92,142,
Improve students’ motivation	6,21,23,60,84,119,
Self-directed learning	6,26,70,95,98,138,145,
Higher engagement	59,71,80,81,115,124,133,148,173,183,
Improve observation	42,45,46,63,84,139,
Increased accuracy and precision	11,20,29,53,94,130,149,181,182,
Better performance compared to conventional methods	27,31,140,
Learning in unsupervised setting	3,9,25,62,78,96,100,110,143,
More attentiveness	81,126,
Not specified	7,10,14,16,17,18,33,38,40,44,48,49,52,54,68,69,72,73,77,90,93,97,103,104,106,109,111,116,121,122,123,125,127,129,131,134,135,136,137,141,144,146,150,151,154,156,157,159,161,162,165,167,172,174,175,177,178,180,184

Another rationale was to improve students’ motivation [39, 50] and/or self-directed learning [27, 46]. As students are used to using digital technologies in almost all aspects of their lives, using these technologies in education was thought to have a positive impact on their perceptions. This rationale was often mentioned for teaching anatomy, which is a course that students often tend to find uninteresting [27, 44].

Moreover, in the context of Augmented Reality (AR), technologies have been employed to enhance student engagement and observation beyond what is achievable under typical circumstances.. For example, AR technologies would be used to overlay information from other modalities (e.g., MRI) on to-be-diagnosed images, making it easier to combine the information in order to locate abnormalities [12].

We plotted instructional design aspects against the rationale for using AR/VR/MR tools that each research considered for their study design or simulation design (Table 7). Since rationale behind using a specific method or tool comes at the analysis part of instructional design, we took the analysis section of the Morrison et al. [55]. The purpose is to see how relying on the analysis section of an instructional design model can help with logically designing the rationale behind using a tool operated by AR/VR/MR in health education.

**Table 7:** Rational behind using AR/VR/MR and Morrison et al. [55]

**Rational behind using AR/VR/MR**	More attentiveness			81	
	Learning in unsupervised setting	62,		96	78
	Better performance compared to conventional methods				31,
	Increased accuracy and precision			29, 94,	20, 53,
	Improve observation			42	
	Higher engagement				59
	Self-directed learning		26,	70,	
	Improve students’ motivation			23	21
	Face and content validity				43
	Repetitive training				
	Reduce cognitive load			22, 24, 41,	37,
	Safe/controlled setting		19	19, 89	
	Feasible education tool	30	102,		75, 113
	Increased time-efficiency	61		89, 101,	
	Increased cost-efficiency	32,			
	Immersive training	28		36, 76, 86,	20, 58,
		**Instructional problems**	**Learner characteristics**	**Task analysis**	**Instructional objectives**
		**analyze**			

The available data shows that some studies considered the learner characteristics by having two groups with different knowledge levels (novice/expert) and compared their performance [22],code 19). Some provided immersive training as an instructional objective to improve face and content validity [24],code 20). Some others utilized simulation in order to improve student’s motivation [27],code 21). Some considered task analysis by providing tasks at different simulations [28, 39],codes 22, 23). In other studies, simulation was used for personalized and self-directed learning [50],codes 26) and some attempted to resolve the issues, difficulties and disadvantages of current methods [53],code 28).

### Types of learning and behavioral outcomes

**Fig. 11:**
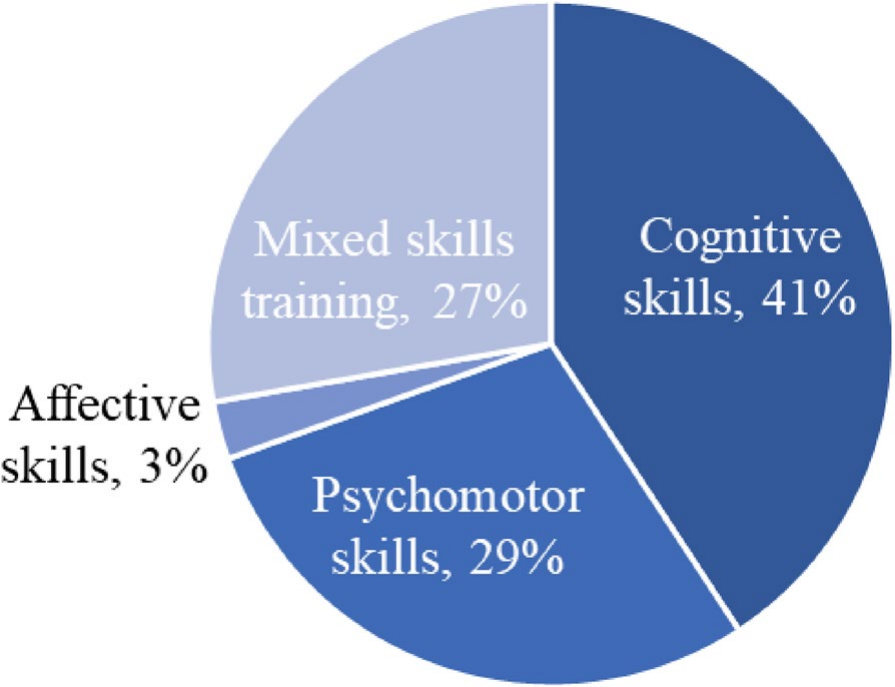
Types of learning and behavioral outcomes

The AR/VR/MR articles were divided into the different learning and behavioral domains. According to Bloom’s revised taxonomy [5], three domains can be distinguished: the cognitive, affective and the psychomotor domain. The cognitive domain refers to the mental processes needed to engage in (higher-order) thinking. The affective domain refers to development of students’ values and attitudes, while the psychomotor domain has to do with developing the physical skills required to execute a (professional) task [5]. Of the included studies, seventy-five used AR/VR/MR for teaching cognitive skills (41%, Fig. 11. Psychomotor skills were targeted in 53 studies (29%, and 5 studies (3% focused on affective outcomes aiming at improving learners’ confidence in surgery; especially, training in neurosurgery, laparoscopy, orthopaedic, endoscopy, sinus surgery, bone surgery, electro-surgery, and eobotic surgery. It is also interesting to know that fifty-one studies (27%) utilized a mixed skills training.

### Outcomes of the studies that used AR/VR/MR in healthcare education

**Fig. 12:**
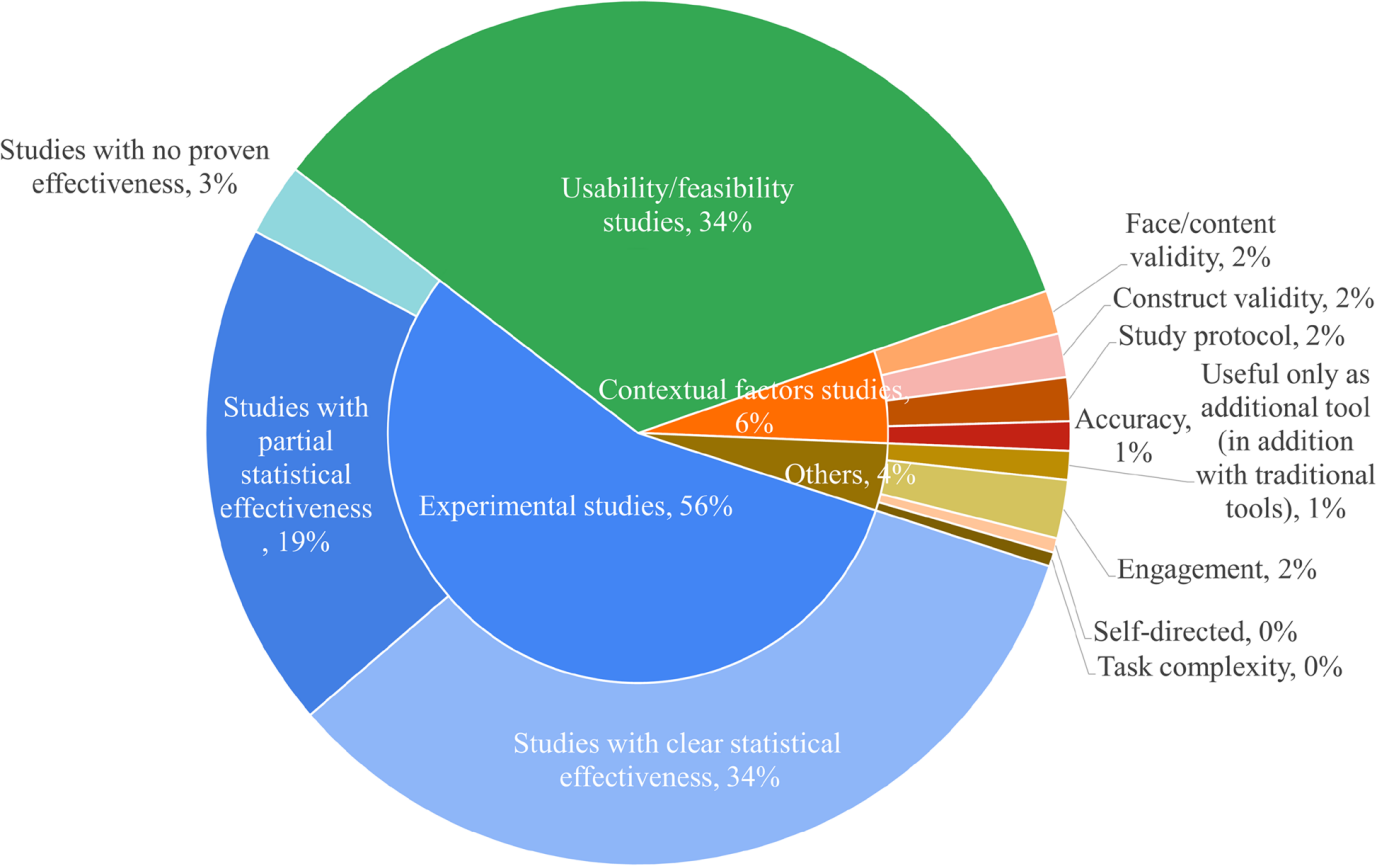
Effectiveness of included studies

The included studies in this review generally categorized an intervention as effective if the majority of the participants achieved significantly higher scores in tests (experiment/control, pre-posttest, exercises) compared to traditional instructional approaches, such as analogue surgery or ultrasound procedures (Table 8). Up to 56% of the studies were experimental studies (Fig. 12).

**Table 8:** Outcomes of included studies

**Effectiveness**	Study ID
**Experimental studies:** Studies with statistical effectiveness	2,3,6,11,12,17,19,22,23,24,27,28,30,35,39,40,42,47,49,51,52,53,56,57,66,67,85,87,88,89,93,94,95,96,98,100,102,103,105,109,123,125,128,131,134,136,137,139,140,144,151,156,158,160,166,172,173,178,180,181,182,184
Studies with partial statistical effectiveness	4,7,9,10,18,25,29,33,37,38,41,44,45,59,60,61,62,64,69,70,71,72,75,76,82,83,84,86,101,129,153,163,169,170,175
Studies with no statistical effectiveness	54,112,114,116,138
**Usability/feasibility studies**	8,20,21,55,63,99,115,124,146,152,167,168,1,15,26,31,32,34,36,50,65,68,73,74,77,78,79,80,81,90,91,92,97,104,106,110,111,117,118,119,130,133,135,141,143,145,148,154,157,159,161,162,164,165,171,174,176,177,107,113,132,147,155
**Contextual factors studies:**	
Face/content validity	13,43,142
Construct validity	58,120,126
Study protocol	48,108,122
Accuracy	46,149
**Others**	
Useful only as additional tool (in addition with traditional tools)	150,183
Engagement	5,14,121,179
Self-directed	16
Task complexity	127

Some studies were considered as partly effective (Table 8), when there were no significant differences in all participants scores (19%, Fig. 12) (e.g. [17, 35],Van Nuland et al., 2016; [76]). Here, differences among the participating groups in the studies could be attributed to the level of the training or expertise of the learners (e.g., [33]). Although in some of these studies, students using the more traditional approaches were performing at the same level as the students in the AR/VR/MR group, there were partial differences reported that learning with AR/VR/MR improved aspects like time efficiency, or precision sensitivity (e.g., [52, 64, 73, 74]).

Some studies did not report any effectiveness (3%, Fig. 12). Study by Llena et al. [49] showed that although students experienced the AR technology as favorable, no significant differences in learning were found between group learning with AR compared to the group learning with traditional teaching methods. In the study by Huang et al. [40], no differences were found between students learning with a VR model versus a traditional physical model.

There were also studies showing mixed results, with some but not all outcomes improving in the AR/VR/MR conditions (e.g., [68]). Other studies reported the positive effects of applying AR/VR/MR as usable (e.g., [41, 51],Van Nuland et al., 2016), feasible (e.g. [67]) tool for healthcare training (e.g. [47, 72, 75, 76]). Few studies considered contextual factors like face/content validity (e.g. [30, 63]), construct validity (e.g. [1, 21, 22, 56]), study protocols [4], and accuracy (e.g. [12, 43, 60]).

Several studies reported on variables that impact the effectiveness of AR/VR/MR technologies. One commonly mentioned variable was level of expertise: learners/practitioners with more experiences and/or years of training outperformed novices (e.g., [37]), and experience had a positive effect on skills acquisition when using these technologies (e.g., [44]). An exception to this was the study of Hudson et al. [42], in which nurses with more years of practice found it more difficult to use the technology. Furthermore, Lin et al. [48] reports an effect of gender, in which men tended to reach proficiency sooner than women when using a laparoscopic surgery simulator. Nickel et al. [57] further indicated that experiencing fun was also relevant for the student’s learning. In the study by Huber et al., [41] were they investigated the use of VR to improve residents’ surgery confidence, a correlation was found between confidence improvement and students’ perceived utility of rehearsal. In the same study, the authors showed that the effect of the rehearsal on learner’s confidence was further dependent on trainees’ level of experience and on task difficulty. Finally, Chalhoub et al. [14] found that gamers had an advantage over non-gamers when using a ‘smartphone game’ to learn laparoscopic skills in the first learning session, although all participants improved in a similar manner.

## Discussion

In this comprehensive review of literature, we explored the application of AR/VR/MR technologies in the instruction of various stages of medical and health professions education. We identified six key research questions to guide our investigation: 1) the trend of studies over time, 2) the healthcare domains and participant types included in these studies, 3) the design methodologies and instructional design aspects/educational theories employed in these studies, 4) the benefits and underlying reasons for using AR/VR/MR in medical and health professions education, 5) the kinds of learning and behavioral outcomes promoted by the use of AR/VR/MR in this field, and 6) the results regarding these learning outcomes in studies that examine the use of these technologies in medical and health professions education.

In general, we observed a rising trend in the number of studies focusing on the application of AR/VR/MR in medical and health professions education. This suggests a consistent and growing interest in leveraging these technologies to enhance student learning across various healthcare disciplines. The primary use of these tools was found to be in teaching surgical skills to residents and anatomy skills to undergraduate students.

When examining the research methodologies employed to study the integration of AR/VR/MR, a notable finding was the predominant focus on quantitative methodology. However, given the limited number of participants in programs such as residency or professional training, qualitative methods could offer researchers the opportunity for a more comprehensive analysis of these tools’ usage and provide detailed insights into these complex learning situations [2, 18].

It is interesting to note that the study of affective outcomes is often overlooked when integrating AR/VR/MR into health professions education. While studies are typically categorized based on cognitive, psychomotor, and affective outcomes, the majority focus on cognitive aspects, followed by psychomotor outcomes. Only a small number of studies explore the use of AR/VR/MR for teaching affective outcomes.

Usually, when AR/VR/MR is used in contexts related to emotions and affections, it serves more psychological purposes for patients rather than instructional ones [26]. However, there is potential value in using these technologies for specific situations, such as targeting affective outcomes like empathy (e.g., [25]).

In the context of 21st-century multidisciplinary healthcare, prioritizing patient needs and addressing their concerns is crucial. Compassionate and appropriate communication within healthcare teams can build patient trust [23]. To foster interpersonal skills among healthcare providers, it’s important for health professions education programs to emphasize student competencies in the affective domain of learning [20]. Interestingly, despite its importance, this aspect is less explored compared to other applications of AR/VR/MR in health professions education.

In this review, we not only examined outcomes but also scrutinized the findings from the included studies. These findings were grouped into three categories: experimental design, usability studies, and contextual factors (Table 8). Interestingly, not all experimental studies demonstrated effective outcomes for the application of AR/VR/MR in medical and health profession education. Some studies argued that display technologies did not significantly enhance learning across all or most outcome measures (e.g., [14, 17, 21, 35, 40, 49, 69, 76]).

This review also uncovered that only a handful of studies built their AR/VR/MR applications based on specific instructional design models or theories, and there is little description on how these applications can be incorporated into the teaching curriculum. As mentioned in the introduction, instructional design should be rooted in robust theoretical models. Instruction is often ineffective, and knowledge about instructional design needs to be considered to address this issue and optimize complex learning. In other words, the focus should not only be on what is taught but also on how it is taught, which is of paramount importance [38], Reigeluth & Carr-Chellman [66].

We suggest that several factors should be considered when creating educational materials based on AR/VR/MR. In this review, we recommend using the instructional design model by Morrison et al. [55]. When focusing on this model, it is crucial to consider the unique value that a virtual environment can add to enhance students’ learning process when addressing instructional problems and strategies. For instance, AR/VR/MR can offer distinct advantages to learning by providing scenarios where patient privacy is crucial Pan, et al. [59] or where standardization is key [43, 67, 74].

Regarding learner characteristics, it is important for learners to be at ease with the general use of technology and specifically for learning. VR can provide a safe environment for both patients and students to practice essential skills (e.g., [8, 29, 33, 57, 60, 63]).

When considering *task analysis*, it’s crucial to understand that all students will be performing the same task, leading to the point of standardization. All participants can practice the same task, allowing teachers to manage what everyone is learning. The tasks can be whole-task problems (e.g., students demonstrating they can conduct a full consultation) [56], or part-tasks (e.g., surgical procedures) [43, 51, 67, 76]. Similar to the instructional problem mentioned earlier, it’s important to consider the objectives of the task before designing the teaching/learning methodologies and applications.

In terms of *instructional objectives*, it is a widely accepted practice in education to clearly define intended learning outcomes (ILOs) prior to designing learning and assessment tasks [11]. This principle holds true for the use of AR/VR/MR in health professions education. As previously mentioned, the application of these technologies should have a specific purpose, rather than being used merely for their “cool” factor or “motivating” qualities (e.g., [17, 27, 39, 49, 50, 69]). The most common justifications found in the studies included in this review were to overcome certain limitations (such as lack of realism, high maintenance costs for real-life props, time constraints, practicing complex scenarios, providing a safe/controlled setting for practice, cognitive load, and the opportunity for repetitive training), to boost students’ motivation, or to enhance students’ observation skills and attentiveness beyond their usual capabilities.

Beyond integration, it’s also crucial to consider where in the curriculum the technology will be most effective, which relates to the aspect of *content sequencing*. This will depend on the course and curriculum content, as well as the intended learning outcomes (ILOs). In terms of assessment tools, these technologies can also be utilized for *evaluation purposes*. Particularly in formative assessment, they can offer learning opportunities coupled with feedback for the users [36].

When discussing all the elements of the Morrison et al. [55] model, it is equally important to consider *instructional delivery*, particularly in terms of the necessary resources and support. For instance, teacher training is crucial, as it can not be assumed that teachers are inherently capable of utilizing the technology. This pertains not only to the technological aspects of the application (how does it operate?), but also to the pedagogical aspects (how should it be implemented in class, and how should students be guided?). With the insights from this research and the recommendations based on the Morrison et al. [55] model, the understanding of new training and practice methods will enable practitioners to choose from a wider range of training options.

## Limitations

This review has several limitations. Firstly, we exclusively examined studies that incorporated an intervention and utilized AR/VR/MR to teach knowledge or skills to the healthcare professions population. We ignored all theoretical papers. There might be more discussions in theoretical papers on the use of different educational models and theories. Future work might need to include all sorts of studies to cover a broader picture.

Secondly, we limited ourselves to publications between 2015 and 2020, assuming that this would be the timeline when AR/VR/MR gained more popularity in the health education domain.

Thirdly, our study did not thoroughly investigate the limitations and barriers associated with utilizing AR/VR/MR technologies for educational purposes.. When using these technologies in the classroom, it is necessary to acquire the required equipment and to be able to store it safely, both in terms of physical storage of devices as well as cloud storage of data. Batteries may need to be charged and the equipment must be kept clean. Updates may sometimes be required, and it is possible that these will happen at an inconvenient time (e.g., mid-session). Special requirements may be present for the software to run. For example, it might be necessary to make an account in order to be able to use the software, which must then be arranged while also taking into account data protection rules. The space in which instruction takes place should also be considered. For example, is it necessary that students can walk around? If so, this should also be facilitated. Finally, it is worthy of mentioning that none of the named limitations impairs the value of this work, in fact it provides opportunities to more research and further strengthening this topic.

## Conclusion and recommendations for future research

The most important points that stand out when looking at the results of this review are general lack of instructional design theories or models guiding the use of these technologies for teaching and learning, and the abundant use of these tools for teaching courses like anatomy or for designing part-task practice routines in surgery, especially things like offering the possibility of scalability and repeated practice. For the lack of models and theories in course design with AR/VR/MR, we have tried looking at the instructional design model by Morrison et al. [55] and plotting our findings against this model to help guide further studies on how they can use an instructional design model in designing courses that include AR/VR/MR tools.

In general, when looking at the quality of the existing studies and applications including the educational benefits of these technologies, further studies need to be conducted to gain better insight into the added value of including these expensive and sophisticated tools into our education [31]. The most common rationales that were found in the included studies referred to overcoming some sort of limitation (lack of realism, high maintenance costs for real life props, time limitations, practicing high complex scenarios, providing safe/controlled setting for practice, cognitive load and, providing the possibility of repetitive training), enhancing students’ motivation or improving students’ observation and attentiveness beyond their normal capabilities.

## Supplementary Information

Supplementary Material 1: Appendix 1.

Supplementary Material 2: Appendix 2.

## Data Availability

All relevant data are available in the form of appendices.

## Abbreviations

AR: Augmented Reality
VR: Virtual Reality
MR: Mixed Reality
UM: Maastricht University
PubMed: Public Medical Literature
ERIC: Educational Research Information Center
IEEE: Institute Electrical Engineers
SCOPUS: Scientific Content on Public Access
EBSCO: Electronic Book Service Company
RQ: Research Question
ADDIE: Analysis Design Development Implementation Evaluation
PICOS: Population, intervention, comparison, outcome, study design
MEDLINE: Medical Literature Analysis and Retrieval System Online
BMJ British: Medical Journal
RCT: Randomized control trial
MRI: Magnetic Resonance Imaging
