# Supplementary material for: Theoretical foundations and implications of augmented reality, virtual reality, and mixed reality for immersive learning in health professions education
Source: Adv Simul (Lond). 2024 Sep 9;9:36. doi: 10.1186/s41077-024-00311-5 (PMC11382381; doi:10.1186/s41077-024-00311-5)
Supplement: Supplementary file 1 — Supplementary Material 1: Appendix 1. [file 41077_2024_311_MOESM1_ESM.docx]

**Appendix 1**

**Title:** How are Augmented Reality, Virtual Reality and Mixed Reality used for training in Health Professions Education?

**Table A.** Studies distributed by domain and population (for article numbers see Appendix 2)

|  | Bachelor | master | residents | Physicians/ specialists | Mixed |
| --- | --- | --- | --- | --- | --- |
| surgery | 37,82,96,100,105,107,108,112,114,122,160,172 | 48,61,75,90,101,182 | 1,2,3,11,40,46,63,93,99 | 8,9,13,15,17,19,20,22,25,28,29,30,36,38,51,52,55,56,73,74,77,79,83,87,88,92,104,110,123,142,162,168 | 4,12,32,59,68,71,91,95,97,127 |
| anatomy | 6,18,21,23,24,26,33,41,47,54,60,76,80,85,98,117,139,140,144,150,151,154,166,169 | 27,35 |  | 31 | 42,80,106,145 |
| dentistry | 45,84,86,111,125,156,178,183,184 | 163 | 93 | 44 |  |
| nursing | 49,103,109,138,147,148,173,174,179,181 |  |  | 50 |  |
| radiology | 72,115,159,170 |  |  | 65,149 |  |
| orthopedy | 180 | 126,182 |  | 15,22,29,36,158 | 68,71 |
| endoscopy | 114 |  | 99 | 17,20,30,132,135,168 | 4,32,70 |
| gynecology | 128 |  |  | 43,53,74,77 | 120 |
| ultrasound | 62 | 66 |  |  | 7,164 |
| intubation | 175 |  |  |  | 102,129 |
| interprofessional skils | 113,118,176 |  |  |  |  |
| ophthalmology |  |  | 63,89 | 67 |  |
| laparoscopy | 69,82,100,112,122,165 | 90 | 11 | 9,25,51,58,83,87,88,123 | 59 |
| neuroimaging | 81,94 |  |  |  |  |
| anesthesiology |  |  |  | 104,121,141 |  |
| emergency medicine |  |  | 39,57 | 5 |  |
| colonoscopy |  |  | 10 |  | 64 |
| biomedical/health sciences | 116,131,146,157,161 |  |  | 123,136 |  |
| others | 78,137,152,153,155,171 |  | 134 | 14,16,34,167,177 | 133 |
| not specified | 119,130,143 |  |  |  |  |

**Table B.** Study design and mode of technology used

| Designs | AR | VR | MR |
| --- | --- | --- | --- |
| Quantitative | 3,6,21,22,26,46,57,62,75,84,96,98,102,146,149,163,164,183 | 1,2,5,8,9,10,11,12,15,17,19,20,25,28,29,32,35,36,37,39,40,41,42,43,44,45,47,48,49,50,51,52,53,56,58,59,60,61,63,64,67,69,70,74,76,79,81,82,83,85,86,87,88,89,90,91,92,93,94,95,99,100,101,103,104,105,106,108,110,112,114,115,116,117,118,119,120,122,123,124,126,127,128,129,130,131,132,133,134,135,137,138,142,143,144,145,147,151,152,153,154,156,157,158,159,160,162,165,168,172,174,175,177,178,180,182,184,18,23,31,33,38,14, | 7,13,54,77,97,139,166,167,169,171 |
| Qualitative | 121,136 | 16,30,55,141,173,176,181,65 |  |
| Mixed method | 24,73,111,150,80 | 4,27,34,66,68,71,72,107,109,113,125,140,148,155,161,170,179 | 78, |

**Table C.** Study methodology and mode of technology used

| Designs | AR | VR | MR |
| --- | --- | --- | --- |
| RTC | 3,24,57,75 | 11,25,30,37,40,42,45,47,48,59,60,61,71,76,82,88,93,94,95,100,101,103,112,129,131,138,151,158,165,172,182,184,23,33 | 54,139,171 |
| Experimental | 6,46,62,102,149,150,163 | 12,17,19,29,35,58,63,64,67,69,85,87,108,114,116,120,123,126,127,128,132,142,144,153,156,160,168,170,175,178 | 7,169 |
| Quasi-experimental | 21 | 27,49 |  |
| Survey |  | 55,89 | 13 |
| Correlation |  | 32,99 |  |
| Cross-over |  | 180,18 |  |
| Useability | 22,26,73,80,84,96,98,111,164 | 1,4,16,20,34,36,50,51,53,56,68,70,72,74,79,81,83,86,90,91,92,105,106,110,117,118,119,133,134,135,141,145,157,159,161,162,174,177,31,65 | 77,78,97,166 |
| Case study |  | 28,104, 14 |  |
| Prepost test | 136,146,183 | 9,10,39,41,52,66,109,113,115,124,125,130,140,143,147,154,155,173,179,181 | 167 |
| Not specified | 121, | 2,5,8,15,43,44,107,122,137,148,152,176,38 |  |
