# Supplementary material for: Theoretical foundations and implications of augmented reality, virtual reality, and mixed reality for immersive learning in health professions education
Source: Adv Simul (Lond). 2024 Sep 9;9:36. doi: 10.1186/s41077-024-00311-5 (PMC11382381; doi:10.1186/s41077-024-00311-5)
Supplement: Supplementary file 2 — Supplementary Material 2: Appendix 2. [file 41077_2024_311_MOESM2_ESM.docx]

**Appendix 2**

Table 1. Overview of the use of AR/VR/MR in research in 2015 and their methodological quality to train students and professionals in medicine, biomedical and health sciences (n=17).

|  | **Article** | **AR/VR/MR**  **Learning & behavioral outcomes (Bloom’s taxonomy)** | **What is the rationale for AR/VR/MR exposure** | **In which domain of healthcare are AR/VR/MR being used?**  **Who are the participants?** | **What type of design/methodology is used in these studies?** | **What are the findings?** |
| --- | --- | --- | --- | --- | --- | --- |
| 1 |  |  |  |  |  |  |
|  | Abelson, J. S., et al. (2015). Virtual operating room for team training in surgery. *The American Journal of Surgery*, *210*(3), 585-590. | VR  Cognitive/affective skills | We hypothesized that VR software can offer realistic team training environments that overcome some of the current limitations. | Medicine  Surgery  Specialization training (Residence)  A total of 33 participants, including 26 trainees and 7 attendings | Quantitative  Usability study*  Metric data and seven-point Likert scale questionnaire | Metric data revealed that attendings completed the simulation in less time, thus conﬁrming construct validity. Attendings and trainees felt that the simulator was realistic, easy to communicate with, and similar to other non–computer-based, communication-based training modules, thus conﬁrming face validity. However, they did not believe it would improve their performance in the OR, possibly because of the short simulation session. |
| 2 | Azarnoush, H., et al. (2015). Neurosurgical virtual reality simulation metrics to assess psychomotor skills during brain tumor resection. *International journal of computer assisted radiology and surgery*, *10*(5), 603-618. | VR  Psychomotor skills | Cadavers, animal models, synthetic phantoms, manikin based, etc. used for surgical training, have the disadvantages of lack of realism, absence of disease-related pathology and bleeding and no limits on surgical exposure. VR simulators could address some of the limitations in the assessment and teaching of psychomotor skills. | Medicine  Neurosurgical  Specialization training (Residence)  A novice resident in the second year of training and an expert neurosurgeon | Quantitative  Performance metrics | The three tiers of metrics obtained from the NeuroTouch simulator do encompass the wide variability of technical performance observed during novice/expert resections of simulated brain tumors and can be employed to quantify the safety, quality, and efﬁciency of technical performance during simulated brain tumor resection. |
| 3 | Chowriappa, A., et al. (2015). Augmented‐reality‐based skills training for robot‐assisted urethrovesical anastomosis: a multi‐institutional randomised controlled trial. *BJU international*, *115*(2), 336-345. | AR  Psychomotor skills | Training in UVA in an AR environment improves technical skill acquisition with minimal cognitive demand. | Medicine  Urethrovesical anastomosis - robot-assisted surgery  Specialization training (Residence); Specialists (continuous education)  N=52: 22 residents and 30 fellows. | Quantitative  Randomized - Control Trial (RCT) | Participants at three institutions were randomised to aHands-on Surgical Training (HoST) technology group or a control group. The participants who underwent AR-based UVAtraining had significantly better GEARS scores in terms of bimanual dexterity and force sensitivity. |
| 4 | Dharmawardana, N., et al. (2015). Validation of virtual‐reality‐based simulations for endoscopic sinus surgery. *Clinical Otolaryngology*, *40*(6), 569-579. | VR  Psychomotor skills | Traditionally, surgical training was apprenticeship-based after medical school. This method inherently requires many hours of hands on experience in OT. With restrictions in junior doctor working hours and decreased exposure to OT time, it is possible that future surgeons will struggle to reach the same standards as their predecessors. | Medicine  Endoscopic sinus surgery  5 ﬁnal-year medical students, 2 interns, 7 resident medical ofﬁcers (RMOs), 6 registrars and 4 consultants. | Mixed  Usability study  All participants completed an online questionnaire after performing four separate simulation tasks. These were then used to assess face, content and construct validity. | Face validity results had a statistically signiﬁcant (P < 0.05) difference between the consultant group and others, while there was no signiﬁcant difference between medical student/intern and RMOs. Students, interns and RMOs indicated that the simulations provide a useful tool for learning OHNS-related anatomy and as an introduction to ENT-speciﬁc procedures (Otorhinolaryngology - Head & Neck Surgery (OHNS)). |
| 5 | Ferracani, et al. (2015). Natural and virtual environments for the training of emergency medicine personnel. *Universal Access in the Information Society*, *14*(3), 351-362. | VR  Cognitive/affective skills | The possibility to replicate very complex scenarios at a very low cost. | Emergency Medicine  Specialists (continuous education)  Ten evaluators (6 researchers and 4 medical operators in the ﬁeld of emergency medicine) | Quantitative  Heuristics evaluation | The results show that the users were highly engaged in the virtual reality experience, even if there are still some issues related to gesture understanding and tasks accomplishment due probably to the prototypal state of the system. |
| 6 | Ferrer-Torregrosa, J., et al. (2015). ARBOOK: Development and assessment of a tool based on augmented reality for anatomy. *Journal of Science Education and Technology*, *24*(1), 119-124. | AR  Cognitive/Affective skills | To encourage motivation and self-directed learning | First year bachelor in Medical & Biomedical & Health  Anatomy  A total of 211 students from 7 public and private Spanish universities | Quantitative  Control group received standard teaching sessions supported by books, and video. The ARBOOK group received the same standard sessions but additionally used the ARBOOK tool. | At the end of the training, a written test on lower limb anatomy was done by students. Statistically significant better scorings for the ARBOOK group were found on attention–motivation, autonomous work and three-dimensional comprehension tasks. Additionally, significantly better scoring was obtained by the ARBOOK group in the written test. Concretely, the results indicate how this technology is helpful for student motivation, autonomous work or spatial interpretation. |
| 7 | Freschi, C., et al. (2015). Hybrid simulation using mixed reality for interventional ultrasound imaging training. *International journal of computer assisted radiology and surgery*, *10*(7), 1109-1115. | MR  Cognitive/Psychomotor skills | Provide safe practice opportunities for novices, without putting stress on patients. | Medicine  Hand–eye coordination training in diagnostic and interventional ultrasound imaging  Mixed: doctor, resident, medical student, healthcare assistant and non-medical staff (36 novice and 4 experts) | Quantitative  The performances of the hybrid (HG) versus physical (PG) simulator were compared with novices and experts. | Success hybrid approach (78 %) and physical phantom (45 %): Fisher’s exact test was significant: p value is 0.04 hybrid system has been judged easy to use, good for providing adequate information for the 3D perception skill acquisition and very useful for training novices, phantom realism received the lowest score. |
| 8 | Fucentese, S. F., et al. (2015). Evaluation of a virtual-reality-based simulator using passive haptic feedback for knee arthroscopy. *Knee Surgery, Sports Traumatology, Arthroscopy*, *23*(4), 1077-1085. | VR  Cognitive/Psychomotor skills | In current practice, mainly plastic and cadaver models are employed to teach adequate skills to residents. Plastic models offer only reduced realism, while using cadavers implies high maintenance requirements and costs. VR simulators are explored as a possible alternative, also for arthroscopy simulators. | Medicine  Knee arthroscopic (KA) surgery  Specialization training (Residence); Specialists (continuous education)  Participants [N=68: novices (N=33, 20 KA), intermediates (N=19, 21–99 KA), and experts (N=16, 100 KA)]. | Quantitative  Face validity: questionnaire with Likert scales and construct validity: simulator metrics | Face validity was rated with a mean value of 5.5 points (1-7 point scale). Global training capacity scored a mean value of 5.9. Participants considered the simulator as useful for procedural training of diagnostic and therapeutic arthroscopy. The simulator achieved high scores in terms of realism. It was regarded as a useful training tool. |
| 9 | Gomez, P. P., et al. (2015). Development of a virtual reality robotic surgical curriculum using the da Vinci Si surgical system. *Surgical endoscopy*, *29*(8), 2171-2179. | VR  Cognitive/Psychomotor skills | This integrated high fidelity VR simulator has the ability to function without the need of the robotic arms, allowing surgeons from any specialty to improve their robotic skills and adjust to the master surgeon’s console controls in a safe, controlled environment. | Medicine  laparoscopic surgery  Specialization training (Residence); Specialists (continuous education)  22 participants (14 junior, four senior residents and four faculty). | Quantitative | A significant improvement in the seven core robotic skills was observed from pre- to post-test regardless of participant’s level of training (two-tailed paired t test, p < .001). Interestingly, participants were only able to achieve an overall score of 80 % or higher in only five of the seven exercises during post-test.  When overall performance was compared among groups, no difference was found independent of the level of training or surgical expertise (ANCOVA, all p > .05). |
| 10 | Grover, S. C., et al. (2015). Impact of a simulation-training curriculum on technical and nontechnical skills in colonoscopy: a randomized trial. *Gastrointestinal endoscopy*, *82*(6), 1072-1079. | VR  Cognitive/Psychomotor/Affective skills | The study does not have a direct rationale for VR. It compares two designs both with VR. | Medicine  Technical and nontechnical skills in colonoscopy  Specialization training (Residence)  N=33: SCC=16 SRL=17 | Quantitative  33 novice endoscopists; 2 groups; to a structured comprehensive curriculum (SCC) group or self-regulated learning (SRL) group. | No signiﬁcant difference in baseline or post-training performance on the simulator task. The SCC group performed superiorly during their ﬁrst and second clinical colonoscopies. Additionally, the SCC group demonstrated signiﬁcantly better knowledge and colonoscopy-speciﬁc performance, communication, and global performance during the integrated scenario. |
| 11 | Hashimoto, D. A., et al. (2015). Deliberate practice enhances quality of laparoscopic surgical performance in a randomized controlled trial: from arrested development to expert performance. *Surgical endoscopy*, *29*(11), 3154-3162 | VR  Psychomotor skills | Deliberate practice enhances quality of laparoscopic surgical performance | Medicine  laparoscopic surgery  Specialization training (Residence)  Fourteen residents were randomized into deliberate practice (n=7) or control training (n=7). | Quantitative  Randomized - Control Trial (RCT) | Both groups improved over 20 VR LCs in time, dexterity, and global rating scales (all p < 0.05). After 20 LCs, there were no differences in speed or dexterity between groups. The DP group achieved higher quality of VR surgical performance than control for GRS (26 vs. 20, p = 0.001) and PSRS (18 vs. 15, p = 0.001). |
| 12 | Holloway, T., et al. (2015). Operator experience determines performance in a simulated computer-based brain tumor resection task. *International journal of computer assisted radiology and surgery*, *10*(11), 1853-1862. | VR  Psychomotor skills | The consequences of errors during brain surgery can be severe. Using surgical simulators, surgeons may practice in VR. This could theoretically reduce procedural errors, especially during training. | Medicine  Neurosurgical skills  Medical students and neurosurgery residents  Medical students (n=71) and neurosurgery residents (n=12) | Quantitative | In sum, neurosurgery residents removed more tumor, removed less healthy brain, and required less instrument movement than medical students. Coaching modiﬁed medical student performance. |
| 13 | Huang, C., et al. (2015). Face and content validity of a virtual-reality simulator for myringotomy with tube placement. *Journal of Otolaryngology-Head & Neck Surgery*, *44*(1), 40. | MR  Psychomotor skills | Myringotomy with tube insertion in Otolaryngology—Head and Neck Surgery 🡪 The instruction of junior trainees is often challenging. The goal of simulation is to decrease the learning curve prior to entering the operating, minimize complications in patients, and provide the ability to practice difficult cases. | Medicine  Otolaryngology — Head and Neck Surgery  Specialization training (Residence); Specialists (continuous education)  7 junior Otolaryngology (postgrad year 1-3) and 5 specialist Otolaryngology (over 200 procedures) | Quantitative  A questionnaire was developed and used (n=12) to assess face (14 q's) and content validity (6 q's) of a VR-simulator | Responses to 12 of the 14 questions on face validity were predominantly positive. With regard to content validity, 64 % of the responses were positive, 21 % were neutral, and 15 % were negative. |
| 14 | Hudson, K., et al. (2015). Second Life simulation as a strategy to enhance decision‐making in diabetes care: a case study. *Journal of clinical nursing*, *24*(5-6), 797-804. | VR Second Life  Cognitive skills | Nurses can practice clinical decision-making – especially in difficult scenarios/situations. | 12 registered nurses  Diabetes care  Specialists (continuous education) | Quantitative | Nurses with more years of practice reported difﬁculty in using Second Life. As age increased, the total Situational Awareness Score decreased. Day shift nurses were more likely to obtain a High Situational Awareness Score. |
| 15 | Jacobsen, M. E., et al. (2015). Testing basic competency in knee arthroscopy using a virtual reality simulator: exploring validity and reliability. *JBJS*, *97*(9), 775-781. | VR  Psychomotor skills | In traditional model of surgical training, inexperienced trainees have a higher risk of complications, such as damage to chondral surfaces, early in the learning curve. | Medicine  Orthopaedic surgery  Specialization training (Residence)  Twenty-six physicians (thirteen novices and thirteen experienced) | Quantitative  Data from the tests were extracted from the simulator metrics. Pass/fail, and the consequences were explored. | The ﬁnal test showed an intercase reliability of 0.87. The pass-or-fail standard was set at a total z-score of 15.5 points, resulting in two of the novices passing the test and a single experienced surgeon failing the test. |
| 16 | Levac, D., et al. (2015). “Kinect-ing” with clinicians: A knowledge translation resource to support decision making about video game use in rehabilitation. *Physical therapy*, *95*(3), 426-440. | VR  Psychomotor skills | To support clinical decision making about selection and use of Kinect games in physical therapy. The purpose is to build a repository that helps participants become self-paced, self-directed learners with up-to-date multimedia content. | Medicine - Outpatient neurorehabilitation  Physical therapy  Specialists (continuous education) | Qualitative  Four usability studies conducted as either focus groups or formative evaluations with subject matter experts. | Comments regarding categories and information that could be added were numerous and specific. Clinicians requested ranking information for the games in the different categories, as well as including a decision-making algorithm in the online resource to facilitate finding the games that might best fit a particular goal or client population. |
| 17 | Suzuki, T., et al. (2015). An evaluation of the endoscopic surgical skills assessment using a video analysis software program. *Surgical endoscopy*, *29*(7), 1804-1808. | VR  Cognitive/Psychomotor skills | To assess surgeons’ endoscopic surgical skills.  The results of this study demonstrated that the results of the motion analysis by the Dartfish Software were well correlated with the surgeon’s skill level. | Medicine  Endoscopic surgery  Specialization training (Residence); Specialists (continuous education)  6 experts (more than 100 laparoscopic surgeries) and 11 novices (no laparoscopic surgery) | Quantitative  Experimental - Comparison (control) | The Wilcoxon signed-ranks test for related data was used to assess the differences in performance for all of the parameters measured for each group. The Mann–Whitney U-test was applied for comparisons among each of the training groups. There were statistically significant differences between the experts and novices in all three variables assessed (task time: p = 0.0011, the locus tracing of the left sides’ needle holders: p = 0.0011, the locus tracing of the right sides’ needle holders: p = 0.0011). |
|  |  |  |  |  |  |  |

* Usability study - Testing if the study/tool is aligned with the intended teaching/learning purpose.

Table 2. Overview of the use of AR/VR/MR in research in 2016 and their methodological quality to train students and professionals in medicine, biomedical and health sciences (n=24).

|  | **Article** | **AR/VR/MR**  **Learning & behavioral outcomes (Bloom’s taxonomy)** | **What is the rationale for AR/VR/MR exposure** | **In which domain of healthcare are AR/VR/MR being used? Who are the participants?** | **What type of design/methodology is used in these studies?** | **What are the findings?** |
| --- | --- | --- | --- | --- | --- | --- |
|  |  |  |  |  |  |  |
| 18 | Allen, L. K., et al. (2016). Evaluation of an online three‐dimensional interactive resource for undergraduate neuroanatomy education. *Anatomical sciences education*, *9*(5), 431-439. | VR -3D learning  Cognitive skills | This study seeks to contribute to the growing body of research surrounding the construction and application of novel 3D learning resources. | Medicine - 47 second year Bachelor - Neuroanatomy | Quantitative  crossover design divided the participants into two groups | Students who initially accessed the 3D online resources scored significantly better on the Quiz 1 than students who accessed the gross anatomy resources. Scores significantly improved on Quiz 2 for participants who accessed the 3D learning module following exposure to the cadaveric resources. After exposure to both learning modalities, there were no significant differences between groups. Significant positive correlations were found between participants’ spatial ability score and their performance on the Pretest, Quiz 1, and Quiz 2 assessments. |
| 19 | Diment, L. E., et al. (2016). Comparing surgical experience with performance on a sinus surgery simulator. *ANZ journal of surgery*, *86*(12), 990-995. | VR  Cognitive/Psychomotor skills | Simulators are used as important tools for facilitating improved technical proficiency and reducing surgical error. | Medicine  sinus surgery  Specialization training (Residence);Specialists (continuous education) | Quantitative  Experimental - Comparison (control)  Novices (7 master and 7 residence) vs experienced sinus surgeons (10) on 3 tasks | The study demonstrated the construct validity differentiating experts and novices on time, distance travelled and number of cutting motions. Indicating that the simulator could be used for training. |
| 20 | Dorozhkin, D., et al. (2016). Face and content validation of a Virtual Translumenal Endoscopic Surgery Trainer (VTEST™). *Surgical endoscopy*, *30*(12), 5529-5536. | VR  Cognitive skills | One of the identified problems is the urgent need for effective training platforms - assessing the face and content validity of the latest VTESTTM version. | Medicine  Endoscopic Surgery  Specialists (continuous education) | Quantitative  Usability study *  6 of the 12 participants are experts with endoscopic surgery | Participants rated 60% of items questions as 3.0 or greater, for face validity questions regarding the realism of the anatomical features, interface, and the tasks. Content validity results indicate a high level of usefulness of the VTESTTM for training prior to operating room experience. |
| 21 | Ferrer-Torregrosa, J., et al. (2016). Distance learning ects and flipped classroom in the anatomy learning: comparative study of the use of augmented reality, video and notes. BMC medical education, 16(1), 230. | AR  Cognitive skills | Students are used to handling technologies like Internet, 3D video games, mobile phones, MP3 players, etc. We need to change didactic methods, and particularly didactic aids, in order to encourage students to use the abilities and intelligence they usually develop for studying. | Medical  Anatomy  Bachelor  N=171 (78 Medicine, 48 Physiotherapy, and 45 Podiatry) | Quantitative  Quasi-experimental Transversal comparison of equivalent groups design. | The questionnaire assessed the acquired knowledge through a course exam, where 5.60 points were obtained for the notes group, 6.54 for the video group, and 7.19 for the augmented reality group. That is 0.94 more points for the video group compared with the notes and 1.59 more points for the augmented reality group compared with the notes group. |
| 22 | Fischer, M., et al. (2016). Preclinical usability study of multiple augmented reality concepts for K-wire placement. *International journal of computer assisted radiology and surgery*, *11*(6), 1007-1014. | AR  Cognitive skills | Systems with augmented video may benefit of the use of RGBD cameras, which allows the positioning of the virtual cameras and renderings of the patient surface from arbitrary perspectives. RGBD information can also be used to improve the understanding of the environment and enhance the augmentation. | Medicine  orthopedic surgeries  Specialists (continuous education)  7 trained surgeons | Quantitative  Usability study  Using three different mixed reality visualization systems to perform K-wire placement into the superior pubic ramus | Mixed reality systems have great potential to significantly increase surgical efficiency. |
| 23 | Hu, A., et al. (2016). Motivation in computer‐assisted instruction. *The Laryngoscope*, *126*, S5-S13. | VR -3D computer module  Cognitive/Affective skills | Computer-assisted instruction is motivating for students in today’s fast-paced and electronic world. | Medicine  Learning anatomy with the 3D computer module  Randomly chosen participants from bachelor of medicine | Quantitative  Randomized - Control Trial (RCT)  Medical students learning anatomy with the 3D computer vs learning from the written text. | This study concluded that both methods were effective at teaching anatomy for the long term. |
| 24 | Küçük, S., et al. (2016). Learning anatomy via mobile augmented reality: effects on achievement and cognitive load. *Anatomical sciences education*, *9*(5), 411-421. | AR  Cognitive skills | Can facilitate meaningful learning and instant feedback, and an immersive, attractive learning experience which can improve student satisfaction, help students structure their knowledge, and enhance their academic performance. | Medicine  Learning anatomy via mobile augmented reality  Bachelor  70 students second year bachelor medicine | Mixed  RCT  A true experimental, concurrent nested mixed method design | The experimental group students who learned anatomy via mAR applications were more successful than the control group when studying anatomy. |
| 25 | Lin, D., et al. (2016). What are the demographic predictors in laparoscopic simulator performance? *ANZ journal of surgery*, *86*(12), 983-989. | VR  Cognitive skills | Simulators provide a way for educators to develop competencies prior to transitioning into the operating room. | Medicine  Laparascopic surgery  Final year medical students, interns, registered medical ofﬁcers, surgical trainees and surgeons.  370 were randomized RCT | Quantitative  Randomized - Control Trial (RCT)  Two groups: A fundamentals of laparoscopic surgery (FLS) or a LapSim (Surgical Science, Goteborg, Sweden) simulator. | Skills acquisition on both simulators was positively affected by surgical experience. Gender was an inﬂuential factor on the LapSim with men reaching proﬁciency sooner than women. The effect of gaming had no clear inﬂuence on the participants’ scores; and playing a musical instrument had no impact. Practicing non-surgical tasks requiring manual dexterity and handedness were not an inﬂuential factor in total proﬁcient scores, but had a signiﬁcant impact on individual task scores on the FLS simulator. |
| 26 | Ma, M., et al. (2016). Personalized augmented reality for anatomy education. *Clinical Anatomy*, *29*(4), 446-453. | AR  Cognitive skills | Considering the benefits of the personalized and interactive AR system for motivation and perception of anatomy learning. | 72 participants from Bachelor Medicine  Anatomy | Quantitative  Usability study  AR magic mirror and has primarily been developed for medical anatomy education | Results from the user studies demonstrated that the magic mirror system is precise enough for the students to learn anatomy. The AR view of the user body is great and helps the students link the knowledge to the real human body. |
| 27 | Mathiowetz, V., et al. (2016). Comparison of a gross anatomy laboratory to online anatomy software for teaching anatomy. *Anatomical sciences education*, *9*(1), 52-59. | VR  Cognitive skills | The goal was to determine if equivalent learning outcomes could be achieved regardless of the learning tool used (laboratory are considered too costly). | Anatomy for occupational therapy students (HBO)  Masters (Interns)  85 first-year, second-semester: 32 chose laboratory, 53 AnatomyTV | Mixed  Experimental - Comparison (control)  Quasi experimental: two groups of anatomy students, perceived learning, and satisfaction. Mainly quantitative with in addition focus groups | Anatomy laboratory group did significantly better than the online AnatomyTV group in course grade percentages, self-perceived learning, and satisfaction. |
| 28 | Medellín-Castillo, H. I., et al. (2016). The evaluation of a novel haptic-enabled virtual reality approach for computer-aided cephalometry. *Computer methods and programs in biomedicine*, *130*, 46-53. | VR  Cognitive/Psychomotor skills | In oral and maxillofacial surgery, the haptic-enabled 3D digital cephalometric approach reduced errors, and task completion time. | 21 dental surgeons  Specialists (continuous education) | Quantitative  Case studies  21 dental surgeons (comprising 7 Novices, 7 Semi-experts and 7 Experts) performed a range of case studies using a haptic-enabled 2D, 2½D and 3D digital cephalometric analyses. | The results revealed that 3D cephalometry significantly reduced landmarking errors and variability compared to 2D methods. |
| 29 | Middleton, R. M., et al. (2016). Which global rating scale? A comparison of the ASSET, BAKSSS, and IGARS for the assessment of simulated arthroscopic skills. *JBJS*, *98*(1), 75-81. | VR  Cognitive/Psychomotor skills | To address the transition from apprenticeship-based training to competency-based training, methods for surgical skill. | Medicine  Orthopaedics surgery  63 participants: 31 novices, 21 had fewer than 100 independent arthroscopic surgeries, 11 experts with more than 100 independent arthroscopic procedures | Quantitative  Experimental - Comparison (control) | The Kruskal-Wallis test demonstrated signiﬁcant differences between groups for the ASSET, BAKSSS, and IGARS. Subgroup analysis showed that these differences were present for novices compared with trainees and for trainees compared with experts, demonstrating construct validity for all of the global rating scales.  No single global rating scale demonstrated superiority as an assessment tool. |
| 30 | Miki, T., et al. (2016). Development of a virtual reality training system for endoscope-assisted submandibular gland removal. *Journal of Cranio-Maxillofacial Surgery*, *44*(11), 1800-1805. | VR  Psychomotor skills | Technical training prior to the performance of surgery for novice surgeons and trainees. | Medicine  endoscope-assisted submandibular gland removal surgery  Specialization training (Residence)  n=14 (7/7) expert/novice surgeons | Quantitative  Randomized - Control Trial (RCT)  RCT groups with and without training were compared | Demonstrated efficacy in shortening surgery durations (p< 0.05) , decreasing the number of Pean forcep strokes required (p<0.01), and improving the performance of endoscope-assisted surgery. |
| 31 | Miller, M. (2016). Use of computer‐aided holographic models improves performance in a cadaver dissection‐based course in gross anatomy. *Clinical Anatomy*, *29*(7), 917-924. | VR- 3D computer software  Cognitive skills | Teaching gross anatomy with non-traditional approaches including manipulable computer-aided 3-dimensionalmodels, can improve exam performance, and presumably mastery of anatomical knowledge. | Medicine  Anatomy – Osteopathy  Specialization training (Residence)  130–135 students; 56% were men and 44% were women | Quantitative  Usability study  Gross anatomy using the dissection of donor cadavers, manipulation of digitized 3-dimensional holo-graphic renderings, and examination of plastinated specimens. | The use of holographic models apparently reaches students who may be challenged to learn the material using traditional approaches. This may be linked to potentially predictive information gleaned through performance on the MCAT. |
| 32 | Mueller, C. L., et al. (2016). Validity evidence for a new portable, lower-cost platform for the fundamentals of endoscopic surgery skills test. *Surgical endoscopy*, *30*(3), 1107-1112. | VR  Cognitive/Psychomotor skills | To provide an objective assessment of the knowledge and skills required to perform basic endoscopy, the Society of American Gastrointestinal and Endoscopic Surgeons (SAGES) developed the fundamentals of endoscopic surgery (FES^TM^) programme. FES^TM^ includes a didactic educational component, a knowledge test and a hands-on skills assessment on a virtual reality simulator. The study aims to assess equivalence of scores obtained on two platforms, so they can be used interchangeably for testing. | Medicine  Endoscopic surgery  Masters (Interns); Specialists (continuous education)  58 General surgery residents at various levels of training and practicing endoscopists | Quantitative  A multi-institution, randomized, prospective study | There is a high correlation between FES manual skills scores measured on the original platform and the new Express, providing evidence to support the use of the GI MentorTM Express for FES testing. |
| 33 | Nickel, F., et al. (2016). Successful learning of surgical liver anatomy in a computer-based teaching module. *International journal of computer assisted radiology and surgery*, *11*(12), 2295-2301. | VR 3D computer model  Cognitive skills | A comparison study showing 3D was superior to 2D for learning of surgical liver anatomy. | Medicine  Anatomy  Medical Bachelor and Masters (Interns) in their third to fifth year of training (N=410) | Quantitative  Randomized - Control Trial (RCT)  comparing two- (2D) and three-dimensional (3D) presentation modes in a TM for surgical liver anatomy | 3D was superior to 2D for learning of surgical liver anatomy. With training 2D showed similar results. Fun and gender were relevant factors for learning success. |
| 34 | Pan, X., et al. (2016). The responses of medical general practitioners to unreasonable patient demand for antibiotics-a study of medical ethics using immersive virtual reality. *PloS one*, *11*(2), e0146837. | VR  Cognitive skills | VR is useful for situations where ecologically valid studies with human participants cannot be conducted for ethical or practical reasons. | Medicine ethics  21 GP Trainees and GPs  Specialization training (Residence); Specialists (continuous education) | Mixed  Usability study | Experienced GPs are more likely to withstand the pressure to prescribe antibiotics than trainee doctors, thus answering our first question positively. Overall participants did tend towards the illusion of being in the consultation room depicted in the virtual reality and that the virtual consultation taking place was really happening. |
| 35 | Peterson, D. C., et al. (2016). Analysis of traditional versus three‐dimensional augmented curriculum on anatomical learning outcome measures. *Anatomical sciences education*, *9*(6), 529-536. | VR  Cognitive/Psychomotor skills | An integration of traditional cadaveric dissection paired with new computerized technologies was likely to provide the best learning outcomes | Biomedical  Anatomy and cadaveric dissection  Masters (Interns)  55 graduate and 5 upper level undergraduate students (VR group) | Quantitative  Experimental - Comparison (control)  Augmented curriculum versus traditional curriculum | The study showed that the addition of these digital 3D teaching tools significantly improved student understanding and performance on the laboratory examinations. |
| 36 | Rahm, S., et al. (2016). Validation of a virtual reality-based simulator for shoulder arthroscopy. *Knee Surgery, Sports Traumatology, Arthroscopy*, *24*(5), 1730-1737. | VR  Cognitive/Psychomotor skills | First steps to incorporate this VR training into the daily training of residents as a safe method to teach and learn the rather difficult shoulder arthroscopy skills before performing real-life arthroscopy. | Medicine  Orthopaedic surgery  Masters (Interns); Specialists (continuous) education  Participants (N=51) grouped into novices and experts. | Quantitative  Usability study  Establishing face and construct validity | The main study questions could be answered, and the participants considered the presented two exercises as a realistic representation of a real shoulder arthroscopy. They recommend the tested simulator as a useful tool for orthopaedic residents. |
| 37 | Rasmussen, S. R., et al. (2016). Notes from the field: Secondary task precision for cognitive load estimation during virtual reality surgical simulation training. *Evaluation & the health professions*, *39*(1), 114-120. | VR  Cognitive skills | The CLT framework proposes educational design strategies that can be used to reduce the cognitive load. | 24 Bachelor of Medicine  VR temporal bone surgical simulation training. | Quantitative  Randomized - Control Trial (RCT)  Participants were randomized to receive supplementary simulator-integrated tutoring during the first five sessions (Group 1) or not (Group 2). | Secondary task precision was found to be significantly lower during simulation compared with nonsimulation baseline, p < .001. Contrary to expectations, simulator-integrated tutoring and repeated practice did not have an impact on secondary task precision. |
| 38 | Sankaranarayanan, G., et al. (2016). Face validation of the virtual electrosurgery skill trainer (VEST©). *Surgical endoscopy*, *30*(2), 730-738. | VR -3D virtual reality  Cognitive/Psychomotor skills | Though there is an established program for training in both cognitive and motor skills for laparoscopic and endoscopic surgery, none exists for the usage of energy-based devices. | Medicine  Electrosurgery  Masters (Interns); Specialists (continuous education)  63 participants (50 males and 13 females). The surgical experience ranged from medical student to attending | Quantitative  Assessing the face validity of the (Virtual Electrosurgery Skill Trainer) VEST simulator | FUSE experience (n = 15) and no FUSE experience (n = 48). Mann–Whitney U test showed no signiﬁcant difference indicating a general agreement. 46 % of the respondents preferred VEST compared with 52 % who preferred animal model and 2 % preferred both for training in electrosurgery. |
| 39 | Teeter, W., et al. (2016). 323 Virtual Reality Simulation Can Help Prepare Emergency Physicians for REBOA. Annals of Emergency Medicine, 68(4), S124-S125. | VR  Cognitive/Psychomotor skills | Improvements in procedural time and knowledge by emergency physicians based on VRS. | Emergency Medicine  Endovascular skills training for REBOA:  Specialization training (Residence)  N=10 (mean Postgraduate level of 4.6 years (SD 0.5). | Quantitative  Pretest-Posttest  Resuscitative endovascular balloon occlusion of the aorta | Significant improvements in procedural time and knowledge (performance metrics: procedural time; accurate placement of guide wire, sheath, and balloon; correct sequence of steps; economy of motion; and safe use of endovascular tools + pre- and post-course test and questionnaire). |
| 40 | Valdis, M., et al. (2016). Evaluation of robotic cardiac surgery simulation training: a randomized controlled trial. *The Journal of thoracic and cardiovascular surgery*, *151*(6), 1498-1505. | VR  Cognitive skills | The wet lab and virtual reality robotic simulation training outperform the current training methods. This study can help guide training programs in investing resources in cost-effective, high-yield simulation exercises. | Medicine  Cardiac Surgery  Specialization training (Residence)  40 surgical trainees each with less than 10 hours of experience with robotic surgical simulator | Quantitative  Randomized - Control Trial (RCT)  Forty surgical trainees were randomized to a wet lab, a dry lab, a virtual reality lab, or a control group | Wet lab trainees showed the greatest improvement in time-based scoring and the objective scoring tool compared with the experts. The average duration of training was shortest for the dry lab and longest for the virtual reality simulation. |
| 41 | Van Nuland, S. E., et al. (2016). The anatomy of E‐Learning tools: Does software usability influence learning outcomes? *Anatomical sciences education*, *9*(4), 378-390. | VR  Cognitive skills | In the anatomical sciences, computer-aided instruction and online learning tools have become a critical component of teaching the intricacies of the human body when physical classroom space and cadaveric resources are limited. | Health sciences  Anatomy  Bachelor  n=70 (3rd and 4th year anatomy) | Quantitative  Pretest-Posttest  Design with alternating use of 2D and 3D environment | Results showed that reaction times and post-test outcomes were similar for both tools. With a very tentative conclusion that a simple 2D e-learning tool might be as effective as more complicated 3D one. |
|  |  |  |  |  |  |  |

* Usability study - Testing if the study/tool is aligned with the intended teaching/learning purpose.

Table 3. Overview of the use of AR/VR/MR in research in 2017 and their methodological quality to train students and professionals in medicine, biomedical and health sciences (n=24).

|  | **Article** | **AR/VR/MR**  **Learning & behavioral outcomes (Bloom’s taxonomy)** | **What is the rationale for AR/VR/MR exposure** | **In which domain of healthcare are AR/VR/MR being used? Who are the participants?** | **What type of design/methodology is used in these studies?** | **What are the findings?** |
| --- | --- | --- | --- | --- | --- | --- |
| 42 |  |  |  |  |  |  |
|  | Agbetoba, A., et al. (2017, February). Educational utility of advanced three‐dimensional virtual imaging in evaluating the anatomical configuration of the frontal recess. In *International forum of allergy & rhinology 7*( 2), 143-148. | VR  Cognitive skills | Conventional learning and presurgical planning has traditionally involved review of 2-dimensional (2D) computed tomography (CT) images in a triplanar format. For the novice trainee, this can create a challenge in thoroughly comprehending the 3-dimensional (3D) spatial orientation of the frontal recess and frontal sinus drainage pathway during endoscopic sinus surgery. | Medicine  Anatomy  45 otorhinolaryngology trainees and 20 medical school students from 5 academic institutions | Quantitative  Randomized - Control Trial (RCT)  2 groups - with one half learning with the 2D method ﬁrst and the other half learning with the 3D method ﬁrst. | Most trainees (89%) believed that the virtual 3D planning software signiﬁcantly improved their understanding of the spatial orientation of the frontal sinus drainage pathway. |
| 43 | Alsalamah, A., et al. (2017). Face and content validity of the virtual reality simulator ‘ScanTrainer®’. *Gynecological surgery*, *14*(1), 18. | VR  Cognitive skills | A simulated performance should appear realistic. | Medicine  Gynecological  Specialization training (Residence)  36 participants: 25 novices and 11 experts | Quantitative  To determine the face and content validity of a virtual reality simulator | Median scores of face validity statements between experts and non-experts using a 10-point visual analogue scale (VAS) ratings ranged between 7.5 and 9.0 (p> 0.05) indicated a high level of agreement. Experts’ median scores of content validity statements ranged from 8.4 to 9.0. |
| 44 | Al‐Saud, L. M., et al. (2017). Drilling into the functional significance of stereopsis: the impact of stereoscopic information on surgical performance. *Ophthalmic and Physiological Optics*, *37*(4), 498-506. | VR  Cognitive skills | One suggested advantage of human binocular vision is the facilitation of sophisticated motor control behaviours via stereopsis – but little empirical evidence exists to support this suggestion. We examined the functional signiﬁcance of stereopsis by exploring whether stereopsis is used to perform a highly skilled real-world motor task essential for the occupational practice of dentistry. | 13 Dentists  Specialists (continuous education) | Quantitative  A cross sectional quantitative study with a repeated measure design. Four different dental tasks under non-stereoscopic and stereoscopic vision conditions, with two levels of task complexity (direct and indirect observation) using a virtual reality dental simulator. | Depth related errors were signiﬁcantly higher under non-stereoscopic viewing but lateral errors did not differ between conditions. Indirect observation led to participants drilling less of the target area compared to direct viewing, but this did not interact with the stereopsis manipulation. |
| 45 | Al‐Saud, L. M., et al. (2017). Feedback and motor skill acquisition using a haptic dental simulator. *European Journal of Dental Education*, *21*(4), 240-247. | VR  Psychomotor skills | Virtual reality (VR) simulation technologies offer an opportunity to present online continuous feedback on surgical performance through presentation of visual and auditory information. | Dentistry  Bachelor  63 participants - with no previous dental training randomly allocated to three groups (n=21 each). | Quantitative  Randomized - Control Trial (RCT)  Completed four tasks during which feedback was given. Skill retention was examined immediately after training, at 1 week and at 1 month post-test. | Groups – 1. device-only feedback: visual display of simulator; 2. verbal feedback: a qualified dental instructor; 3. a combination of instructor and device feedback.  Participants who received instructor-led and VR feedback adopted a more cautious strategy i.e. produced fewer errors and also removed less of the target. In line with multimodal feedback is more effective than unimodal feedback. Group who received feedback from the device alone was the lowest performing. |
| 46 | Bourdel, N., et al. (2017). Augmented reality in gynecologic surgery: evaluation of potential benefits for myomectomy in an experimental uterine model. *Surgical endoscopy*, *31*(1), 456-461. | AR  Cognitive skills | Myomas are not always easy to correctly localize when they do not signiﬁcantly change the surface of the uterus, or are in multiple locations. AR works by overlaying information from another modality, such as MRI and fusing it in real time with the endoscopic images. AR has never been attempted on a very mobile organ like the uterus and has never been developed for gynecology. | Medicine surgery  Uterine incision  Specialization training (Residence)  Ten residents trained in laparoscopy | Quantitative  Experimental - Comparison (control)  To study the accuracy of myoma localization using a new AR system compared to MRI-only localization. | The mean accuracy in the control group was 16.80 mm versus 0.64 mm with AR. In the control group, the mean time to perform the task was 18.68 s compared to 19.6 s with AR. The mean score of difﬁculty (evaluated for each myoma) was 2.36 [1–4] versus 0.87 [0–4], respectively, for the control and the AR group.  We developed an AR system for a very mobile organ. This is the ﬁrst user study to quantitatively evaluate an AR system for improving a surgical task. In our model, AR improves localization accuracy. |
| 47 | Cui, D., et al. (2017). Evaluation of the effectiveness of 3D vascular stereoscopic models in anatomy instruction for first year medical students. *Anatomical sciences education*, *10*(1), 34-45. | VR  Cognitive skills | Efficiency; replacement of laboratory time. | Medicine  anatomy head and neck region  Bachelor - first year n=39 (out of 149 first year): 2D n=18 versus 3D n=21 | Quantitative  Randomized - Control Trial (RCT)  Anatomy knowledge tests (pre-learning session knowledge test and post-learning session knowledge test), Mental Rotation Tests (spatial ability; pre-session MRT and post-session MRT), and a satisfaction survey. | Stereoscopic 3D vascular models in 3D learning sessions increased the ability to correctly identify the head and neck vascular anatomy. Students with low spatial ability improved post session knowledge scores to a level comparable to that demonstrated by students with high-spatial ability. |
| 48 | De La Garza, J. R., et al. (2017). Does rating the operation videos with a checklist score improve the effect of E-learning for bariatric surgical training? Study protocol for a randomized controlled trial. *Trials*, *18*(1), 134. | VR  cognitive  please note: it is study protocol | We hypothesize that using the BOSATS checklist during E-learning will improve the learning curve and facilitate transfer to practice. | Medicine  (Bariatric) surgery  Masters (Interns)  The trainees are medical students from the University of Heidelberg in their clinical years with no prior laparoscopic experience; n= 80 | Quantitative  Randomized - Control Trial (RCT)  The present study aims to explore whether trainees will have an improved learning curve for RYGB on the VR trainer by E-learning and rating videos with a modified BOSATS checklist than just by E-learning without the use of a checklist. | No findings - it is a study protocol for a randomized controlled trial. |
| 49 | Dubovi, I., et al. (2017). Now I know how! The learning process of medication administration among nursing students with non-immersive desktop virtual reality simulation. *Computers & Education*, *113*, 16-27. | VR  Cognitive skills | Computerized virtual reality simulations have the potential to configure a variety of scenarios to determine likely staff responses and how to address them without intensive utilization of resources. | Nursing education  Bachelor  Learning medication administration processes with a PILL-VR simulation platform (experimental group; n=82) or with lecture-based curriculum (n=47; comparison group). | Quantitative  Experimental - Comparison (control)  A quasi-experimental pretest-intervention-posttest comparison group design was conducted based on quantitative analysis of questionnaires, video recordings and worksheets. | The results revealed significantly higher conceptual and procedural knowledge learning gains following activity with the PILL-VR simulation compared to studying via lecture-based curriculum. |
| 50 | Dubovsky, S. L., et al. (2017). A preliminary study of a novel emergency department nursing triage simulation for research applications. *BMC research notes*, *10*(1), 15. | VR  Cognitive skills | Because performance on this (or any other) simulation has not been compared with the actual situations it represents, it was necessary to demonstrate that it could be used as a valid model of an important component of ED activity before we could investigate the effect of varying parameters that impact it. | Specialists (continuous education)  Nurses  Ten experienced female ED triage nurses (mean age 51) mastered navigating a virtual reality model of triage of 4 patients in an ED | Quantitative  Usability study | Nurses perceived their work on the simulation task to be equivalent to their workload on the job in all aspects except for physical exertion. Although they were able to work with written communications with the patients, verbal communication would have been preferable. Consistent with the workplace, variability in performance during triage reflected subject skill and experience and was correlated with comfort with the task. Time to perform triage corresponded to the time required in the ED and virtual patients were prioritized appropriately according to severity. |
| 51 | Huber, T., et al. (2017). New dimensions in surgical training: immersive virtual reality laparoscopic simulation exhilarates surgical staff. *Surgical endoscopy*, *31*(11), 4472-4477. | VR  Cognitive/Psychomotor skills | Training usually takes place outside of the OR. The goal of the current project was to develop a new combined highly IVR laparoscopy setup and to analyze first experiences regarding the degree of immersion, motion sickness, and performance measurements. | Medicine surgery  Specialization training (Residence);Specialists (continuous education)  N=10 members of the surgical department (three females) with varying laparoscopic experience levels (two attending surgeons, two surgical fellows, two PGY-5, two PGY-2, and two medical students). | Quantitative  Usability study  This is the first clinical and technical feasibility study using the full IVR laparoscopy setup combined with the latest laparoscopic simulator in a 360° surrounding. Participants were exhilarated by the high level of immersion. The setup enables a completely new generation of surgical training. | Participants’ times for fine dissection were significantly longer during the IVR session (regular: 86.51 s [62.57 s; 119.62 s] vs. IVR: 112.35 s [82.08 s; 179.40 s]; p = 0.022). The cholecystectomy task had higher error rates during IVR. Motion sickness did not occur at any time for any participant. Participants experienced a high level of exhilaration, rarely thought about others in the room, and had a high impression of presence in the generated IVR world. |
| 52 | Locketz, G. D., et al. (2017). Anatomy-specific virtual reality simulation in temporal bone dissection: perceived utility and impact on surgeon confidence. *Otolaryngology–Head and Neck Surgery*, *156*(6), 1142-1149. | VR  Affective outcomes | The major focus of VR simulation has revolved around resident training, with an emphasis on anatomic understanding, technique development, and familiarization with various approaches. | Medicine  Surgery confidence  Specialization training (Residence)  16 residents were enrolled, 9 men and 7 women, ranging in training level from postgraduate years (PGYs) 2 through 5. | Quantitative  Prospective pre- and poststudy of a novel virtual surgical rehearsal platform. | Of 16 subjects, 14 (87.5%) reported a significant increase in overall confidence after conducting an anatomy-specific VR rehearsal. A significant correlation existed between perceived utility of rehearsal and confidence improvement. The effect of rehearsal on confidence was dependent on trainee experience and the inherent difficulty of the surgical subtask. Post rehearsal confidence correlated strongly with graded dissection performance. Subjects rated anatomy-specific rehearsal as having a moderate to high contribution to their dissection performance. |
| 53 | Mathews, S., et al. (2017). Predictors of laparoscopic simulation performance among practicing obstetrician gynecologists. *American journal of obstetrics and gynecology*, *217*(5), 596-e1. | VR  Cognitive/Psychomotor skills | The goal of the initiative is to develop a method by which to assess, improve, and maintain laparoscopic skill levels for attending gynecologic laparoscopic surgeons, and ultimately develop criteria by which to use simulation in the credentialing and privileging process for gynecologic laparoscopic surgery. | Medicine  Gynecologists  Specialists(continuous education)  357 physicians participated in the quality improvement initiative. The included physicians had an average of 14.4 years in practice (range 0-50 years) and 34.8% were fellowship-trained specialists. | Quantitative  Usability study  All gynecologists with laparoscopic privileges from each institution were required to complete a questionnaire and laparoscopic surgery simulation assessment. | The average number of laparoscopic procedures per month was a significant predictor of total time on all 3 tasks (P = .001 for peg transfer; P = .041 for lifting and grasping; P < .001 for cutting). Average monthly laparoscopic surgical volume was a significant predictor of 2 efficiency scores in peg transfer, and all 4 efficiency scores in cutting (P = .001 to P = .015). Surgical volume was a significant predictor of errors in lifting/grasping and cutting (P < .001 for both). Self-rated laparoscopic skill level was a significant predictor of total time in all 3 tasks (P < .0001 for peg transfer; P = .009 for lifting and grasping; P < .001 for cutting) and a significant predictor of nearly all efficiency scores and errors scores in all 3 tasks. |
| 54 | Moro, C., et al. (2017). The effectiveness of virtual and augmented reality in health sciences and medical anatomy. *Anatomical sciences education*, *10*(6), 549-559. | AR and VR  Cognitive skills | Substitute for use of cadavers given substantial financial, ethical, and supervisory constraints on their use. | Medical and Biomedical and Health  Anatomy  Bachelor  59 participants | Quantitative  Randomized - Control Trial (RCT)  3 conditions: a VR, an AR and a Tablet based application | No significant differences were found between mean assessment scores in VR, AR, or TB. Participants rated their learning experience highly in all seven domains across the three learning modes. |
| 55 | Sampogna, G., et al. (2017). Routine clinical application of virtual reality in abdominal surgery. *Minimally Invasive Therapy & Allied Technologies*, *26*(3), 135-143. | VR  Cognitive skills | Virtual simulation may solve the pressing medico-legal issues and ethical considerations associated with medical education, as it is an eco-sustainable way of learning diagnostic and therapeutic procedures having the intrinsic potential to harm patients (9). Moreover, VR may be used for patient-specific procedure rehearsal, before performing any interventions on patients themselves. | Medicine  abdominal surgery  Specialization training (Residence);Specialists (continuous education)  15 patients undergoing pancreatic, hepatic or renal surgery were studied realizing a 3D reconstruction of target anatomy. | Qualitative  A qualitative evaluation of the three approaches was performed by 20 surgeons, who filled in a specific questionnaire regarding a clinical case for each organ considered. | Preoperative surgical planning and intraoperative guidance was feasible for all patients included in the study. The vast majority of surgeons interviewed scored their quality and usefulness as very good. |
| 56 | Saratzis, A., et al. (2017). Role of simulation in endovascular aneurysm repair (EVAR) training: a preliminary study. *European Journal of Vascular and Endovascular Surgery*, *53*(2), 193-198. | VR  Cognitive/Psychomotor skills | This study confirms that virtual reality-based simulation in endovascular surgery can improve several aspects of trainee performance and contribute to patient safety. It should be strongly considered as part of structured vascular training in future. | Medicine surgery  Endovascular Aneurysm Repair (EVAR) Training  Specialization training (Residence);Specialists (continuous education)  16 vascular surgical trainees, 4 consultant vascular surgeons, and 2 consultant interventional radiologists | Quantitative  Usability study  This study aimed to: a) benchmark competency levels using EVAR SBT, and b) investigate the impact of supervised SBT on trainee performance. | Median procedural-time for consultants was 43.5 min (IQR 7.5). A significant improvement in trainee procedural-time following SBT was observed (median procedural time 77 min [IQR 20.75] vs. 56 min [IQR: 7.00], p < .0001). The mean (SD) trainee Likert score pre- and post-SBT improved (16.6 [SD 1.455] vs. 28.63 [SD 2.986], p < .0001). Fewer endoleaks were observed (p = .0063) and trainees chose an appropriately sized device more often after SBT. |
| 57 | Siebert, J. N., et al. (2017). Adherence to AHA guidelines when adapted for augmented reality glasses for assisted pediatric cardiopulmonary resuscitation: A randomized controlled trial. *Journal of medical Internet research*, *19*(5), e183. | AR  Cognitive skills | AR glasses are wearable and connected devices that display interactive images to the visual field of users by overlaying visual information without significantly disturbing the ordinary vision. Despite recent communications and studies related to the use of these glasses in various medical fields, their contribution to resuscitation in emergency medicine has not yet been investigated. | Medicine  pediatric  Specialization training (Residence)  20 pediatric residents participated and completed the study with no dropout | Quantitative  Randomized - Control Trial (RCT)  2 parallel groups of voluntary pediatric residents, comparing AR glasses to PALS pocket reference cards during a simulation-based pediatric cardiac arrest scenario—pulseless ventricular tachycardia (pVT). | Time to first defibrillation attempt (mean: 146 s) and adherence to AHA guidelines in terms of time to other critical resuscitation endpoints and drug dose delivery were not improved using AR glasses. However, errors and deviations were significantly reduced in terms of defibrillation doses when compared with the use of the PALS pocket reference cards. |
| 58 | Sirimanna, P., & Gladman, M. A. (2017). Development of a proficiency‐based virtual reality simulation training curriculum for laparoscopic appendicectomy. *ANZ journal of surgery*, *87*(10), 760-766. | VR  Cognitive skills | Recently, there has been interest in virtual reality (VR) simulation as this allows technical skill acquisition with improved operating performance and creation of structured training curricula using expert benchmarks of skill, that is, competency-based performance goals. Indeed, such curricula can improve trainee performance in the actual operating theatre. | Medicine  Surgeon - laparoscopic appendicectomy  Specialization training (Residence)  10 experienced (>50 LAs), 8 intermediate (10–30 LAs) and 20 inexperienced (<10 LAs) surgeons | Quantitative  Experimental - Comparison (control)  Surgeons performed guided and unguided LA tasks on a high-ﬁdelity VR simulator using internationally relevant techniques. | 16 (89%) surgeons considered the VR model to be visually realistic and 17 (95%) believed that it was representative of actual practice. All ‘guided’ modules demonstrated construct validity, with learning curves that plateaued between sessions 6 and 9. When comparing inexperienced to intermediates to experienced, the ‘unguided’ LA module demonstrated construct validity for economy of motion and task time. Construct validity was also conﬁrmed for number of movements, path length and idle time. Validated modules were used for curriculum construction, with proﬁciency benchmarks used as performance goals. |
| 59 | Siroen, K. L., et al. (2017). Mastery Learning–does the method of learning make a difference in skills acquisition for robotic surgery?. *The International Journal of Medical Robotics and Computer Assisted Surgery*, *13*(4), e1828. | VR  Cognitive/Psychomotor skills | Performance improves significantly on robotic fundamentals of laparoscopic surgery (FLS) tasks when novice surgeons are able to practice to proficiency on a VR (virtual reality) simulator. | Medicine  robotic surgery - minimally invasive surgery  Participant eligibility criteria included undergraduate students, medical students, and surgery residents. 42 participants were randomized into blocked and random practice groups. | Quantitative  Randomized - Control Trial (RCT)  The dV‐Trainer® and the da Vinci® Surgical System (dVSS) were used to compare practice conditions. Each participant performed five tasks: Ring Walk, Thread the Rings, Needle Targeting, Suture Sponge and Tubes Level 2. Transfer to the dVSS was also assessed. | No significant differences were observed between the two groups, except for a few instances. For example, during Ring Walk, the random group performed significantly faster than the blocked group (100.78 ± 5.26 s vs 121.59 ± 5.26 s, p < 0.01). |
| 60 | Stepan, K., et al. (2017, October). Immersive virtual reality as a teaching tool for neuroanatomy. In *International forum of allergy & rhinology* (Vol. 7, No. 10, pp. 1006-1013). | VR  Cognitive skills | Prohibitive costs and decreased access to cadavers have led to diminished dissection courses. There’s a need to evaluate effectiveness, satisfaction, and motivation associated with immersive VR simulation in teaching medical students neuroanatomy | Medicine  Teaching medical students neuroanatomy  66 students 33 female and 33 male participants; 34 1st year of medical school and 32 participants 2nd year | Quantitative  Randomized - Control Trial (RCT)  anatomy knowledge pre- and posttest, retention quizzes, educational experience, motivation (Instructional Materials Motivation Survey) | No significant difference in anatomy knowledge between the 2 groups (textbook vs VR) on pre-intervention, post-intervention, or retention quizzes. VR group found the learning experience to be significantly more engaging, enjoyable, and useful (all p < 0.01) and scored significantly higher on the motivation assessment (p < 0.01). |
| 61 | Våpenstad, C., et al. (2017). Lack of transfer of skills after virtual reality simulator training with haptic feedback. *Minimally Invasive Therapy & Allied Technologies*, *26*(6), 346-354. | VR  Cognitive/Psychomotor skills | The OR is not the ideal learning environment to train basic technical skills due to the nature of minimally invasive surgery, ethical considerations, working time directives and the increased focus on efficient surgical production. Therefore, new tools that facilitate training outside of the OR are being tested such as VR simulators and box trainers. | Medicine  Laparoscopic Skills  Masters (Interns)  None of the participants had any experience with laparoscopic surgery. | Quantitative  Randomized - Control Trial (RCT)  At initial enrolment, the participants answered a questionnaire with background information. All candidates performed a cholecystectomy on a porcine organ model in a box trainer (the clinical setting). The performances were video rated by two surgeons blinded to subject training status. | In total, 30 students performed the cholecystectomy and had their videos rated (N = 16 simulator group, N = 14 control group). The control group achieved better video rating scores than the simulator group (p < .05).  The criterion-based training program did not transfer skills to the clinical setting. Poor mechanical performance of the simulated haptic feedback is believed to have resulted in a negative training effect. |
| 62 | Wang, S., et al. (2017). Augmented reality as a telemedicine platform for remote procedural training. *Sensors*, *17*(10), 2294. | AR  The use of HoloLens  Cognitive skills | Provision of healthcare to individuals in rural areas represents a significant logistical challenge resulting from geographic, demographic and socioeconomic factors. Recruitment and retention of healthcare providers (HCP) to rural locations continues to be a significant problem. An economical and effective solution to the lack of HCP in rural areas is telemedicine, which uses information technologies to deliver health care services over both large and small distances. | Medicine  Trauma  12 participants with minimal PoCUS experience were enrolled in the pilot study with the HoloLens setup. The other 12 participants were assigned to complete the remote PoCUS training study using a “full telemedicine setup”. | Quantitative  Experimental - Comparison (control)  Performance of the trainee was independently observed and graded by a PoCUS expert using a Global Rating Scale (GRS). | From the expert evaluator’s scores on the GRS for the right upper quadrant exam, there was no signiﬁcant statistical difference between the HoloLens application and the full telemedicine setup. Participants using the HoloLens application took much longer to ﬁnish the procedure than participants completing the full telemedicine setup. The time difference between the two was statistically signiﬁcant. However, trends appeared to suggest that participants felt it was easier to use the HoloLens application to perform an ultrasound scan as the mental effort rating and task difﬁculty rating were lower than the full setup, though there was no signiﬁcant difference between the groups. |
| 63 | Yen, A. J., & Ramanathan, S. (2017). Advanced cataract learning experience in United States ophthalmology residency programs. *Journal of Cataract & Refractive Surgery*, *43*(10), 1350-1355. | VR  cognitive  Comparion of residency programs | Simulation can be a powerful curricular tool in the training of new techniques and devices. In ophthalmology, we know the usefulness of simulation for basic cataract surgery techniques, as well as to improve comfort level prior to working with patients. Simulation can improve technical skills and may improve patient outcomes after cataract surgery. | Medicine surgery  Othalmology  Residency directors - Compare programs  Of the 116 questionnaires sent to residency directors, 71 (61.2%) were completed and included in data analysis. | Quantitative  Comparing residency programs by means of a questionnaire | Program directors reported that 95.3% of their graduating seniors were competent to select and implant toric IOLs and 52.3% were competent to implant capsular tension rings (CTRs). Divide and conquer (56.6%) and stop and chop (25.4%) dominated phacoemulsification teaching. Femtosecond laser– assisted cataract surgery was performed by residents in 44.1% of programs. In 25.4%, residents observed but did not perform the procedure; in 35.6%, they received didactic-only training; and in 22.0%, they received no exposure. |
| 64 | Zupanc, C. M., et al. (2017). Assessing colonoscopic inspection skill using a virtual withdrawal simulation: a preliminary validation of performance metrics. *BMC medical education*, *17*(1), 118. | VR  Cognitive skills | Because of the many factors that may affect performance of the inspection task, it is not obvious how performance can be adequately assessed during live colonoscopy. One alternative is the use of virtual simulation. Simulators offer the possibility of objectively and automatically quantifying many of the factors relevant to effective inspection, and allowing trainees to be assessed on standardized cases. | Medicine  Colonoscopy  Eleven experienced endoscopists and 18 endoscopy novices (medical students) | Quantitative  Experimental - Comparison (control)  The two groups were compared on 10 performance metrics to preliminarily assess the validity of these measures to describe inspection quality. | Statistically significant experienced-novice differences were found for 8 of the 10 performance metrics (p’s < .005). Compared with novices, experienced endoscopists inspected more of the mucosa and detected more polyp markers, at a faster rate. Despite completing the withdrawals more quickly than the novices, the experienced endoscopists also moved the colonoscope more in terms of linear distance travelled and overall tip movement, with greater use of both the up/down angulation control and axial shaft rotation. However, the groups did not differ in the number of polyp markers visible on the monitor but not identified, or movement of the left/right angulation control. |
|  |  |  |  |  |  |  |

Table 4. Overview of the use of AR/VR/MR in research in 2018 and their methodological quality to train students and professionals in medicine, biomedical and health sciences (n=37).

|  | **Title of article** | **AR/VR/MR**  **Learning & behavioral outcomes -Bloom’s taxonomy** | **What is the rationale for AR/VR/MR exposure** | **In which domain of healthcare are AR/VR/MR being used? Who are the participants?** | **What type of design/methodology is used in these studies?** | **What are the findings?** |
| --- | --- | --- | --- | --- | --- | --- |
| 65 |  |  |  |  |  |  |
|  | Ali, S., et al. (2018). Virtual simulation in enhancing procedural training for fluoroscopy-guided lumbar puncture: a pilot study. *Academic radiology*, *25*(2), 235-239. | VR -3D model  Cognitive skills | Fluoroscopy-guided lumbar puncture (FGLP) - Performance of the procedure with limited experience is associated with increased patient discomfort as well as increased radiation dose, puncture attempts, and complication rate. Simulation in health care is a developing field that has potential for enhancing procedural training. We demonstrate the design and utility of a virtual reality simulator for performing FGLP. | Radiology nurse  Specialists (continuous education)  Six participants, five radiology trainees (Post-graduate Year-2, 5, 5, 6, and 7) and one radiology nurse practitioner (with 2 years of non–FGLP related work experience). | Qualitative  Usability study (checking to see if the study/tool aligns with what you want to teach/learn)  They went through a procedure and filled out a survey | On the survey, using a five-point Likert scale, all participants thought that it was “very important” or “extremely important” for the module to realistically replicate anatomy both visually and through haptic feedback. All participants thought the module realistically replicated the anatomy visually through the reconstructed 3-D images and the simulated fluoroscopy, with the responses varying between “somewhat realistic” to “extremely realistic.” Those with prior FGLP experience (5/6) thought the system realistically replicated the anatomy through haptic feedback, with responses ranging from “somewhat realistic” to “realistic.” All participants said “yes” to whether they would recommend the training program to a colleague. On a scale of 1–5 (lowest to highest) rating the virtual simulator training overall, the mean score was 4.3 (range 3–5). |
| 66 | Arya, S., et al. (2018). Role of pelvic ultrasound simulation. *The clinical teacher*, *15*(6), 457-461. | VR  Cognitive skills | Our study aimed to determine the usefulness, applicability and attitudes toward pelvic ultrasound simulation training among residents, sonographers and practising doctors. | Medicine  Pelvic ultrasound simulation  Bachelor; Masters (Interns)  31 participants were involved in a 4‐hour US workshop: 28 (90.3%) participants completed all of the segments of the pelvic US workshop. | Mixed  Pretest-Posttest  All learners completed a pre‐ and post‐encounter quiz, and an anonymous post‐simulation survey on the relevance of ultrasound simulation to clinical learning, and its usefulness to improve scanning performance and interpretation skills. | Pelvic ultrasound simulation activity using high‐fidelity virtual reality ultrasound simulators lasted 4 hours and consisted of three modules: abnormal uterine bleeding, adnexal masses and bleeding in pregnancy. Five respondents agreed and 23 strongly agreed that pelvic ultrasound simulation applies to their clinical ultrasound practice, and seven of them agreed and 21 strongly agreed that their performance of ultrasound and interpretation skills will be improved following their simulation training. The average post‐activity knowledge score for all three topics significantly increased (paired Student's t‐test, p < 0.0001). |
| 67 | Borgersen, N. J., et al. (2018). Virtual reality‐based proficiency test in direct ophthalmoscopy. *Acta ophthalmologica*, *96*(2), e259-e261. | VR  Cognitive skills | Direct ophthalmoscopy is poorly mastered among young physicians and avoided due to lack of proficiency and confidence in the skill. | Medicine  Ophthalmology  Masters (Interns);Specialization training (Residence) | Quantitative  Experimental - Comparison (control) | Relations to other variables showed that the experienced significantly outperformed the novices. Consequences of applying a pass/fail standard showed excellent discriminatory ability with no false positives/negatives. Experienced participants considered the simulator realistic and found that the training programme met the training needs on how to perform direct ophthalmoscopy. |
| 68 | Cecil, J., et al. (2018). An advanced simulator for orthopedic surgical training. *International journal of computer assisted radiology and surgery*, *13*(2), 305-319. | VR  Cognitive skills | The American Board of Orthopedic Surgery (ABOS) has mandated the use of simulation based training in order to improve surgical skills. The developed virtual surgical environment (VSE) deals with training medical residents in an orthopedic surgical process. | Medicine  Orthopedic surgical  Residents/students  37 participants including 24 medical students and 13 residents participated in the activity (20 males and 17 females). | Mixed  Usability study (checking to see if the study/tool aligns with what you want to teach/learn) | Out of 37 residents/students who participated in the test, 32 showed improvements in their understanding of the LISS plating surgical process. A majority of participants were satisﬁed with the use of teaching Avatars and haptic technology. |
| 69 | Chalhoub, M., et al. (2018). The role of smartphone game applications in improving laparoscopic skills. *Advances in medical education and practice*, *9*, 541. | VR  Cognitive/Psychomotor skills | The aim of this study is to show that the positive impact of VG practice on laparoscopic skills can include Smartphone games (SGs), and to find a pattern between the types of SGs played and the type of skills acquired. | Medicine  laparoscopy  Bachelor  A total of 45 medical students with no previous surgical experience were divided into three groups: gamers (n=20), control (n=10) and intervention (n=15). | Quantitative  Experimental - Comparison (control)  Every 5 intervention participants were asked to play a different smartphone application game daily for 2 months between the two sessions. Evolution ratio between sessions was calculated. | Significant advantage was found at session 1 of gamers over non-gamers (p=0.002). No significant difference existed between the two non-gamer groups (p=0.96), or between the three intervention sub-groups (p>0.05). All participants’ performances improved between sessions. No significant difference existed in evolution between control and gamers (p=0.121), nor between intervention and gamers (p=0.189). Significant advantage was found in evolutions of the intervention group over control group (p=0.035). |
| 70 | Cook, et al. (2019). Supporting self-regulation in simulation-based education: a randomized experiment of practice schedules and goals. *Advances in Health Sciences Education*, *24*(2), 199-213. | VR  Cognitive skills | Simulation might be used to support students self-regulated learning. | Medicine  Endoscopy  Bachelor; Masters (Interns);Specialization training (Residence)  Participants (18 surgery interns, 17 research fellows, 5 medical/college students) were randomly assigned to groups. | Quantitative  Usability study  Participants practiced 5 endoscopy tasks on a physical simulator, then completed 4 repetitions on a virtual reality simulator. | Study A compared two practice schedules: sequential versus unstructured. Study B compared normative comparisons framed as success. Study C compared a time-only goal versus time + quality goal. In Study A, the sequential group had higher task completion (10/19 vs. 1/21; P < .001), longer persistence attempting an ultimately incomplete task (20.0 vs. 15.9 min; P = .03), and higher efficiency (43% vs. 27%; P = .02), but task time was similar between groups (20.0 vs. 22.6 min; P = .23). In Study B, the success orientation group had higher task completion (10/16 vs. 1/24; P < .001) and longer persistence (21.2 vs. 14.6 min; P = .001), but efficiency was similar (33% vs. 35%; P = .84). In Study C, the time-only group had greater efficiency than time + quality (56% vs. 41%; P = .03), but task time did not differ significantly (172 vs. 208 s; P = .07). |
| 71 | Courteille, O., et al. (2018). Learning through a virtual patient vs. recorded lecture: a comparison of knowledge retention in a trauma case. *International journal of medical education*, *9*, 86. | VR  Cognitive skills | As reported by Botezatu and colleagues, Virtual Patients (VPs) have proven to engage and motivate trainees, as well as improve learning acquisition and under-standing. It was therefore suggested to use the VP model to offer a valid learning alternative to traditional lecturing in spinal trauma. | Medicine  orthopedic surgery  Bachelor; Specialization training (Residence)  A total of 170 volunteers (85 medical students and 85 residents in orthopedic surgery) | Mixed  Randomized - Control Trial (RCT)  Randomly allocated (to either a video-recorded standard lecture or a Virtual Patient-based training session where they interactively assessed a clinical case portraying a motorcycle accident. | Participants’ learning experiences were evaluated with exit questionnaires. A repeated-measures analysis of variance was applied on knowledge scores. A total of 81% (n = 138) of the participants completed both tests. There was a small but significant decline in first and second test results for both groups (F(1, 135) = 18.154, p = 0.00). However, no significant differences in short-term and long-term knowledge retention were observed between the two teaching methods. The Virtual Patient group reported higher learning experience levels in engagement, stimulation, general perception, and expectations. |
| 72 | Gunn, T., et al. (2018). The use of virtual reality simulation to improve technical skill in the undergraduate medical imaging student. *Interactive Learning Environments*, *26*(5), 613-620. | VR  Cognitive skills | VR software can enable independent practice of medical imaging procedures and skills. | Medicine  General radiography Bachelor -first year  Of the 81 students enrolled in the first year general radiography, 57 students voluntarily completed a formative role play assessment for this study. | Mixed  Usability study  The base model included learning style, anatomy and their interactions on mean role-play score, 95% confidence intervals were reported. Order effects, gaming, gender and age were added one at a time and were removed if not significant. | Data demonstrated an improved total role-play skill score for those students trained using VR software simulation compared with the total role-play skills score traditional laboratory simulation. Demographic multivariable analysis demonstrated no statistically significant association of age, gender, gaming skills/activity with the outcome. The novel medical imaging VR simulation learning tool facilitated technical skill acquisition, equal to, or slightly better than traditional laboratory training. |
| 73 | Hettig, J., et al. (2018). AR in VR: Assessing surgical augmented reality visualizations in a steerable virtual reality environment. *International journal of computer assisted radiology and surgery*, *13*(11), 1717-1725. | AR  Cognitive/Psychomotor skills | A dedicated system architecture was introduced and workﬂow in order to bypass the above-mentioned registration issues and foster the investigation of new visualization applications. | Medicine  Surgery  Specialization training (Residence)  Our study consisted of 11 participants, including 3 senior, 2 consultant and 4 resident surgeons and 2 medical students with surgical experience. | Mixed  Usability study | According to the surgeons, the visual impression of the VR scene is mostly inﬂuenced by 2D surface details and lighting conditions. The AR evaluation shows that, depending on the visualization used and its capability to encode depth, 37% to 91% of the experts made wrong decisions, but were convinced of their correctness. These results show that surgeons have more conﬁdence in their decisions, although they are wrong, when supported by AR visualizations. |
| 74 | Hovgaard, L. H., et al. (2018). Validity evidence for procedural competency in virtual reality robotic simulation, establishing a credible pass/fail standard for the vaginal cuff closure procedure. *Surgical endoscopy*, *32*(10), 4200-4208. | VR  Cognitive/Psychomotor skills | Virtual reality (VR) simulation of robotic surgery can be used to train these new skills and can potentially optimize the early learning curve in robotic surgery. VR simulation enables novice robotic surgeons to practice in a patient-free and safe learning environment allowing also for repeated and distributed practice, which is optimal for learning. Finally, for the experienced surgeon, re-familiarization with the robotic console prior to a procedure as a ‘warm-up’ with the VR simulator improves subsequent performance. | Medicine  Gynaecology  Specialization training (Residence);Specialists (continuous education)  11 robotic surgical novices and 11 experienced surgeons subspecialized in robotic surgery | Quantitative  Usability study  After familiarization with the VR simulator, participants completed the module ‘Guided Vaginal Cuff Closure’ six times. Validity evidence was investigated for 18 preselected simulator metrics. | The experienced surgeons significantly outperformed the novice surgeons on 6 of the 18 metrics. The internal consistency was 0.58 (Cronbach’s alpha). The experienced surgeons’ mean composite score for all six repetitions were significantly better than the novice surgeons’ (76.1 vs. 63.0, respectively, p < 0.001). A pass/fail standard of 75/100 was established. Four novice surgeons passed this standard (false positives) and three experienced surgeons failed (false negatives). |
| 75 | Huang, C. Y., et al. (2018). The use of augmented reality glasses in central line simulation:“see one, simulate many, do one competently, and teach everyone”. *Advances in medical education and practice*, *9*, 357. | AR  Cognitive skills | The aim of this study was to investigate the feasibility of using augmented reality (AR) glasses in central line simulation by novice operators and compare its efficacy to standard central line simulation/teaching. It allows for teaching of complex skills in a controlled environment | Medicine  Surgery  Masters (Interns)  A total of 32 adult novice central line operators with no visual or auditory impairments were enrolled. Medical doctors, respiratory therapists, and sleep technicians were recruited from the medical field. | Quantitative  Randomized - Control Trial (RCT)  This was a prospective randomized controlled study. Subjects were randomized on a 1:1 basis to either simulation using the augmented virtual reality glasses or simulation using conventional instruction. | The mean time for AR placement in the AR group was 71±43 s, and the time to internal jugular (IJ) cannulation was 316±112 s. There was no significant difference in median (minimum, maximum) time (seconds) to IJ cannulation for those who were in the AR group and those who were not (339 [130, 550] vs 287 [35, 475], p=0.09), respectively. There was also no significant difference between the two groups in median total procedure time (524 [329, 792] vs 469 [198, 781], p=0.29), respectively. There was a significant difference in the adherence level between the two groups favoring the AR group (p=0.003). |
| 76 | Huang, Z., et al. (2018). Three-dimensional printing model improves morphological understanding in acetabular fracture learning: A multicenter, randomized, controlled study. *PloS one*, *13*(1), e0191328. | VR  Cognitive skills | Virtual reality (VR) is widely applied in the teaching field and can be used to accurately  reconstruct stereoscopic objects from digital data. The tactile-based Virtual-Fracture-Carving Simulator was used to help teach trainees the complex acetabular spatial anatomy by drawing fracture lines on a virtual model. Although observation using the simulator is still limited to the two-dimensional (2D) plane and there is a lack of real fractures, this interactive system was considered to promote better understanding of fracture anatomy. | Medicine  acetabular fractures  Specialization training (Residence)  141 students in the first year of three-year standardization resident training from these national training bases were invited to participate | Quantitative  Randomized - Control Trial (RCT)  Participants were equally and randomly assigned to the PM, VR and 3DP learning groups. | Three-level objective tests were conducted to evaluate learning, including identifying anatomical landmarks, describing fracture lines, identifying classification, and inferring fracture mechanism. Four subjective questions were asked to evaluate the usability and value of instructional materials. Generally, the 3DP group showed a clear advantage over the PM and VR groups in objective tests, while there was no significant difference between the PM and VR groups. 3DP was considered to be the most valuable learning tool for understanding acetabular fractures. |
| 77 | Javaux, A., et al. (2018). A mixed-reality surgical trainer with comprehensive sensing for fetal laser minimally invasive surgery. *International journal of computer assisted radiology and surgery*, *13*(12), 1949-1957. | MR  Cognitive/Psychomotor skills | Long training is required to master the instrument manipulation constraints with smaller incisions and reduced surgical trauma in minimally invasive surgery. Very few systems tackle fetal surgery and more specifically the treatment of twin-twin transfusion syndrome. | Medicine surgery  Gynaecology  Specialization training (Residence);Specialists (continuous education)  8 surgeons - three different categories: novice, intermediate and expert. | Quantitative  Usability study  Face and content validity of the developed setup was assessed by asking surgeons from the field of fetal MIS to accomplish specific tasks on the trainer. | A small use-case investigates whether the trainer sensors are able to distinguish between an easy and difficult scenario.  The trainer was deemed sufficiently realistic and its proposed tasks relevant for practicing the required motor skills. The use-case demonstrated that the motion and force sensing capabilities of the trainer were able to analyze surgical skill. |
| 78 | Kay, R., et al. (2018). Assessing the impact of a virtual lab in an allied health program. *Journal of allied health*, *47*(1), 45-50. | VR Virtual lab  Cognitive/Psychomotor skills | Allied health students need exposure to multiple and varied scenarios to improve clinical diagnostic skills, and virtual laboratories could support this process. | Medicine  Medical laboratory  Bachelor  64 students (55 females, 9 males) sampled from a total population of 97 undergraduate students for a response rate of 66%. | Mixed  Usability study  Participants were enrolled in the second (n=35), third (n=17), or fourth year (n=12) of a medical laboratory science program in small suburban Canadian university of 10,000 students. | Students had positive attitudes towards visual learning, authenticity, learner control, organization, and scaffolding afforded by the virtual lab. Challenges reported included navigational difficulties, an absence of control over content selection, and lack of understanding for certain concepts. Over 90% of students agreed that the virtual lab helped them prepare for hands-on laboratory sessions and that they would use this format of instruction again. Overall, 84% of the students agreed that the virtual lab helped them to achieve greater success in learning. |
| 79 | Korzeniowski, P., et al. (2018). VCSim3: a VR simulator for cardiovascular interventions. *International journal of computer assisted radiology and surgery*, *13*(1), 135-149. | VR  Cognitive/Psychomotor skills | VR simulation of cardiovascular procedure can contribute to surgical training and improve the educational experience without putting patients at risk, raising ethical issues or requiring expensive animal or cadaver facilities. | Medicine surgery  cardiology  Specialization training (Residence)  17 participants (15 males and 2 females) | Quantitative  Usability study  Participants were required to complete a cardiovascular intervention five times. Specifically, to navigate the catheter and guidewire from the femoral artery into the heart coronaries, localize the stenosis and deploy a stent. | We present detailed results of simulation accuracy of the virtual instruments, along with their computational performance. In addition, the results of a preliminary face and content validation study conveyed on a group of 17 interventional radiologists are given.  VR simulation of cardiovascular procedure can contribute to surgical training and improve the educational experience without putting patients at risk, raising ethical issues or requiring expensive animal or cadaver facilities. VCSim3 is still a prototype, yet the initial results indicate that it provides promising foundations for further development. |
| 80 | Kugelmann, D., et al. (2018). An augmented reality magic mirror as additive teaching device for gross anatomy. *Annals of Anatomy-Anatomischer Anzeiger*, *215*, 71-77. | AR  Cognitive skills | AR can provide a novel, interactive learning tool in addition to a regular dissection course. Recent studies have shown that integrating radiology into gross anatomy courses significantly improves the learning outcomes for both disciplines. | Medicine anatomy  880 ﬁrst-year medical students | Mixed  Usability study  We designed a tutorial during which students worked with the system in groups of about 12 and evaluated the results. | Each participant was asked to asses the system’s value by filling out a Likert-scale questionnaire. Overall, the students rated the AR MM experience very positively and appreciated the addition of the tool to the gross anatomy course. |
| 81 | Lamb, R., et al. (2018). Comparison of virtual reality and hands on activities in science education via functional near infrared spectroscopy. *Computers & Education*, *124*, 14-26. | VR  Cognitive skills | In the science education as with other fields, aspects of the relationship between positive learning outcomes and technology are often assumed, but scientific evidence is lacking. In fact, use of technology may create an extra level of complexity and hands-on activities may be better. But SEGs and VR may be comparable to hands-on activities. | First year students  Any field - only condition is that participants must not have taken biology or life sciences since enrollment in the university  100 healthy, right-handed college aged students, 53 males and 47 females. | Quantitative  Usability study  This study made use of multiple measures in an eﬀort to characterize the cognitive processing occurring during learning about the topic of DNA replication. The authors used a combination of neuroimaging and content tests to examine the research questions. | The results of this study are in line with previous studies that suggest higher cognitive dynamics, increases in attention, and critical thinking are elicited in subjects engaging in learning through VR and SEGs. Cognitive systems often tie to speciﬁc stimuli and the intensity of activation. This demand may be mediated by diﬀerent modes of presentation, e.g. VR, SEG, video lecture, or hands-on activities, indicating that while the same underlying cognitive architecture may be used to process information related to such activities as critical thinking, modal representations may inﬂuence the degree to which the cognitive systems are used. These outcomes would manifest as intensity of activation and be measured via hemodynamic response using neuroimaging techniques such as fNIRS. |
| 82 | Linsk, A. M., et al. (2018). Validation of the VBLaST pattern cutting task: a learning curve study. *Surgical endoscopy*, *32*(4), 1990-2002. | VR  Cognitive/Psychomotor skills | VR simulators enable objective and automated assessment of performance, in real time, and without the need for proctors. Moreover, they permit unlimited training without the expense of consumables. They can also provide haptic feedback, which has already been shown to be an essential component of minimally invasive surgery simulations. VR-based simulators have been shown to transfer effective technical skills to the operating room environment. | Medicine surgery  laparoscopy  Bachelor- 24 medical students were randomly assigned to an FLS training group, a VBLaST training group, or a control group. | Quantitative  Randomized - Control Trial (RCT)  Fifteen training sessions, 30 min in duration per session per day, were conducted over 3 weeks. All subjects completed pretest, posttest, and retention test (2 weeks after posttest) on both the FLS and VBLaST© simulators. | Performance data, including time, error, FLS score, learning rate, learning plateau, and CUSUM score, were analyzed. The learning curve for all trained subjects demonstrated increasing performance and a performance plateau. CUSUM analyses showed that five of the seven subjects reached the intermediate proficiency level but none reached the expert proficiency level after 150 practice trials. Performance was significantly improved after simulation training, but only in the assigned simulator. No significant decay of skills after 2 weeks of disuse was observed. Control subjects did not show any learning on the FLS simulator, but improved continually in the VBLaST simulator. |
| 83 | Liu, W., et al. (2018). Novel laparoscopic training system with continuously perfused ex-vivo porcine liver for hepatobiliary surgery. *Surgical endoscopy*, *32*(2), 743-750. | VR  Cognitive/Psychomotor skills | Existing models for laparoscopic training, such as box trainers and virtual reality simulators, often fail to provide holistic training and real haptic feedback. We have formulated a new training system that addresses these problems. | Medicine  laparoscopic surgery  Specialists (continuous education)  5 attending surgeons evaluated the developed simulator. Each surgeon had at least 5 years’ experience in performing LCs or had performed at least 500 LC procedures. | Quantitative  Usability study  We assessed the performances of 43 trainees who used the new system to perform laparoscopic cholecystectomy (LC) three times. | Real-Liver Laptrainer offered more functions and better tactile feedback than the FLS or LapSim system. All five surgeons graded the quality of the new system as realistic. The utility of the system for training was scored as 3.6 ± 1.1 on a scale of 1–5. Between the first and third attempts, the number of successfully performed LCs increased (9 vs 14 vs 23; P = .011), while the numbers of liver damage incidents (25 vs. 21 vs. 18, P = .303) and gallbladder perforations decreased (17 vs. 12 vs. 9, P = .163). The mean LC operation time significantly decreased (63 vs. 50 vs. 44, P < .0001). |
| 84 | Llena, C., et al. (2018). Implementation of augmented reality in operative dentistry learning. *European Journal of Dental Education*, *22*(1), 122-130. | AR  Cognitive/Psychomotor skills | A number of requirements must be met in cavity preparation to guarantee adequate resistance, retention and support for the material used, and in this regard, the understanding of cavity design is closely related to the development of spatial visual perception and the calculation of dimensions. AR is a new technology that might be attractive for students and which can serve as a source of study motivation. | Dentistry  Dental students - third year  41 Students enrolled with no prior experience in the preparation of treatment cavities, and no prior experience in the use of AR. | Quantitative  Usability study  Students were divided into two groups (traditional teaching methods vs AR). | No significant differences were observed in level of knowledge before, immediately after or 6 months after teaching between the two groups (P>.05). Although the results corresponding to most of the studied skills parameters were better in the experimental group, significant differences (P<.05) were only founded for cavity depth and extent for Class I and divergence of the buccal and lingual walls for the Class II. The experience was rated as favourable or very favourable by 100% of the participants. The students showed preference for computers (60%) vs mobile devices (10%). |
| 85 | Maresky, H. S., et al. (2019). Virtual reality and cardiac anatomy: Exploring immersive three‐dimensional cardiac imaging, a pilot study in undergraduate medical anatomy education. *Clinical Anatomy*, *32*(2), 238-243. | VR  Cognitive/Affective skills | Cardiac anatomy can be challenging to grasp because of its complex 3D nature and is not easy to teach. In light of some exciting technological advances in the field of virtual reality (VR), we sought to test the viability and the assess efficacy of this computer-generated model for the purposes of teaching cardiac anatomy. | 42 Bachelor 1st-year medical  Cardiac anatomy curriculum | Quantitative  Experimental - Comparison  students participated in an anatomically correct VR simulation of the heart. Students were randomly distributed into 14 control and 28 variable groups (30-min immersive cardiac VR experience). | At the end of the intervention, both the groups underwent a separate post-intervention 10-question quiz. They scored 50.9% on average on the pre-intervention quiz (SD = 16.5) and 70.2% on the post-intervention quiz (SD = 18.7). Compared to the control group, the students exposed to VR scored 21.4% higher in conventional content (P= 0.004), 26.4% higher in VS content (P< 0.001), and 23.9% higher overall (P< 0.001). VR offers an anatomically correct and immersive VS environment that permits learner to interact three-dimensionally with the heart’s anatomy. This study demonstrates the viability and the effectiveness of VR in teaching cardiac anatomy. |
| 86 | Mirghani, I., et al. (2018). Capturing differences in dental training using a virtual reality simulator. *European Journal of Dental Education*, *22*(1), 67-71. | VR  Cognitive/Psychomotor skills | A step change in VR simulation has come from the integration of haptic technology into simulators, as these systems have the potential to provide several advantages over conventional approaches. Advantages include the ability to interact with virtual objects through realistic feel and touch. Haptic technology also provides students with the ability to feel the various tooth surfaces through force feedback mechanisms and distinguish between soft and hard tissues, potentially useful pedagogical information. These haptic systems also automatically produce kinematic data (performance production measures) that could be used for objective assessment of task performance–information that is not available in conventional training environments. Construct validity remains to be studied. | Dental training  Bachelor  289 participants, with 1 (n=92), 3 (n=79), 4 (n=57) and 5 (n=61) years of dental training | Quantitative  Usability study  Participants performed a series of tasks upon their first exposure to the simulator | We found statistically signiﬁcant differences between novice (Year 1) and experienced dental trainees (operationalised as 3 or more years of training), but no differences between performance of experienced trainees with varying levels of experience. This work represents a crucial ﬁrst step in understanding the value of haptic virtual reality simulators in dental education. |
| 87 | Nayar, S. K., et al. (2018). Validation of a virtual reality laparoscopic appendicectomy simulator: a novel process using cognitive task analysis. *Irish Journal of Medical Science (1971-)*, 1-9. | VR  Cognitive/Psychomotor skills | VR simulation offers a controlled environment to practice essential surgical skills and rehearse whole procedures. In addition, immediate detailed feedback can be given, as well as data collected to establish common errors and help improve the quality of training. | Medicine surgery  Laparoscopy  Masters (Interns);Specialization training (Residence)  32 novice and 9 experienced surgeons participated in simulator validation. | Quantitative  Experimental - Comparison  Construct validity was evaluated using dexterity metrics, masked assessment of surgical quality using the OSATS global rating scale, and mental workload from two validated tools: the NASA-TLX and SMEQ. Ten novices performed eight further LAs for learning curve assessment. | Face validity was demonstrated across all domains. Considering content validity, the essential technical and non-technical steps were evident. The experienced group performed the procedure quicker (median time 361 vs. 538 s, P = 0.0039) with fewer total movements (426 vs. 641, P < 0.0001) and shorter idle time (131 vs. 199 s, P = 0.0006). This correlated with higher OSATS scores (median 33.5 vs. 22.2, P < 0.0001) and lower mental demand (NASA-TLX: 9.0 vs. 13.75, P = 0.012; SMEQ: 60 vs. 80, P = 0.0025), indicating construct validity. Learning curve data showed statistically significant improvements after the 7th session for procedure time, total movements and idle time, which correlated with reduction in mental demand. |
| 88 | Nemani, A., et al. (2018). Convergent validation and transfer of learning studies of a virtual reality-based pattern cutting simulator. *Surgical endoscopy*, *32*(3), 1265-1272. | VR  Cognitive/Psychomotor skills | Traditional surgical assessment methods, such as direct observations by an experienced trainer to assess the skills of the trainee, are generally subjective and use global rating scales (GRS) to score competency. There are serious criticisms of creating a generalized rating assessment across all subjects. Such criticism cites tremendous human resource costs, poor interrater reliability of human observers, and poor correlation with technical skill to patient outcome in the operating room. To address the general limitations of physical trainers, virtual reality-based simulators have been developed and shown to provide a safe and effective training and assessment platform for laparoscopic surgical skills. | Medicine  Laparoscopic surgery  Masters (Interns); Specialization training (Residence)  18 medical students at the University at Buffalo, with no prior laparoscopic surgical skills | Quantitative  Randomized - Control Trial (RCT)  Methods Participants: control, FLS training, or VBLaST training groups. Each training group performed pattern cutting trials | Results indicate that the FLS and VBLaST trained groups have significantly higher task performance scores than the control group in both the VBLaST and FLS environments (p < 0.05). Learning curve results indicate that three out of seven FLS training subjects and four out of six VBLaST training subjects achieved the “senior” performance level. Furthermore, both the FLS and VBLaST trained groups had significantly lower transfer task completion times on ex vivo peritoneal tissue models (p < 0.05). |
| 89 | Ng, D. S. C., et al. (2018). impact of virtual reality simulation on learning barriers of phacoemulsification perceived by residents. *Clinical ophthalmology (Auckland, NZ)*, *12*, 885. | VR  Cognitive/Psychomotor skills | With rising patient expectations and demands of high-quality surgical outcomes, it is vital that ophthalmic residents learn to operate in a manner that is safe and time efficient. | Medicine  Ophthalmology  Specialization training (Residence)  22 ophthalmology residents | Quantitative  A cross-sectional, multicenter survey | Nucleus cracking/chopping was ranked highest in difficulty by all respondents followed by capsulorrhexis completion and nucleus rotation/manipulation. Respondents with prior simulation training had significantly lower difficulty scores on these three tasks (nucleus cracking/chopping; capsulorrhexis completion; and nucleus rotation/manipulation). In multivariate analyses, simulation training was significantly associated with lower difficulty scores on these three tasks. |
| 90 | Nomura, T., et al. (2018). Characteristics predicting laparoscopic skill in medical students: nine years’ experience in a single center. *Surgical endoscopy*, *32*(1), 96-104. | VR  Cognitive skills | Important factors in preventing problems attributable to primitive techniques of laparoscopic surgery are training and skill assessment outside the operating room, often performed with the use of various types of simulators. Both virtual reality simulators (VRS) and augmented reality simulators (ARS) have been adopted for laparoscopic surgery in many institutes. The utility of these simulators in the surgical education of medical students and novice surgeons has been reported by many studies. | Medicine  laparoscopic surgery  Masters (Interns)  In total, 270 fifth-year medical students performed ARS training after interviews. | Quantitative  Usability study  Multiple regression analysis identified significant effects of manual dexterity, gender, and confidence about driving on the results of the training. | This study investigated factors likely to be predictors of the laparoscopic skill of medical students. Changes over the course of time and variability among the results in each training group were also evaluated. Five variables (desire to be a surgeon, perfectionist, like video games, confidence in driving, and manual dexterity) were significantly correlated with at least some of the results of training in the univariate analyses. The multiple regression analyses suggested that manual dexterity, gender, and confidence in driving significantly contributed to the results of training. As for the disparity between generations, the mean training results have been significantly improving in recent years. |
| 91 | Perin, A., et al. (2018). Filling the gap between the OR and virtual simulation: a European study on a basic neurosurgical procedure. *Acta Neurochirurgica* *160*(11): 2087-2097. | VR  Cognitive/Psychomotor skills | Placing an external ventricular drain (EVD) - In this regard, virtual reality simulators— with haptic feedback technology—might be able to create a realistic and safe environment, where both residents and surgeons could train and practice, in order to perform this procedure better and more safely. | Medicine surgery  Neurosurgical  92 participants [47 males and 45 females; average age of 32 years old (min 26; max 53); 25 junior residents, 49 senior residents and 18 staff neurosurgeons from different European countries were involved in the study. | Quantitative  Usability study | Data suggests a positive correlation between subjects who placed more EVDs in the previous year and those who get better scores at the simulator (p = .008) (fewer attempts and better surgical accuracy). The number of attempts to reach the ventricle was also analysed; senior residents needed fewer attempts (mean = 2.26; SD = 1.11) than junior residents (mean = 3.12; SD = 1.05) (p = .007) and staff neurosurgeons (mean = 2.89, SD = 1.23). Scoring results were compared by using the Fisher’s test, for the analysis of the variances, and the Student’s T test. Surprisingly, having a wider surgical experience overall does not correlate with the best performance at the simulator. |
| 92 | Pulijala, Y., et al. (2018a). An innovative virtual reality training tool for orthognathic surgery. *International journal of oral and maxillofacial surgery*, *47*(9), 1199-1205. | VR  Cognitive/Psychomotor skills | VR surgery is a holistic learning application that provides an uninterrupted close-up surgical training experience. | Medicine  Orthographic surgery  Specialists (continuous education)  9 consultant surgeons volunteered to participate in the validation process. | Quantitative  Usability study  a novel training tool for Le Fort I osteotomy based on immersive virtual reality (iVR) was developed and validated | Seven consultant oral and maxillofacial surgeons evaluated the application for face and content validity. Using a structured assessment process, the surgeons commented on the content of the developed training tool, its realism and usability, and the applicability of VR surgery for orthognathic surgical training. The results confirmed the clinical applicability of VR for delivering training in orthognathic surgery. |
| 93 | Pulijala, Y., et al. (2018b). Effectiveness of immersive virtual reality in surgical training—A randomized control trial. *Journal of Oral and Maxillofacial Surgery*, *76*(5), 1065-1072. | VR  Cognitive/Affective skills | The lack of conﬁdence in novices can lead to unintended mishaps during surgery. Immersive technologies, including VR and AR, might provide an answer for these challenges. | Medicine  Dental surgery  Specialization training (Residence)  95 residents from 7 dental schools were included. | Quantitative  Randomized - Control Trial (RCT) | The study group participants showed signiﬁcantly greater perceived self-conﬁdence levels compared with those in the control group (P = .034; a= 0.05). Novices in the ﬁrst year of their training showed the greatest improvement in their conﬁdence compared with those in their second and third year. |
| 94 | Rai, A., et al. (2018). Patient-specific Virtual Simulation—A State of the Art Approach to Teach Renal Tumor Localization. *Urology*, *120*, 42-48. | VR  Cognitive skills | To test whether a novel visuospatial testing platform improves trainee ability to convert two-dimensional to three-dimensional (3D) space. | Medicine  Image Reconstruction and Virtual Model Generation  100 Bachelor-medical students (years 1-4) were recruited as subjects | Quantitative  Randomized - Control Trial (RCT)  dV-Trainer (intervention) and No-dV-Trainer (control) | Tasks (1) visualization of computed-tomographic images, (2) visualization of the reconstructed kidney and tumor in the dV-Trainer (intervention group only), and (3) selection of the correct tumor location on the 3D printed models (primary outcome). dV-Trainer use significantly improved subjects ability to localize tumor position (tumor localization score: 0.24 vs 0.38, P < .001). However, subjects in the No-dV-Trainer group more accurately assigned R.E.N.A.L. scores. |
| 95 | Raison, N., et al. (2018). Cognitive training for technical and non‐technical skills in robotic surgery: a randomised controlled trial. *BJU international*, *122*(6), 1075-1081. | VR  Cognitive/Psychomotor skills | Expansion of simulation training has allowed adoption of the deliberate practice model of training centred on repeated, effortful practice directed through feedback to improve performance. The wide variety of simulation tools now available has enabled training to move towards a more individualised, proficiency-based approach. | Medicine  Minimally invasive surgery (MIS)  Bachelor;Masters (Interns)  Participants were recruited from three large London hospital trusts and four associated medical schools by open invitation | Quantitative  Randomized - Control Trial (RCT)  A single‐blind, parallel‐group randomised controlled trial was conducted at the Vattikuti Institute of Robotic Surgery, King's College London. | Results In all, 33 participants underwent MI training and 29 underwent standard training. Interrater reliability was high, Krippendorff's α = 0.85. After MI training, the mean (sd) GEARS score was significantly higher than after standard training, at 13.1 (3.25) vs 11.4 (2.97) (P = 0.03). There was no difference in mean NOTSS scores, at 25.8 vs 26.4 (P = 0.77). MI training was successful with significantly higher imagery scores than standard training (mean MIQ score 5.1 vs 4.5, P = 0.04). Conclusions - Motor imagery is an effective training tool for improving technical skill in MIS even in novice participants. No beneficial effect for NTS was found. |
| 96 | Rojas-Muñoz, E., et al. (2018). Surgical Telementoring Without Encumbrance: A Comparative Study of See-through Augmented Reality-based Approaches. *Annals of surgery*. | AR  Cognitive/Psychomotor skills | To improve surgical telementoring. Surgical telementoring is a method to deliver specialized expertise to a mentee in scenarios where expertise is not readily available. For example, telementoring can allow a remote expert surgeon to convey specialized surgical expertise to a generalist surgeon in rural hospitals, in disaster-affected regions and in the battlefield. AR can make this more effective, for example by overlaying mentor annotations onto the mentee’s view of the surgical field. | Medicine  Surgery abdominal incision  20 medical students (6 females, 14 males) in their 2^nd^, 3^rd^, or 4^th^ year, and had no previous experience with surgical telementoring systems. | Quantitative  Usability study  20 medical students performed anatomical marking (Task1) and abdominal incision (Task2) on a patient simulator, in 1 of 2 telementoring conditions: ARHMD and telestrator. | The ARHMD condition yielded smaller placement errors (Task1: 45%, P < 0.001; Task2: 14%, P = 0.01), fewer focus shifts (Task1: 93%, P < 0.001; Task2: 88%, P = 0.0039), and longer completion times (Task1: 31%, P < 0.001; Task2: 24%, P = 0.013). Furthermore, the ARHMD avoided potential tablet collisions (4.8 for 3.2 seconds in Task1; 3.8 for 1.3 seconds in Task2). |
| 97 | Sgouros, N. P., et al. (2018). An automated skills assessment framework for laparoscopic training tasks. *The International Journal of Medical Robotics and Computer Assisted Surgery*, *14*(1), e1853. | MR  Laparoscopy Box Trainer Cognitive/Psychomotor skills | In laparoscopic surgery, new challenges arise and additional skills are required from the surgeon through a demanding learning process in terms of procedures and manoeuvers. In order to facilitate these needs, various simulation setups are developed, ranging from simplistic box trainers to virtual and augmented reality simulators. | Medicine  Surgery  Specialization training (Residence).  Novices and Experts | Quantitative  Usability study  Novices- included residents at the beginning of their residency training, Experienced- surgeons who are significantly more experienced in laparoscopic operations | The system achieves an average of 96% correct classification ratio (CCR) when no information on the performed task is available and >98% CCR when the task is known, outperforming a recently proposed video‐based technique by >13%. |
| 98 | Sugiura, A., et al. (2018). The Use of Augmented Reality Technology in Medical Specimen Museum Tours. *Anatomical sciences education*. | AR  Cognitive skills | In particular, these modalities have been applied to anatomical and pathological education, especially with regards to understanding the 3D structure of the body in physiologic and pathologic states. Such technologies may also bridge many of the difficulties in the upkeep and use of medical museums in medical education. Previous studies have suggested that such systems not only enhance learners’ motivation, but also their capacity for independent learning. | Medicine  60 Bachelor - studying anatomy in nursing course. Another 60 2^nd^‐year medical students studying anatomy | Quantitative  Usability study  Groups: Control, Tablet AR, and HMD AR | Participants evaluated the AR tracking and display devices. Significant differences were found among the three groups (χ2 = 19.95, P < 0.001). The Wilcoxon multiple comparison showed that the total scores for both AR groups were significantly higher than that of the control group (P < 0.001 and P = 0.018 for control vs. tablet AR and control vs. HMD AR, respectively), whereas no significant difference was found in the scores between the two AR groups (P = 0.247). These results suggest that both AR groups could improve learning achievements regardless of the difference in the device. |
| 99 | Won, T. B., et al. (2018, January). Early experience with a patient‐specific virtual surgical simulation for rehearsal of endoscopic skull‐base surgery. *International forum of allergy & rhinology* *8*(1), 54-63. | VR  Cognitive/Psychomotor skills | Virtual reality (VR) surgical simulation has the potential to shorten learning curves and ultimately improve surgical care by representing specific anatomic configuration as well as avoiding the considerable resources that are needed in cadaver dissections. | Medicine  Endoscopic skull‐base surgery  Specialization training (Residence)  10 patients | Quantitative  We conducted a retrospective analysis of the ability of the rhinologic VSE to replicate intraoperative ﬁndings (face validity). | In all 10 cases, the simulation experience was realistic enough to perform dissections in a similar manner as in the actual surgery. Excellent correlation was found in terms of surgical exposure, anatomical features, and the locations of pathology. |
| 100 | Yiasemidou, M., et al. (2018). Patient-specific mental rehearsal with interactive visual aids: a path worth exploring? *Surgical endoscopy*, *32*(3), 1165-1173. | VR  Cognitive skills | Students need sufficient training opportunities in order to become sufficiently proficient in surgery. | Medicine surgery  Laparoscopic cholecystectomy  Bachelor; Masters (Interns)  16 medical students, (years 2–5) | Quantitative  Randomized - Control Trial (RCT)  Group A practiced mental rehearsal with the use of a pre-prepared checklist and Group B mental rehearsal with the checklist combined with virtual models matching the anatomical variations of the SLCs. | The participants performed equally well when presented with a “straight-forward” anatomy [Group A vs. Group B—time sec: 445.5 vs. 496 p = 0.64—NOM: 437 vs. 413 p = 0.88—PL cm: 1317 vs. 1059 p = 0.32—per: 0.5 vs. 0 p = 0.22—NCB: 0 vs. 0 p = 0.71—DVS: 0 vs. 0 p = 0.2]; however, Group B performed significantly better [Group A vs. B Total CAT score—Short Cystic Duct (SCD): 20.5 vs. 26.31 p = 0.02 η 2 = 0.32—Double cystic Artery (DA): 24.75 vs. 30.5 p = 0.03 η 2 = 0.28] and committed less errors (Damage to Vital Structures—DVS, SCD: 4 vs. 0 p = 0.03 η 2=0.34, DA: 0 vs. 1 p = 0.02 η 2 = 0.22) in the cases with more challenging anatomies. |
| 101 | Yoganathan, S., et al. (2018). 360 virtual reality video for the acquisition of knot tying skills: a randomised controlled trial. *International Journal of Surgery*, *54*, 24-27. | VR  Cognitive skills | In modern day surgical training, reduced working hours, rota pressures and the increasing use of alternative treatment modalities such as interventional radiology are changing a trainee’s exposure to certain procedures. Surgical trainees encounter numerous obstacles to time spent in the operating theatre enhancing their surgical skills. Virtual reality technology has the potential to bridge those obstacles, bring close to real life experiences, and accelerate learning curves. | Medicine  Surgery  Masters (Interns)  40 foundation year doctors (ﬁrst year postgraduate) were randomised to either the 360-degree VR video (n=20) or 2D video teaching (n=20). | Quantitative  Randomized - Control Trial (RCT) | Knot tying scores were signiﬁcantly better in the VR video teaching arm when compared with conventional (median knot score 5.0 vs 4.0 p = 0.04). When used in combination with face-to-face skills teaching this diﬀerence persisted (median knot score 9.5 vs 9.0 p = 0.01). More people in the VR arm constructed a complete reef knot than in the 2D arm following face-to-face teaching (17/20 vs 12/20). No diﬀerence between the groups existed in the time taken to construct a reef knot following video and teaching (median time 31.0s vs 30.5s p = 0.89). |
|  |  |  |  |  |  |  |

Table 5. Overview of the use of AR/VR/MR in research in 2019 and their methodological quality to train students and professionals in medicine, biomedical and health sciences (n=33).

|  | **Title of article** | **AR/VR/MR**  **Learning & behavioral outcomes -Bloom’s taxonomy** | **What is the rationale for AR/VR/MR exposure** | **In which domain of healthcare are AR/VR/MR being used? Who are the participants?** | **What type of design/methodology is used in these studies?** | **What are the findings?** |
| --- | --- | --- | --- | --- | --- | --- |
|  |  |  |  |  |  |  |
| 102 | Alismail, A., et al. (2019). Augmented reality glasses improve adherence to evidence-based intubation practice. *Adv Med Educ Pract, 10*, 279-286. | AR  Psychomotor skills | Facilitate simulation | Medicine  A total of 32 subjects:  8 medical students, 8 medical professors, 8 unskilled doctors and 8 skilled doctors group. | Quantitative  Experimental - Comparison (control) | The AR glasses showed promise in assisting different health care professionals on endotracheal intubation simulation. Participants in the AR group took a longer time to ventilate but scored 100% in the developed checklist that followed the NEJM protocol. |
| 103 | Bayram, S. B., et al. (2019). Effect of a game-based virtual reality phone application on tracheostomy care education for nursing students: A randomized controlled trial. *Nurse Educ Today, 79*, 25-31. | VR  Psychomotor skills | Offer training situations | Medicine  nursing  238 first-year nursing students | Quantitative  Randomized - Control Trial (RCT) | The game-based virtual reality phone application was effective in teaching the skill of suctioning a tracheostomy tube for nursing students in the short term, and it is recommended that this application be used in psychomotor skill training. |
| 104 | Cold, K. M., et al. (2019). Simulation-Based Mastery Learning of Flexible Bronchoscopy: Deciding Factors for Completion. *Respiration, 97*(2), 160-167. | VR  Cognitive skills | Offer training situations | Medicine  77 trainees (doctors from different specialties, respiratory medicine, thoracic surgery, otorhinolaryngology and anesthesiology). | Quantitative  Case control study | Successful simulation-based mastery learning courses should be clinically relevant, and the trainees should be provided protected time to complete the training. The instructional design should also be adapted systematically for male and female trainees to achieve the necessary competencies. |
| 105 | Dardick, J., et al. (2019). Virtual reality simulation of neuroendovascular intervention improves procedure speed in a cohort of trainees. *Surg Neurol Int, 10*, 184. | VR  Psychomotor skills | Decrease training time | Medicine  Surgery training  18 trainees | Quantitative  Usability study (checking to see if the study/tool aligns with what you want to teach/learn) | Both performance and viewing of simulated procedures produced significant decreases in time to reach neuroendovascular procedural benchmarks. These data show that VR simulation is a valuable tool for improving trainee skill in neuroendovascular procedures. |
| 106 | Erolin, C., et al. (2019). Using virtual reality to complement and enhance anatomy education. *J Vis Commun Med, 42*(3), 93-101. | VR  Psychomotor skills | More authentic | Medicine  18 students: 5 participants from 2nd year medicine, 5 MSc medical art students, 4 PhD students, 2 MSc anatomy and forensic anthropology students, 1 MSc forensic art and facial identification student and 1 undergraduate forensic anthropology student. | Qualitative  Usability study (checking to see if the study/tool aligns with what you want to teach/learn) | VR models receiving a slightly more favourable response than those online in terms of how much participants enjoyed viewing the models. |
| 107 | Frendo, M., et al. (2019). Decentralized virtual reality mastoidectomy simulation training: a prospective, mixed-methods study. *Eur Arch Otorhinolaryngol, 276*(10), 2783-2789. | VR  Psychomotor skills | Offer training situations | Medicine  Surgery simulation  20 participants had an otorhinolaryngology training. | Mixed  Posttest only | Decentralized VR training using a freeware, high-fidelity mastoidectomy simulator is feasible but did not lead to a high training volume or truly distributed practice. |
| 108 | Gustafsson, A., et al. (2019). Hip-fracture osteosynthesis training: exploring learning curves and setting proficiency standards. *Acta Orthop, 90*(4), 348-353. | VR  Psychomotor skills | Offer training situations Learn in a safe environment | Medicine  Training surgery operation 51 novices in their 1st year of specialization were included from 7 different departments. | Quantitative  Experimental - Comparison (control) | Training time to reach plateau varied widely and it is paramount that simulation-based training continues to a predefined standard instead of ending after a fixed number of attempts or amount of time. |
| 109 | Hanson, J., et al. (2019). Effectiveness of three-dimensional visualisation on undergraduate nursing and midwifery students' knowledge and achievement in pharmacology: A mixed methods study. *Nurse Educ Today, 81*, 19-25. | VR  Cognitive skills | Offer training situations | Nursing  226 1st-year students enrolled in the Bachelor of Nursing Science degree or Bachelor of Nursing Science and Midwifery dual degree. | Mixed  Pretest-Posttest | The three-dimensional experience improved understanding when compared to two-dimensional viewing, satisfied students leaning needs, and caused minimal discomfort. |
| 110 | Jensen, K., et al. (2019). Evaluating competency in video-assisted thoracoscopic surgery (VATS) lobectomy performance using a novel assessment tool and virtual reality simulation. *Surg Endosc, 33*(5), 1465-1473. | VR  Psychomotor skills | Offer training situations | Medicine  surgery  53 participants with varying experience in VATS lobectomy. | Quantitative  Usability study (checking to see if the study/tool aligns with what you want to teach/learn) | ATSAT provides supervisors and assessors with a procedure-specific assessment tool for evaluating VATS lobectomy performance and aids with the decision of when the trainee is ready for unsupervised performance. |
| 111 | Kim-Berman, H., et al. (2019). Validity and User Experience in an Augmented Reality Virtual Tooth Identification Test. *J Dent Educ, 83*(11), 1345-1352. | AR  Cognitive/Affective skills | Offer training situations | Medicine  dentistry  109 first-year dental students | Mixed  Usability study (checking to see if the study/tool aligns with what you want to teach/learn) | On the tests, the students had some difficulty in viewing and manipulating the images and experienced technical difficulties related to their smartphones, and their survey responses expressed little support for the AR tool. Nevertheless, this study demonstrated criterion validity of the AR virtual assessment tool for tooth identification. |
| 112 | Kowalewski, K. F., et al. (2019). One or two trainees per workplace for laparoscopic surgery training courses: results from a randomized controlled trial. *Surg Endosc, 33*(5), 1523-1531. | VR  Psychomotor skills | Offer training situations | Medicine  surgery 100 medical student were included: (alone = 40; dyad = 40; control = 20). | Quantitative  This study was a registered prospective, single-center, rater- blinded, three-arm, parallel-group randomized controlled trial. | The curriculum provided trainees with the laparoscopic skills needed to perform LC safely, irrespective of the number of trainees per workplace. Dyad training reduced the operation time needed for LC. |
| 113 | Liaw, S. Y., et al. (2019). Design and evaluation of a 3D virtual environment for collaborative learning in interprofessional team care delivery. *Nurse Educ Today, 81*, 64-71. | VR  Cognitive/Affective skills | Offer training situations | Medicine  Interprofessional skills  29 healthcare students (e.g. medical social worker and pharmacist). | Mixed  Pretest-Posttest | Finding supports the feasibility of using a 3D-VE in supporting social interactions and collaborative practices among the six different healthcare professions to facilitate the sharing of information and the planning of patient care. |
| 114 | Lindquist, N. R., Leach, M., Simpson, M. C., & Antisdel, J. L. (2019). Evaluating Simulator-Based Teaching Methods for Endoscopic Sinus Surgery. *Ear Nose Throat J, 98*(8), 490-495. | VR  Psychomotor skills | Offer training situations | Medicine Surgery  34 first-year medical students | Quantitative  Experimental - Comparison (control) | Found no difference in performance of novice trainees with regard to basic anatomical identification or procedural tasks associated with ESS, the use of ESS simulators may improve time to completion. |
| 115 | Lorenzo-Alvarez, R., Rudolphi-Solero, T., Ruiz-Gomez, M. J., & Sendra-Portero, F. (2019). Medical Student Education for Abdominal Radiographs in a 3D Virtual Classroom Versus Traditional Classroom: A Randomized Controlled Trial. *AJR Am J Roentgenol, 213*(3), 644-650. | VR  Cognitive/Affective skills | Motivation | Health Sciences  215 students: The students were randomized into two groups of 107 (VW group) and 108 (RL group). | Quantitative  Pretest-Posttest | Radiology education in a 3D virtual classroom fosters participatory learning and results in similar acquisition of interpretive skills as a traditional face-to-face classroom. Virtual worlds allow the performance of online activities to learn interpretive skills with guaranteed success in learning similar to that of conventional activities. Additionally, the relative lack of identity in the virtual workshops makes students less afraid to speak and more participatory. |
| 116 | Makransky, G., et al. (2019). Equivalence of using a desktop virtual reality science simulation at home and in class. *PloS one, 14*(4), e0214944. | VR  Cognitive skills | Offer training situations | Microbiology  112 students: the topic of microbiology at home on their own time (home group; N = 62) or in an assigned classroom setting with teacher supervision (classroom group; N = 50). | Quantitative  Experimental - Comparison (control) | The home and classroom groups did not differ significantly on post- test learning outcome scores, or on self-report measures of intrinsic motivation or self-efficacy. |
| 117 | Maresky, H. S., et al. (2019). Virtual reality and cardiac anatomy: Exploring immersive three-dimensional cardiac imaging, a pilot study in undergraduate medical anatomy education. *Clin Anat, 32*(2), 238-243. | VR  Psychomotor skills | Effective way of teaching, better than 3D models. offers an immersive and intuitive experience | Medicine  anatomy  261 medical students | Quantitative  Usability study (checking to see if the study/tool aligns with what you want to teach/learn) | VR offers an anatomically correct and immersive VS environment that permits learner to interact three-dimensionally with the heart’s anatomy. |
| 118 | Ochs, M., et al. (2019). Training doctors’ social skills to break bad news: evaluation of the impact of virtual environment displays on the sense of presence. *Journal on Multimodal User Interfaces, 13*(1), 41-51. | VR  Cognitive skills | Create a sense of presence through immersion | Health Sciences  22 persons | Quantitative  Usability study (checking to see if the study/tool aligns with what you want to teach/learn) | This article described a semi-autonomous system which particularly suitable for doctors to break bad news. |
| 119 | Sattar, M. U., et al. (2019). Effects of Virtual Reality training on medical students' learning motivation and competency. *Pak J Med Sci, 35*(3), 852-857. | VR  Psychomotor skills | Offer training situations | Medicine  87 volunteers | Quantitative  Usability study (checking to see if the study/tool aligns with what you want to teach/learn) | Virtual reality was found best for medical students in both learning motivation and learning competency. Medical students and educationist may select virtual reality as new learning methodology for curriculum learning. |
| 120 | Savran, M. M., et al.(2019). Using virtual-reality simulation to ensure basic competence in hysteroscopy. *Surg Endosc, 33*(7), 2162-2168. | VR  Cognitive skills | Create a sense of presence through immersion | Medicine  Three groups of distinct experience levels were enrolled: medical students; residents with limited hysteroscopy experience (1–50 hysteroscopy procedures); and experienced gynaecologists. We intended to include more than 10 participants per group. | Quantitative  Experimental - Comparison (control) | This study developed a standardised, simulation-based test of competence in hysteroscopy and established arguments of validity evidence for the test. |
| 121 | Schlosser, P. D., et al. (2019). An exploratory clinical evaluation of a head-worn display based multiple-patient monitoring application: impact on supervising anesthesiologists' situation awareness. *J Clin Monit Comput, 33*(6), 1119-1127. | AR  Affective outcomes | Offer training situations | Medicine  8 anesthesiologists | Qualitative  Proof of concept study | Head-worn displays can improve supervising anesthesiologists’ situation awareness in multiple-patient monitoring situations. |
| 122 | Schmidt, M. W., et al. (2019). The Heidelberg VR Score: development and validation of a composite score for laparoscopic virtual reality training. *Surg Endosc, 33*(7), 2093-2103. | VR  Psychomotor skills |  | Medicine surgery  Participants totaled to 24 LapMentor™ II and 23 on the LapMentor™ III, with Expert groups of 9 per simulator. | Quantitative  Develop VR score for laparoscopic surgical training | N effective and simple performance measurement was established to propose a new standard in analyzing and reporting VR outcome data—the Heidelberg virtual reality (VR) score. |
| 123 | Shirk, J. D., et al. (2019). The Use of 3-Dimensional, Virtual Reality Models for Surgical Planning of Robotic Partial Nephrectomy. *Urology, 125*, 92-97. | VR  Psychomotor skills | Offer training situations | Medicine  3 surgeons: The intervention group included patients who were scheduled to undergo robotic-assisted laparoscopic partial nephrectomy, identified from the operating room schedule. Patients were accrued in chronological order for a total of 30 cases. The control group was identified from the surgical schedule as patients having undergone robotic assisted laparoscopic partial nephrectomy starting 6 months prior to the intervention implementation and continued in chronological order until 30 eligible cases had been identified. | Quantitative  Experimental - Comparison (control) | Use of a 3-dimensional, virtual reality model when performing robotic partial nephrectomy improves key surgical outcome parameters. |
| 124 | Sultan, L., et al. (2019). An Experimental Study On Usefulness Of Virtual Reality 360 degrees In Undergraduate Medical Education. *Adv Med Educ Pract, 10*, 907-916. | VR Affective outcomes | Offer training situations | Medicine  492 students | Quantitative  Pretest-Posttest | VR provides a rich, interactive, and engaging educational context that supports experiential learning-by-doing. In fact, it raises interest and motivation for student and effectively supports knowledge retention and skills acquisition. |
| 125 | Takagi, D., et al. (2019). Effects of dental students’ training using immersive virtual reality technology for home dental practice. *Educational Gerontology, 45*(11), 670-680. | VR  Cognitive skills | Reality teaching material | Medicine  dentistry  101 students | Mixed  Pretest-Posttest | Findings indicate that VR teaching material can supplement dental students’ training in home dental practice. |
| 126 | Van der Heijden, L. L. M., et al. (2019). Validation of Simendo Knee Arthroscopy Virtual Reality Simulator. *Arthroscopy, 35*(8), 2385-2390. | VR  Psychomotor skills | Offer training situations | Medicine  All residents, staff, and medical masters students of the orthopaedic residency program ROGO-Zuid were approached. | Quantitative  Experimental - Comparison (control) | Based on the results, this knee simulator can be applied to train the basic arthroscopic hand-eye coordination skills at the start of resident education programs. |
| 127 | Vera, J., Diaz-Piedra, C., et al. (2019). Intraocular pressure increases after complex simulated surgical procedures in residents: an experimental study. *Surg Endosc, 33*(1), 216-224. | VR  Psychomotor skills | Offer training situations | Medicine surgery  The participants were 34 members of the Andalusian health-care system. 17 surgical and medical residents comprised the experimental group; control group of 17 health care professionals. | Quantitative  Experimental - Comparison (control) | Our data show, for the first time, that IOP is sensitive to residents’ task load, and it could be used as a new index to easily and rapidly assess task (over)load in healthcare scenarios. An arousal-based explanation is given to describe IOP variations due to task complexity. |
| 128 | Wilson, E., et al. (2019). Improved laparoscopic skills in gynaecology trainees following a simulation-training program using take-home box trainers. *Aust N Z J Obstet Gynaecol, 59*(1), 110-116. | VR  Psychomotor skills |  | Medicine  gynaecology  33 participants | Quantitative  Experimental - Comparison (control) | A take- home box trainer simulation- training program was associated with improvement in laparoscopic skills. This type of program may improve trainee access to simulation training. |
| 129 | Wong, D. T., et al. (2019). The effect of virtual reality bronchoscopy simulator training on performance of bronchoscopic-guided intubation in patients: A randomised controlled trial. *Eur J Anaesthesiol, 36*(3), 227-233. | VR  Psychomotor skills | Offer training situation | Medicine  Medical students, anaesthesia assistants and anaesthesia residents | Quantitative  Randomized - Control Trial (RCT) | They found posttraining performance of FOB intubation, as measured by intubation time and Global Rating Scale (GRS), improved in Group (simulation) SIM, while it was unchanged in the Group (control)CON. The ORSIM simulator may be a useful adjunct in acquiring FOB intubation skills. |
| 130 | Zackoff, M. W., et al. (2019). Medical Student Perspectives on the Use of Immersive Virtual Reality for Clinical Assessment Training. *Acad Pediatr, 19*(7), 849-851. | VR  Cognitive skills | Offer training situations | Medicine  A randomized sample of third-year medical students, based upon predetermined clinical team assignment during their pediatric rotation, was invited to participate in a VR curriculum. | Quantitative  Prospective pilot study// post test only | Medical students reported an immersive virtual reality (VR) curriculum on respiratory distress as clinically accurate and likely to impact future patient assessment. VR training was rated as equally or more effective than high-fidelity mannequins and standardized patients but less effective than bedside teaching. |
| 131 | Zare Bidaki, M., et al. (2019). Designing, Producing, Application, and Evaluation of Virtual Reality-Based Multimedia Clips for Learning Purposes of Medical and Nursing Students. *Chest, 155*(4), 166A. | VR  Cognitive skills | Offer training situations | Medicine  47 medical students | Quantitative  Randomized - Control Trial (RCT)  BIOMEDICAL SCIENCE | Virtual reality-based simulation is a successful complementary method to teach pathogenesis of respiratory system infection to medical students. |
| 132 | Zhou, Z., Hu, et al. (2019). Feasibility of Virtual Reality Combined with Isocentric Navigation in Transforaminal Percutaneous Endoscopic Discectomy: A Cadaver Study. *Orthop Surg, 11*(3), 493-499. | VR  Psychomotor skills | Offer training situations | Medicine  12 cadaver specimens were included in the study and four surgeons manually conducted procedures. | Quantitative  Experimental - Comparison (control) | Virtual reality combined with isocentric navigation is feasible in TPED. It enables precise surgical planning and improves intraoperative procedures, and has the potential for application in clinical practice. |
| 133 | Zhou, Z. Y., et al. (2019). Personalized planning and training system for brachytherapy based on virtual reality. *Virtual Reality, 23*(4), 347-361. | VR  Psychomotor skills | Offer training situations | Medicine  A total of 32 subjects:  8 medical students, 8 medical professors, 8 unskilled doctors and 8 skilled doctors group | Quantitative  Usability study (checking to see if the study/tool aligns with what you want to teach/learn) | The study was designed to assess user satisfaction with the traditional training system and the immersive training system. In comparison with the traditional training system, the immersive training system showed higher levels of usability, learnability and enjoyment for all categories of participants, who differed based on age and job. |
|  |  |  |  |  |  |  |

Table 6. Overview of the use of AR/VR/MR in research in 2020 and their methodological quality to train students and professionals in medicine, biomedical and health sciences (n=51).

|  | **Title of article** | **AR/VR/MR**  **Learning & behavioral outcomes -Bloom’s taxonomy** | **What is the rationale for AR/VR/MR exposure** | **In which domain of healthcare are AR/VR/MR being used? Who are the participants?** | **What type of design/methodology is used in these studies?** | **What are the findings?** |
| --- | --- | --- | --- | --- | --- | --- |
|  |  |  |  |  |  |  |
| 134 | Aussedat, C., et al. (2020). Using virtual reality in audiological training: Our experience in 22 otolaryngology residents. *Clin Otolaryngol, 45*(4), 643-648. | VR  Cognitive skills | Offer training situations | Medicine  22 ENT residents | Quantitative  Usability study (checking to see if the study/tool aligns with what you want to teach/learn) | The mean rating by expert educators was 4.4/5 (±0.3), indicating satisfactory validity; student examination scores significantly improved after completing the VR training (P < .0001). |
| 135 | Azzam, N., et al. (2020). Development and validation of metric-based-training to proficiency simulation curriculum for upper gastrointestinal endoscopy using a novel assessment checklist. *Saudi J Gastroenterol*. | VR  Cognitive skills | Offer training situations | Medicine  5 experienced and 10 novice endoscopists | Quantitative  Usability study (checking to see if the study/tool aligns with what you want to teach/learn) | The Fundamentals of Gastrointestinal Endoscopy simulation training curriculum and its SESAS global assessment tool have been primarily validated and can serve as a valuable addition to the gastroenterology fellowship programs. |
| 136 | Baran, B., et al. (2020). Reproductive System Augmented Reality Application for Sexual Health Classes. *International Journal of Sexual Health, 32*(4), 408-420. | AR  Cognitive skills | Offer training situations | Health sciences  16 counselor candidates | Qualitative  Pretest-Posttest | A significant improvement from pretest to post-test indicated that the AR application enhanced participants’ knowledge of reproductive organs and their positions. Participants learned more about female reproductive systems than male reproductive systems. |
| 137 | Bartlett, J. D., et al. (2020). The learning curves of a validated virtual reality hip arthroscopy simulator. *Arch Orthop Trauma Surg, 140*(6), 761-767. | VR  Psychomotor skills | Offer training situations | Medicine  25 medical students | Quantitative  learning curve | The results of this study demonstrate learning curves for a hip arthros copy simulator, with significant improvements seen after three sessions. All performance metrics were found to improved, demonstrating sufficient visuo-haptic consistency within the virtual environment, enabling individuals to develop basic arthroscopic skills. |
| 138 | Berg, H., et al. (2020). Is individual practice in an immersive and interactive virtual reality application non-inferior to practicing with traditional equipment in learning systematic clinical observation? A randomized controlled trial. *BMC Med Educ, 20*(1), 123. | VR  Psychomotor skills | Offer training situations | Nursing  First year medical and nursing students | Quantitative  Randomized - Control Trial (RCT) | Individual self-practicing the ABCDE approach in VR was non-inferior to individual self-practicing with traditional equipment. |
| 139 | Bogomolova, K., et al (2020). The Effect of Stereoscopic Augmented Reality Visualization on Learning Anatomy and the Modifying Effect of Visual-Spatial Abilities: A Double-Center Randomized Controlled Trial. *Anat Sci Educ, 13*(5), 558-567. | AR&VR  Cognitive skills | Offer training situations | Medicine  Participants were a volunteer sample of first- and second-year undergraduate students of Medicine and Biomedical Sciences . | Quantitative  Randomized - Control Trial (RCT) | The Effect of Stereoscopic Augmented reality Visualization on Learning Anatomy and the modifying Effect of Visual-Spatial Abilities: A Double-center randomized controlled trial. |
| 140 | Chen, S., et al. (2020). Can virtual reality improve traditional anatomy education programmes? A mixed-methods study on the use of a 3D skull model. *BMC Med Educ, 20*(1), 395. | VR  Cognitive skills | Offer training situations | Medicine  74 clinical undergraduates from PUMC | Mixed  Pretest-Posttest | This paper presents a study in which the educational effectiveness of a virtual reality (VR) skull model is compared with that of cadaveric skulls and atlases. The aim of this study was to compare the results of teaching with VR to results of teaching with traditional teaching methods by administering objective questionnaires and perception surveys. |
| 141 | Chheang, V., et al. (2020). Toward interprofessional team training for surgeons and anesthesiologists using virtual reality. *Int J Comput Assist Radiol Surg, 15*(12), 2109-2118. | VR  Psychomotor skills | Offer training situations | Medicine  Pilot study with an anesthesiologist with 27 years of working experience and two laparoscopic surgeons. | Qualitative  Usability study (checking to see if the study/tool aligns with what you want to teach/learn) | He proposed VR prototype provides a new basis for interprofessional team training in surgery. It engages the training of problem-based communication during surgery and might open new directions for operating room training. |
| 142 | Compton, E. C., et al. (2020). Assessment of a virtual reality temporal bone surgical simulator: a national face and content validity study. *J Otolaryngol Head Neck Surg, 49*(1), 17. | VR  Psychomotor skills | Offer training situations | Medicine  62 participants: 32 were attending OHNS surgeons, and 30 were resident trainees. | Quantitative  Experimental - Comparison (control) | CardinalSim met acceptable criteria for face and content validity. This temporal bone virtual reality surgical simulation platform may enhance surgical training and be suitable for patient-specific surgical rehearsal for practicing Otolaryngologists. |
| 143 | De Ponti, R., et al. (2020). Pre-graduation medical training including virtual reality during COVID-19 pandemic: a report on students' perception. *BMC Med Educ, 20*(1), 332. | VR  Cognitive/Psychomotor skills | Offer training situations | Medicine  122 medical students | Quantitative  post only | During the COVID-19 pandemic, online medical training including simulated clinical scenarios avoided training interruption and the majority of participant students gave a positive response on the perceived quality of this training modality. During this time frame, a non-negligible proportion of students experienced difficulties in online access to this virtual reality platform. |

| 144 | Du, Y. C., et al. (2020). The impact of multi-person virtual reality competitive learning on anatomy education: a randomized controlled study. *BMC Med Educ, 20*(1), 343. | VR  Cognitive skills | Offer training situations | Medicine  25 university students. The participants were randomly assigned into one of three groups: the control group (CG), the single-player VR (SP) group, or the multi-player VR (MP) group. | Quantitative  Experimental - Comparison (control) | The results indicated that the proposed VR learning system had a positive impact on the anatomy learning. Although the between-player competition caused higher stress levels for the VR groups, the stress could have been a mediator of their learning outcomes. |
| --- | --- | --- | --- | --- | --- | --- |
| 145 | Fairen, M., et al. (2020). VR4Health: Personalized teaching and learning anatomy using VR. *J Med Syst, 44*(5), 94. | VR  Cognitive skills | Offer training situations | Medicine  6 teachers of human anatomy and 18 students | Quantitative  Usability study (checking to see if the study/tool aligns with what you want to teach/learn) | The results presented show that for students and teachers VR4Health is a self-learning tool that facilitates the understanding regarding the volume and the relationship among the different anatomical structures. |
| 146 | Gonzalez, A. et al. (2020). Augmented reality-based learning for the comprehension of cardiac physiology in undergraduate biomedical students. *Adv Physiol Educ, 44*(3), 314-322. | AR  Cognitive skills | Offer training situations | Health sciences  101 students from the Pontificia Universidad Católica de Valparaíso, Chile. 58 students were assigned to the experimental group, and 43 to the control group. | Quantitative  Pretest-Posttest | The results indicate that using AR enhances the comprehension of anatomical and physiological concepts of the cardiac cycle for undergraduate biomedical students. |
| 147 | Hanson, J., et al. (2020). The effects of a virtual learning environment compared with an individual handheld device on pharmacology knowledge acquisition, satisfaction and comfort ratings. *Nurse Educ Today, 92*, 104518. | VR  Cognitive skills | Offer training situations | Nursing  694 students | Quantitative  Pretest-Posttest | Three-dimensional artefacts using mobile devices is promising in terms of cost-effectiveness and accessibility for students with restricted access to on-campus teaching modes. |
| 148 | Hardie, P., et al. (2020). Nursing & Midwifery students' experience of immersive virtual reality storytelling: an evaluative study. *BMC Nurs, 19*, 78. | VR  Cognitive/Affective skills | Offer training situations | Nursing  132 students | Mixed  observational study | The full potential of this new medium of iVR storytelling has yet to be seen. However, this study provides an encouraging insight into the positive attributes of iVR storytelling that engages students and creates authentic active learning experiences. |
| 149 | Hecht, R., et al. (2020). Smartphone Augmented Reality CT-Based Platform for Needle Insertion Guidance: A Phantom Study. *Cardiovasc Intervent Radiol, 43*(5), 756-764. | AR  Psychomotor skills | Offer training situations | Medicine  11 operators (including interventional radiologists, non-interventional radiology physicians, and non-physicians) performed single-pass needle insertions using AR guidance (n = 8) and CT-guided freehand (n = 8). | Quantitative  Experimental - Comparison (control) | All operators exhibited superior needle insertion accuracy when using the smartphone-based AR guidance application compared to CT-guided freehand. This AR platform can potentially facilitate percutaneous biopsies and ablations by improving needle insertion accuracy, expediting procedural times, and reducing radiation exposures. |
| 150 | Henssen, D., et al. (2020). Neuroanatomy Learning: Augmented Reality vs. Cross-Sections. *Anat Sci Educ, 13*(3), 353-365. | AR  Cognitive skills | Offer training situations | Medicine  31 medical and biomedical | Mixed  Experimental - Comparison (control) | No significant differences were found in motivational scores. To conclude, this study suggests that AR applications can play a role in future anatomy education as an add-on educational tool, especially in learning three-dimensional relations of anatomical structures. |
| 151 | Hu, K. C., et al. (2020). Impact of virtual reality anatomy training on ultrasound competency development: A randomized controlled trial. *PloS one, 15*(11), e0242731. | VR  Psychomotor skills | Offer training situations | Medicine  101 medical students were randomized into intervention (n = 47) and control (n = 54) groups. | Quantitative  Randomized - Control Trial (RCT) | This study suggests that VR-enhanced anatomical training could be of significant benefit in ultrasonography training by promoting a better understanding of the spatial relationships of anatomical structures and the development of early psychomotor skills transferable to the handling of ultrasonographic probes. |
| 152 | Jaskiewicz, F., et al. (2020). Chest compressions quality during sudden cardiac arrest scenario performed in virtual reality: A crossover study in a training environment. *Medicine (Baltimore), 99*(48), e23374. | VR  Psychomotor skills | Offer training situations | Medicine  113 students | Quantitative  cross-sectional design | Virtual reality can be a safe and highly valued by medical students, method of hands-on CPR training. However additional VR equipment placed on the trainee’s body may cause chest compressions harder to provide. If it is not preceded by traditional training, the use of VR may have an adverse impact on depth and full chest relaxation during the training. |
| 153 | Katz, D., et al. (2020). Utilization of a Voice-Based Virtual Reality Advanced Cardiac Life Support Team Leader Refresher: Prospective Observational Study. *J Med Internet Res, 22*(3), e17425. | VR  Cognitive skills | Offer training situations | Health sciences  25 participants | Quantitative  Experimental - Comparison (control) | Utilization of a VR-based team leader refresher for ACLS skills is comparable with HFS in several areas, including learner satisfaction. The VR module was more cost-effective and was easier to proctor; however, HFS was better at delivering feedback to participants. Optimal education strategies likely contain elements of both modalities. |
| 154 | Kurul, R., et al. (2020). An Alternative Method for Anatomy Training: Immersive Virtual Reality. *Anat Sci Educ, 13*(5), 648-656. | VR  Psychomotor skills | Offer training situations | Medicine  72 students were included in the study. The students were randomized into control (n = 36) and VR (n = 36) group. | Quantitative  Pretest-Posttest | These results suggest that VR systems can be used as an alternative method to the conventional anatomy training approach for health students. |
| 155 | Lee, A. L., et al. (2020). The feasibility and acceptability of using virtual world technology for interprofessional education in palliative care: a mixed methods study. *J Interprof Care, 34*(4), 461-471. | VR  Cognitive skills | Offer training situations | Medicine  Student volunteers were recruited from a target audience of graduate students in medicine, nursing, nutrition, physical therapy, and social work. | Mixed  Pretest-Posttest | Students found the virtual world environment acceptable for interprofessional education focused on palliative care, based on qualitative results. As health professions schools develop interprofessional education curricula, the use of virtual world technology may be an important modality to consider, to effectively and conveniently bring interprofessional learners together. |
| 156 | Liu, L., et al. (2020). Simulation training for ceramic crown preparation in the dental setting using a virtual educational system. *Eur J Dent Educ, 24*(2), 199-206. | VR  Psychomotor skills | Offer training situations | Dentistry  57 students | Quantitative  Experimental - Comparison (control) | The application of a Virtual Educational System for Dentistry with the VLNP and RDTES in pre-clinical operative training helps students improve their clinical skills. |
| 157 | Lo, S., et al. (2020). Use of a virtual 3D anterolateral thigh model in medical education: Augmentation and not replacement of traditional teaching? *J Plast Reconstr Aesthet Surg, 73*(2), 269-275. | VR  Cognitive skills | Offer training situations | Medicine  52 final year undergraduate B.Sc. | Quantitative  Usability study (checking to see if the study/tool aligns with what you want to teach/learn) | This study highlights a striking user preference for virtual 3D models as compared to for traditional teaching methods. Nonetheless, 3D models are likely to enhance rather than replace lectures, with this study suggesting that teaching by experts is likely to remain an  essential part of medical education. |
| 158 | Lohre, R., et al. (2020). Improved Complex Skill Acquisition by Immersive Virtual Reality Training: A Randomized Controlled Trial. *J Bone Joint Surg Am, 102*(6), e26. | VR  Cognitive skills | Immersion training situation | Medicine  19 orthopaedic surgical residents (resident group) from postgraduate years (PGYs) 4 and 5, and 7 orthopaedic shoulder arthroplasty surgeons (expert group). | Quantitative  Randomized - Control Trial (RCT) | Immersive VR demonstrated substantially improved translational technical and nontechnical skills acquisition over traditional learning in senior orthopaedic residents. Additionally, the results demonstrate the face, content, construct, and transfer validity for immersive VR. |
| 159 | Lorenzo-Alvarez, R., et al. (2020). Game-Based Learning in Virtual Worlds: A Multiuser Online Game for Medical Undergraduate Radiology Education within Second Life. *Anat Sci Educ, 13*(5), 602-617. | VR  Cognitive skills | Offer training situations | Medicine  197 third-year medical students | Quantitative  Usability study (checking to see if the study/tool aligns with what you want to teach/learn) | Competitive game-based learning within Second Life is an effective and well-accepted means of teaching core radiological anatomy and radiological signs content to medical students. The higher medium-term outcomes obtained by participants may indicate effective learning with the game. Additionally, valuable positive perceptions about the game, the educational contents, and the potential benefit for their education were discovered among non-participants. |
| 160 | Mellum, M. L., et al.(2020). Virtual vitreoretinal surgery: effect of distracting factors on surgical performance in medical students. *Acta Ophthalmol, 98*(4), 378-383. | VR  Psychomotor skills | Offer training situations | Medicine  26 participants | Quantitative  Experimental - Comparison (control) | Virtual vitreoretinal surgery is an important tool for practicing complex surgical skills without compromising patient safety. In this study, deleterious effects on surgical performance were induced by four independent distracting factors. |
| 161 | Mills, B., et al. (2020). Virtual Reality Triage Training Can Provide Comparable Simulation Efficacy for Paramedicine Students Compared to Live Simulation-Based Scenarios. *Prehosp Emerg Care, 24*(4), 525-536. | VR  Cognitive skills | Offer training situations | Medicine  29 students enrolled in a Bachelor of Science (Paramedical Science) at ECU participated in the study. | Mixed  Usability study (checking to see if the study/tool aligns with what you want to teach/learn) | The VR simulation provided near identical simulation efficacy for paramedicine students compared to the live simulation. |
| 162 | Mirchi, N., et al. (2020). The Virtual Operative Assistant: An explainable artificial intelligence tool for simulation-based training in surgery and medicine. *PloS one, 15*(2), e0229596. | VR  Psychomotor skills | Offer training situations | Medicine  28 skilled participants (14 staff neurosurgeons, 4 fellows, 10 PGY 4–6 residents) and 22 novice participants (10 PGY 1–3 residents, 12 medical students) took part in this study. | Quantitative  Usability study (checking to see if the study/tool aligns with what you want to teach/learn) | The educational system outlined establishes a basis for the potential role of integrating artificial intelligence and virtual reality simulation into surgical educational teaching. The potential of linking expertise classification, objective feedback based on proficiency benchmarks, and instructor input creates a novel educational tool by integrating these three components into a formative educational paradigm. |
| 163 | Mladenovic, R., et al. (2020). Effect of augmented reality simulation on administration of local anaesthesia in paediatric patients. *Eur J Dent Educ, 24*(3), 507-512. | AR  Psychomotor skills | Offer training situations | Medicine  21 fourth- and fifth-year students enrolled in a 5-year dentistry programme. | Quantitative  Experimental - Comparison (control) | The AR concept may influence better manipulation and control of the syringe in students administering their first anaesthetic injection to paediatric patients, but may not reduce acute stress. |
| 164 | Mu, Y., et al. (2020). Augmented reality simulator for ultrasound-guided percutaneous renal access. *Int J Comput Assist Radiol Surg, 15*(5), 749-757. | AR  Psychomotor skills | Offer training situations | Medicine  6 expert (urologists and interventional radiologist) and 24 novice (undergraduate) participants were recruited to evaluate the efficacy of our simulator. | Quantitative  Usability study (checking to see if the study/tool aligns with what you want to teach/learn) | The cost-effective, flexible, and easily customizable AR training simulator can provide opportunities for trainees to acquire basic skills of US-guided PCA in a safe and stress-free environment. |
| 165 | Oussi, N., et al. (2020). Trainee Performance After Laparoscopic Simulator Training Using a Blackbox versus LapMentor. *J Surg Res, 250*, 1-11. | VR  Psychomotor skills | Offer training situations | Medicine  A cohort of 63 subjects volunteered to participate in the study. The subjects were fourth year medical students. | Quantitative  Randomized - Control Trial (RCT) | Given today’s knowledge of the benefits of laparoscopic simulator training, whether using a low- or high-tech simulator, this paper stress the importance of laparoscopic simulation training. Furthermore, the experience of the basic skills training procedures in the low-tech Blackbox seems to correlate with the students’ performance in the MIST-VR simulator with some gender-specific differences. |
| 166 | Pelanis, E., et al. (2020). Use of mixed reality for improved spatial understanding of liver anatomy. *Minim Invasive Ther Allied Technol, 29*(3), 154-160. | MR  Cognitive skills | Offer training situations | Medicine  The study included 28 participants. | Quantitative  Usability study (checking to see if the study/tool aligns with what you want to teach/learn) | The use of 3D liver models in mixed reality significantly decreases the time for tasks requiring a spatial understanding of the organ. This may significantly decrease operating time and improve use of resources. |
| 167 | Peterson, E., et al. (2020). Mixed-Reality Simulation for a Pediatric Transport Team: A Pilot Study. *Air Med J, 39*(3), 173-177. | MR  Affective outcomes | Offer training situations | nurses therapists technicians  10 nurses, 9 respiratory therapists, and 8 emergency medical technicians participated. | Quantitative  Pretest-Posttest | Mixed-reality simulation programs can enhance standard technical skills training by providing an additional relational element. Such programs are translatable to other institutions. |
| 168 | Richards, J. P., et al. (2020). Virtual coach: the next tool in functional endoscopic sinus surgery education. *Int Forum Allergy Rhinol, 10*(1), 97-102. | VR  Cognitive/Psychomotor skills | Offer training situations | Medicine  17 surgeons were grouped into novice (n =10) and expert (n =7). | Quantitative  Experimental - Comparison (control) | The virtual coach provides a useful tool to enhance FESS education by providing objective real-time data in a novel mixed-reality surgical environment. |
| 169 | Ruthberg, J. S., et al. (2020). Mixed reality as a time-efficient alternative to cadaveric dissection. *Med Teach, 42*(8), 896-901. | MR  Psychomotor skills | Offer training situations | Medicine  185 medical students | Quantitative  Experimental - Comparison (control) | The results indicate that HoloAnatomy may decrease the time necessary for anatomy didactics without sacrificing student understanding of the material. |
| 170 | Sapkaroski, D., et al. (2020). Virtual reality versus conventional clinical role-play for radiographic positioning training: A students' perception study. *Radiography (Lond), 26*(1), 57-62. | VR  Psychomotor skills | Offer training situations | Medicine  Participants in this study were Year 1 students enrolled in the Bachelor of Radiography and Medical Imaging (Hons). | Mixed  Experimental - Comparison (control) | The perceptions of novice students in training for radiographic hand positioning tasks, using either a VR SLE or clinical role-play scenario, did not differ. There was a strong similarity in common themes, however, a key point of difference identified was the benefit of repetition afforded by the VR simulation, in contrast to the need for more time using traditional role-play in a constrained laboratory setting. |
| 171 | Schoeb, D. S., et al. (2020). Mixed reality for teaching catheter placement to medical students: a randomized single-blinded, prospective trial. *BMC Med Educ, 20*(1), 510. | MR  Psychomotor skills | Offer training situations | Medicine  Study enrolled 164 medical students. Students were randomized into 2 groups. One group (107 students) were given their instructions by an instructor, while the other group (57 students) were instructed via an MR guidance system using a Microsoft HoloLens. | Quantitative  a randomized single-blinded, prospective trial | MR is a promising tool for instructing practical skills, and has the potential to enable superior learning outcomes. Advances in MR technology are necessary to improve the usability of current system. |
| 172 | Shao, X., et al. (2020). Virtual reality technology for teaching neurosurgery of skull base tumor. *BMC Med Educ, 20*(1), 3. | VR  Cognitive skills | Offer training situations | Medicine  60 clinical undergraduates were randomly divided into two groups: 1) the traditional teaching group (n=30), and 2) the virtual reality-teaching group (n=30). | Quantitative  Randomized - Control Trial (RCT) | This study showed that VR technology might improve neurosurgical skull base teaching quality, which should be promoted in the teaching of clinical subjects. |
| 173 | Shorey, S., et al. (2020). Communication skills training using virtual reality: A descriptive qualitative study. *Nurse Educ Today, 94*, 104592. | VR  Affective outcomes | Offer training situations | Nursing  24 nursing undergraduates and six clinical facilitators | Qualitative  post test only | The mixed attitudes toward virtual patient interactions and recognitions of the benefits of virtual patient simulations suggest the potential effectiveness of the use of virtual patients in teaching effective nursing communication skills. |
| 174 | Singh, A., et al. (2020). Using Virtual Reality in Biomedical Engineering Education. *J Biomech Eng, 142*(11). | VR  Cognitive skills | Offer training situations | Nursing  Simulation labs in a 300 level biomedical device engineering course (n =22 BME) that was cross-listed with a 300-level evidence-based practices nursing course (n =12). In-person communication simulation labs were conducted with the BME (n =12) and nursing students (n =12) who volunteered to participate in the optional lab sessions of the courses that was being recorded for this study. | Quantitative  Usability study (checking to see if the study/tool aligns with what you want to teach/learn) | The study concluded that VR holds promise as an educational tool to offer simulated clinical scenarios that are effective in training BME students for interprofessional collaborations. |
| 175 | Slamon, N., et al. (2020). 1057: Virtual Reality Simulation for Pediatric Airway Intubation Training. *Critical Care Medicine, 48*(1), 508-508. | VR  Cognitive skills | Offer training situations | Medicine  Two study groups were randomized (n=17). Pediatric residents and first-year fellows (n=7) were included in the VR group. | Quantitative  Experimental - Comparison (control) | VR technologies can be used for education in preparation for pediatric airway intubation. There was no difference in the performance accuracy between the two groups. Larger studies are essential to study benefits of VR in preparation and performance of airway intubation. |
| 176 | Tran, C., et al. (2020). A virtual patient model for students' interprofessional learning in primary healthcare. *PloS one, 15*(9), e0238797. | VR  Cognitive skills | Offer training situations | Nursing, physiotherapy, medicine, and occupational therapy  39 students | Qualitative  test theory model | The students perceived that the mixture of text and multimedia made the virtual patient seem authentic and stimulated their group discussions, which they valued most. The students gave generally high points for usability in the questionnaire, but they also gave input for improvement of the program in their comments. |
| 177 | Vaughan, N., et al. (2020). Scoring and assessment in medical VR training simulators with dynamic time series classification. *Engineering Applications of Artificial Intelligence, 94*. | VR  Psychomotor skills | Offer training situations | Medicine  7 participants were in two groups: Group-C contained 3 medical trained NHS clinicians. Group-N contained 4 non-clinicians who were not medically trained. | Quantitative  Usability study (checking to see if the study/tool aligns with what you want to teach/learn) | Expert VR data recordings can be used for guidance of novices. Assessment feedback can help trainees to improve skills and consistency. Motion analysis can identify different techniques used by individuals. Mistakes can be detected dynamically in real-time, raising alarms to prevent injuries. |
| 178 | Vincent, M., et al. (2020). Contribution of Haptic Simulation to Analogic Training Environment in Restorative Dentistry. *J Dent Educ, 84*(3), 367-376. | VR  Psychomotor skills | Offer training situations | Dentistry  88 first-year dental students were randomly assigned to one of two groups: group 1 (n=45) was assigned to cavity preparations on a haptic simulator (Virteasy) and group 2 (n=43) was assigned to conventional practical work on plastic analogue teeth (Kavo). | Quantitative  Experimental - Comparison (control) | In this study, virtual reality allowed an assessment based on objective criteria and reduced the subjectivity of evaluations conducted on plastic analogue teeth. Considering the saving of supervision and teaching time as well as the material gain offered by virtual reality, the learning methods of haptic simulators are educational options that should be considered by dental educators. |
| 179 | Williams, D., et al. (2020). Teaching interprofessional competencies using virtual simulation: A descriptive exploratory research study. *Nurse Educ Today, 93*, 104535. | VR  Cognitive skills | Offer training situations | Nursing  57 students | Mixed  Pretest-Posttest | Virtual reality provides students with the opportunity to collaborate for safe patient care. Students were able to strengthen their knowledge of interprofessional competencies. |
| 180 | Wilson, G., et al. (2020). Virtual Reality and Physical Models in Undergraduate Orthopaedic Education: A Modified Randomised Crossover Trial. *Orthop Res Rev, 12*, 97-104. | VR  Psychomotor skills | VR is an effective resource for supplementing surgical education within orthopaedic surgery. | Medicine  49 undergraduate medical students were randomly allocated to two groups. | Quantitative  modified randomised crossover trial | Both VR and physical models represent valuable educational adjuncts for the undergraduate medical curriculum. Both have demonstrated improvements in immediate and long-term knowledge retention of key orthopaedic concepts. |
| 181 | Wu, S. H., et al. (2020). Effect of virtual reality training to decreases rates of needle stick/sharp injuries in new-coming medical and nursing interns in Taiwan. *J Educ Eval Health Prof, 17*, 1. | VR  Cognitive/Affective skills |  | Nursing  The study invited 59 new Chinese nursing and 50 new medical interns to join this new intervention at the beginning of their internship, typically within the first week. | Qualitative  Pretest-Posttest | This self-developed VR game system using Gagne’s flow improved universal precaution for needlestick or sharp injury prevention and reduced the needlestick or sharp injury rates in the first 2 months of nursing and medical internship. |
| 182 | Xin, B., et al. (2020). The efficacy of immersive virtual reality surgical simulator training for pedicle screw placement: a randomized double-blind controlled trial. *Int Orthop, 44*(5), 927-934. | VR  Psychomotor skills | Offer training situation | Medicine  Study participants comprised 16 surgical graduate students. | Quantitative  Randomized - Control Trial (RCT) | The immersive VR surgical simulator for PSP training model is superior to the traditional training model in terms of accuracy, success rate, and efficiency, showing potential in training new orthopedic spine surgeons. |
| 183 | Zafar, S., et al. (2020). Evaluation of HoloHuman augmented reality application as a novel educational tool in dentistry. *Eur J Dent Educ, 24*(2), 259-265. | AR  Psychomotor skills | Offer training situations | Dentistry  90 students were invited to participate in the study. | Quantitative  Pretest-Posttest | This study suggested that the use of AR offers an additional mean of dental anatomy training; however, it cannot be used as a replacement for traditional modes of cadaver anatomy training. AR has the potential to be used as an adjunct tool in the learning of dental head and neck anatomy as it has demonstrated increased student engagement and enjoyment. |
| 184 | Zhang, B., et al. (2020). Virtual versus jaw simulation in Oral implant education: a randomized controlled trial. *BMC Med Educ, 20*(1), 272. | VR  Psychomotor skills | Offer training situations | Dentistry  166 students were divided into two groups | Quantitative  Randomized - Control Trial (RCT) | Virtual simulation education, especially with a jaw simulation model, could improve students’ implantology achievements and training. Currently study found that the V-J group may performed better than the J-V group in oral implant teaching. |

**References**

**2015**

Abelson, J. S., Silverman, E., Banfelder, J., Naides, A., Costa, R., & Dakin, G. (2015). Virtual operating room for team training in surgery. *The American Journal of Surgery*, *210*(3), 585-590.

Azarnoush, H., Alzhrani, G., Winkler-Schwartz, A., Alotaibi, F., Gelinas-Phaneuf, N., Pazos, V., ... & Del Maestro, R. F. (2015). Neurosurgical virtual reality simulation metrics to assess psychomotor skills during brain tumor resection. *International journal of computer assisted radiology and surgery*, *10*(5), 603-618.

Chowriappa, A., Raza, S. J., Fazili, A., Field, E., Malito, C., Samarasekera, D., ... & Eun, D. D. (2015). Augmented‐reality‐based skills training for robot‐assisted urethrovesical anastomosis: a multi‐institutional randomised controlled trial. *BJU international*, *115*(2), 336-345.

Dharmawardana, N., Ruthenbeck, G., Woods, C., Elmiyeh, B., Diment, L., Ooi, E. H., ... & Carney, A. S. (2015). Validation of virtual‐reality‐based simulations for endoscopic sinus surgery. *Clinical Otolaryngology*, *40*(6), 569-579.

Ferracani, A., Pezzatini, D., Seidenari, L., & Del Bimbo, A. (2015). Natural and virtual environments for the training of emergency medicine personnel. *Universal Access in the Information Society*, *14*(3), 351-362.

Ferrer-Torregrosa, J., Torralba, J., Jimenez, M. A., García, S., & Barcia, J. M. (2015). ARBOOK: Development and assessment of a tool based on augmented reality for anatomy. *Journal of Science Education and Technology*, *24*(1), 119-124.

Freschi, C., Parrini, S., Dinelli, N., Ferrari, M., & Ferrari, V. (2015). Hybrid simulation using mixed reality for interventional ultrasound imaging training. *International journal of computer assisted radiology and surgery*, *10*(7), 1109-1115.

Fucentese, S. F., Rahm, S., Wieser, K., Spillmann, J., Harders, M., & Koch, P. P. (2015). Evaluation of a virtual-reality-based simulator using passive haptic feedback for knee arthroscopy. *Knee Surgery, Sports Traumatology, Arthroscopy*, *23*(4), 1077-1085.

Gomez, P. P., Willis, R. E., & Van Sickle, K. R. (2015). Development of a virtual reality robotic surgical curriculum using the da Vinci Si surgical system. *Surgical endoscopy*, *29*(8), 2171-2179.

Grover, S. C., Garg, A., Scaffidi, M. A., Jeffrey, J. Y., Plener, I. S., Yong, E., ... & Walsh, C. M. (2015). Impact of a simulation training curriculum on technical and nontechnical skills in colonoscopy: a randomized trial. *Gastrointestinal endoscopy*, *82*(6), 1072-1079.

Hashimoto, D. A., Sirimanna, P., Gomez, E. D., Beyer-Berjot, L., Ericsson, K. A., Williams, N. N., ... & Aggarwal, R. (2015). Deliberate practice enhances quality of laparoscopic surgical performance in a randomized controlled trial: from arrested development to expert performance. *Surgical endoscopy*, *29*(11), 3154-3162

Holloway, T., Lorsch, Z. S., Chary, M. A., Sobotka, S., Moore, M. M., Costa, A. B., ... & Bederson, J. (2015). Operator experience determines performance in a simulated computer-based brain tumor resection task. *International journal of computer assisted radiology and surgery*, *10*(11), 1853-1862.

Huang, C., Cheng, H., Bureau, Y., Agrawal, S. K., & Ladak, H. M. (2015). Face and content validity of a virtual-reality simulator for myringotomy with tube placement. *Journal of Otolaryngology-Head & Neck Surgery*, *44*(1), 40.

Hudson, K., Taylor, L. A., Kozachik, S. L., Shaefer, S. J., & Wilson, M. L. (2015). Second Life simulation as a strategy to enhance decision‐making in diabetes care: a case study. *Journal of clinical nursing*, *24*(5-6), 797-804.

Jacobsen, M. E., Andersen, M. J., Hansen, C. O., & Konge, L. (2015). Testing basic competency in knee arthroscopy using a virtual reality simulator: exploring validity and reliability. *JBJS*, *97*(9), 775-781.

Levac, D., Espy, D., Fox, E., Pradhan, S., & Deutsch, J. E. (2015). “Kinect-ing” with clinicians: A knowledge translation resource to support decision making about video game use in rehabilitation. *Physical therapy*, *95*(3), 426-440.

Suzuki, T., Egi, H., Hattori, M., Tokunaga, M., Sawada, H., & Ohdan, H. (2015). An evaluation of the endoscopic surgical skills assessment using a video analysis software program. *Surgical endoscopy*, *29*(7), 1804-1808.

**2016**

Allen, L. K., Eagleson, R., & de Ribaupierre, S. (2016). Evaluation of an online three‐dimensional interactive resource for undergraduate neuroanatomy education. *Anatomical sciences education*, *9*(5), 431-439.

Diment, L. E., Ruthenbeck, G. S., Dharmawardana, N., Carney, A. S., Woods, C. M., Ooi, E. H., & Reynolds, K. J. (2016). Comparing surgical experience with performance on a sinus surgery simulator. *ANZ journal of surgery*, *86*(12), 990-995.

Dorozhkin, D., Nemani, A., Roberts, K., Ahn, W., Halic, T., Dargar, S., ... & De, S. (2016). Face and content validation of a Virtual Translumenal Endoscopic Surgery Trainer (VTEST™). *Surgical endoscopy*, *30*(12), 5529-5536.

Ferrer-Torregrosa, J., Jiménez-Rodríguez, M. Á., Torralba-Estelles, J., Garzón-Farinós, F., Pérez-Bermejo, M., & Fernández-Ehrling, N. (2016). Distance learning ects and flipped classroom in the anatomy learning: comparative study of the use of augmented reality, video and notes. *BMC medical education*, *16*(1), 230.

Fischer, M., Fuerst, B., Lee, S. C., Fotouhi, J., Habert, S., Weidert, S., ... & Navab, N. (2016). Preclinical usability study of multiple augmented reality concepts for K-wire placement. *International journal of computer assisted radiology and surgery*, *11*(6), 1007-1014.

Hu, A., Shewokis, P. A., Ting, K., & Fung, K. (2016). Motivation in computer‐assisted instruction. *The Laryngoscope*, *126*, S5-S13.

Küçük, S., Kapakin, S., & Göktaş, Y. (2016). Learning anatomy via mobile augmented reality: effects on achievement and cognitive load. *Anatomical sciences education*, *9*(5), 411-421.

Lin, D., Pena, G., Field, J., Altree, M., Marlow, N., Babidge, W., ... & Maddern, G. (2016). What are the demographic predictors in laparoscopic simulator performance?. *ANZ journal of surgery*, *86*(12), 983-989.

Ma, M., Fallavollita, P., Seelbach, I., Von Der Heide, A. M., Euler, E., Waschke, J., & Navab, N. (2016). Personalized augmented reality for anatomy education. *Clinical Anatomy*, *29*(4), 446-453.

Mathiowetz, V., Yu, C. H., & Quake‐Rapp, C. (2016). Comparison of a gross anatomy laboratory to online anatomy software for teaching anatomy. *Anatomical sciences education*, *9*(1), 52-59.

Medellín-Castillo, H. I., Govea-Valladares, E. H., Pérez-Guerrero, C. N., Gil-Valladares, J., Lim, T., & Ritchie, J. M. (2016). The evaluation of a novel haptic-enabled virtual reality approach for computer-aided cephalometry. *Computer methods and programs in biomedicine*, *130*, 46-53.

Middleton, R. M., Baldwin, M. J., Akhtar, K., Alvand, A., & Rees, J. L. (2016). Which global rating scale?: A comparison of the ASSET, BAKSSS, and IGARS for the assessment of simulated arthroscopic skills. *JBJS*, *98*(1), 75-81.

Miki, T., Iwai, T., Kotani, K., Dang, J., Sawada, H., & Miyake, M. (2016). Development of a virtual reality training system for endoscope-assisted submandibular gland removal. *Journal of Cranio-Maxillofacial Surgery*, *44*(11), 1800-1805.

Miller, M. (2016). Use of computer‐aided holographic models improves performance in a cadaver dissection‐based course in gross anatomy. *Clinical Anatomy*, *29*(7), 917-924.

Mueller, C. L., Kaneva, P., Fried, G. M., Mellinger, J. D., Marks, J. M., Dunkin, B. J., ... & Vassiliou, M. C. (2016). Validity evidence for a new portable, lower-cost platform for the fundamentals of endoscopic surgery skills test. *Surgical endoscopy*, *30*(3), 1107-1112.

Nickel, F., Hendrie, J. D., Bruckner, T., Kowalewski, K. F., Kenngott, H. G., Müller-Stich, B. P., & Fischer, L. (2016). Successful learning of surgical liver anatomy in a computer-based teaching module. *International journal of computer assisted radiology and surgery*, *11*(12), 2295-2301.

Pan, X., Slater, M., Beacco, A., Navarro, X., Rivas, A. I. B., Swapp, D., ... & Delacroix, S. (2016). The responses of medical general practitioners to unreasonable patient demand for antibiotics-a study of medical ethics using immersive virtual reality. *PloS one*, *11*(2), e0146837.

Peterson, D. C., & Mlynarczyk, G. S. (2016). Analysis of traditional versus three‐dimensional augmented curriculum on anatomical learning outcome measures. *Anatomical sciences education*, *9*(6), 529-536.

Rahm, S., Germann, M., Hingsammer, A., Wieser, K., & Gerber, C. (2016). Validation of a virtual reality-based simulator for shoulder arthroscopy. *Knee Surgery, Sports Traumatology, Arthroscopy*, *24*(5), 1730-1737.

Rasmussen, S. R., Konge, L., Mikkelsen, P. T., Sørensen, M. S., & Andersen, S. A. (2016). Notes from the field: Secondary task precision for cognitive load estimation during virtual reality surgical simulation training. *Evaluation & the health professions*, *39*(1), 114-120.

Sankaranarayanan, G., Li, B., Miller, A., Wakily, H., Jones, S. B., Schwaitzberg, S., ... & Olasky, J. (2016). Face validation of the virtual electrosurgery skill trainer (VEST©). *Surgical endoscopy*, *30*(2), 730-738.

Teeter, W., Romagnoli, A., Hoehn, M., Menaker, J., Stein, D., Scalea, T., & Brenner, M. (2016). 323 Virtual Reality Simulation Can Help Prepare Emergency Physicians for REBOA. *Annals of Emergency Medicine*, *68*(4), S124-S125.

Valdis, M., Chu, M. W., Schlachta, C., & Kiaii, B. (2016). Evaluation of robotic cardiac surgery simulation training: a randomized controlled trial. *The Journal of thoracic and cardiovascular surgery*, *151*(6), 1498-1505.

Van Nuland, S. E., & Rogers, K. A. (2016). The anatomy of E‐Learning tools: Does software usability influence learning outcomes?. *Anatomical sciences education*, *9*(4), 378-390.

**2017**

Agbetoba, A., Luong, A., Siow, J. K., Senior, B., Callejas, C., Szczygielski, K., & Citardi, M. J. (2017, February). Educational utility of advanced three‐dimensional virtual imaging in evaluating the anatomical configuration of the frontal recess. In *International forum of allergy & rhinology 7*( 2), 143-148.

Alsalamah, A., Campo, R., Tanos, V., Grimbizis, G., Van Belle, Y., Hood, K., ... & Amso, N. (2017). Face and content validity of the virtual reality simulator ‘ScanTrainer®’. *Gynecological surgery*, *14*(1), 18.

Al‐Saud, L. M., Mushtaq, F., Allsop, M. J., Culmer, P. C., Mirghani, I., Yates, E., ... & Manogue, M. (2017). Feedback and motor skill acquisition using a haptic dental simulator. *European Journal of Dental Education*, *21*(4), 240-247.

Al‐Saud, L. M., Mushtaq, F., Mirghani, I. A., Balkhoyor, A., Keeling, A., Manogue, M., & Mon‐Williams, M. A. (2017). Drilling into the functional significance of stereopsis: the impact of stereoscopic information on surgical performance. *Ophthalmic and Physiological Optics*, *37*(4), 498-506.

Bourdel, N., Collins, T., Pizarro, D., Bartoli, A., Da Ines, D., Perreira, B., & Canis, M. (2017). Augmented reality in gynecologic surgery: evaluation of potential benefits for myomectomy in an experimental uterine model. *Surgical endoscopy*, *31*(1), 456-461.

Cui, D., Wilson, T. D., Rockhold, R. W., Lehman, M. N., & Lynch, J. C. (2017). Evaluation of the effectiveness of 3D vascular stereoscopic models in anatomy instruction for first year medical students. *Anatomical sciences education*, *10*(1), 34-45.

De La Garza, J. R., Kowalewski, K. F., Friedrich, M., Schmidt, M. W., Bruckner, T., Kenngott, H. G., ... & Nickel, F. (2017). Does rating the operation videos with a checklist score improve the effect of E-learning for bariatric surgical training? Study protocol for a randomized controlled trial. *Trials*, *18*(1), 134.

Dubovi, I., Levy, S. T., & Dagan, E. (2017). Now I know how! The learning process of medication administration among nursing students with non-immersive desktop virtual reality simulation. *Computers & Education*, *113*, 16-27.

Dubovsky, S. L., Antonius, D., Ellis, D. G., Ceusters, W., Sugarman, R. C., Roberts, R., ... & Butler, L. D. (2017). A preliminary study of a novel emergency department nursing triage simulation for research applications. *BMC research notes*, *10*(1), 15.

Huber, T., Paschold, M., Hansen, C., Wunderling, T., Lang, H., & Kneist, W. (2017). New dimensions in surgical training: immersive virtual reality laparoscopic simulation exhilarates surgical staff. *Surgical endoscopy*, *31*(11), 4472-4477.

Locketz, G. D., Lui, J. T., Chan, S., Salisbury, K., Dort, J. C., Youngblood, P., & Blevins, N. H. (2017). Anatomy-specific virtual reality simulation in temporal bone dissection: perceived utility and impact on surgeon confidence. *Otolaryngology–Head and Neck Surgery*, *156*(6), 1142-1149.

Mathews, S., Brodman, M., D'Angelo, D., Chudnoff, S., McGovern, P., Kolev, T., ... & Kischak, P. (2017). Predictors of laparoscopic simulation performance among practicing obstetrician gynecologists. *American journal of obstetrics and gynecology*, *217*(5), 596-e1.

Moro, C., Štromberga, Z., Raikos, A., & Stirling, A. (2017). The effectiveness of virtual and augmented reality in health sciences and medical anatomy. *Anatomical sciences education*, *10*(6), 549-559.

Sampogna, G., Pugliese, R., Elli, M., Vanzulli, A., & Forgione, A. (2017). Routine clinical application of virtual reality in abdominal surgery. *Minimally Invasive Therapy & Allied Technologies*, *26*(3), 135-143.

Saratzis, A., Calderbank, T., Sidloff, D., Bown, M. J., & Davies, R. S. (2017). Role of simulation in endovascular aneurysm repair (EVAR) training: a preliminary study. *European Journal of Vascular and Endovascular Surgery*, *53*(2), 193-198.

Siebert, J. N., Ehrler, F., Gervaix, A., Haddad, K., Lacroix, L., Schrurs, P., ... & Manzano, S. (2017). Adherence to AHA guidelines when adapted for augmented reality glasses for assisted pediatric cardiopulmonary resuscitation: A randomized controlled trial. *Journal of medical Internet research*, *19*(5), e183.

Sirimanna, P., & Gladman, M. A. (2017). Development of a proficiency‐based virtual reality simulation training curriculum for laparoscopic appendicectomy. *ANZ journal of surgery*, *87*(10), 760-766.

Siroen, K. L., Ward, C. D., Escoto, A., Naish, M. D., Bureau, Y., Patel, R. V., ... & Trejos, A. L. (2017). Mastery Learning–does the method of learning make a difference in skills acquisition for robotic surgery?. *The International Journal of Medical Robotics and Computer Assisted Surgery*, *13*(4), e1828.

Stepan, K., Zeiger, J., Hanchuk, S., Del Signore, A., Shrivastava, R., Govindaraj, S., & Iloreta, A. (2017, October). Immersive virtual reality as a teaching tool for neuroanatomy. In *International forum of allergy & rhinology* (Vol. 7, No. 10, pp. 1006-1013).

Våpenstad, C., Hofstad, E. F., Bø, L. E., Kuhry, E., Johnsen, G., Mårvik, R., ... & Hernes, T. N. (2017). Lack of transfer of skills after virtual reality simulator training with haptic feedback. *Minimally Invasive Therapy & Allied Technologies*, *26*(6), 346-354.

Wang, S., Parsons, M., Stone-McLean, J., Rogers, P., Boyd, S., Hoover, K., ... & Smith, A. (2017). Augmented reality as a telemedicine platform for remote procedural training. *Sensors*, *17*(10), 2294.

Yen, A. J., & Ramanathan, S. (2017). Advanced cataract learning experience in United States ophthalmology residency programs. *Journal of Cataract & Refractive Surgery*, *43*(10), 1350-1355.

Zupanc, C. M., Wallis, G. M., Hill, A., Burgess-Limerick, R., Riek, S., Plooy, A. M., ... & Hewett, D. G. (2017). Assessing colonoscopic inspection skill using a virtual withdrawal simulation: a preliminary validation of performance metrics. *BMC medical education*, *17*(1), 118.

**2018**

Ali, S., Qandeel, M., Ramakrishna, R., & Yang, C. W. (2018). Virtual simulation in enhancing procedural training for fluoroscopy-guided lumbar puncture: a pilot study. *Academic radiology*, *25*(2), 235-239.

Arya, S., Mulla, Z. D., & Kupesic Plavsic, S. (2018). Role of pelvic ultrasound simulation. *The clinical teacher*, *15*(6), 457-461.

Borgersen, N. J., Skou Thomsen, A. S., Konge, L., Sørensen, T. L., & Subhi, Y. (2018). Virtual reality‐based proficiency test in direct ophthalmoscopy. *Acta ophthalmologica*, *96*(2), e259-e261.

Cecil, J., Gupta, A., & Pirela-Cruz, M. (2018). An advanced simulator for orthopedic surgical training. *International journal of computer assisted radiology and surgery*, *13*(2), 305-319.

Chalhoub, M., Khazzaka, A., Sarkis, R., & Sleiman, Z. (2018). The role of smartphone game applications in improving laparoscopic skills. *Advances in medical education and practice*, *9*, 541.

Cook, D. A., Aljamal, Y., Pankratz, V. S., Sedlack, R. E., Farley, D. R., & Brydges, R. (2019). Supporting self-regulation in simulation-based education: a randomized experiment of practice schedules and goals. *Advances in Health Sciences Education*, *24*(2), 199-213.

Courteille, O., Fahlstedt, M., Ho, J., Hedman, L., Fors, U., Von Holst, H., ... & Möller, H. (2018). Learning through a virtual patient vs. recorded lecture: a comparison of knowledge retention in a trauma case. *International journal of medical education*, *9*, 86.

Gunn, T., Jones, L., Bridge, P., Rowntree, P., & Nissen, L. (2018). The use of virtual reality simulation to improve technical skill in the undergraduate medical imaging student. *Interactive Learning Environments*, *26*(5), 613-620.

Hettig, J., Engelhardt, S., Hansen, C., & Mistelbauer, G. (2018). AR in VR: Assessing surgical augmented reality visualizations in a steerable virtual reality environment. *International journal of computer assisted radiology and surgery*, *13*(11), 1717-1725.

Hovgaard, L. H., Andersen, S. A. W., Konge, L., Dalsgaard, T., & Larsen, C. R. (2018). Validity evidence for procedural competency in virtual reality robotic simulation, establishing a credible pass/fail standard for the vaginal cuff closure procedure. *Surgical endoscopy*, *32*(10), 4200-4208.

Huang, C. Y., Thomas, J. B., Alismail, A., Cohen, A., Almutairi, W., Daher, N. S., ... & Tan, L. D. (2018). The use of augmented reality glasses in central line simulation:“see one, simulate many, do one competently, and teach everyone”. *Advances in medical education and practice*, *9*, 357.

Huang, Z., Song, W., Zhang, Y., Zhang, Q., Zhou, D., Zhou, X., & He, Y. (2018). Three-dimensional printing model improves morphological understanding in acetabular fracture learning: A multicenter, randomized, controlled study. *PloS one*, *13*(1), e0191328.

Javaux, A., Bouget, D., Gruijthuijsen, C., Stoyanov, D., Vercauteren, T., Ourselin, S., ... & Vander Poorten, E. (2018). A mixed-reality surgical trainer with comprehensive sensing for fetal laser minimally invasive surgery. *International journal of computer assisted radiology and surgery*, *13*(12), 1949-1957.

Kay, R., Goulding, H., & Li, J. (2018). Assessing the impact of a virtual lab in an allied health program. *Journal of allied health*, *47*(1), 45-50.

Korzeniowski, P., White, R. J., & Bello, F. (2018). VCSim3: a VR simulator for cardiovascular interventions. *International journal of computer assisted radiology and surgery*, *13*(1), 135-149.

Kugelmann, D., Stratmann, L., Nühlen, N., Bork, F., Hoffmann, S., Samarbarksh, G., ... & Navab, N. (2018). An augmented reality magic mirror as additive teaching device for gross anatomy. *Annals of Anatomy-Anatomischer Anzeiger*, *215*, 71-77.

Lamb, R., Antonenko, P., Etopio, E., & Seccia, A. (2018). Comparison of virtual reality and hands on activities in science education via functional near infrared spectroscopy. *Computers & Education*, *124*, 14-26.

Linsk, A. M., Monden, K. R., Sankaranarayanan, G., Ahn, W., Jones, D. B., De, S., ... & Cao, C. G. (2018). Validation of the VBLaST pattern cutting task: a learning curve study. *Surgical endoscopy*, *32*(4), 1990-2002.

Liu, W., Zheng, X., Wu, R., Jin, Y., Kong, S., Li, J., ... & Zhang, X. (2018). Novel laparoscopic training system with continuously perfused ex-vivo porcine liver for hepatobiliary surgery. *Surgical endoscopy*, *32*(2), 743-750.

Llena, C., Folguera, S., Forner, L., & Rodríguez‐Lozano, F. J. (2018). Implementation of augmented reality in operative dentistry learning. *European Journal of Dental Education*, *22*(1), 122-130.

Maresky, H. S., Oikonomou, A., Ali, I., Ditkofsky, N., Pakkal, M., & Ballyk, B. (2019). Virtual reality and cardiac anatomy: Exploring immersive three‐dimensional cardiac imaging, a pilot study in undergraduate medical anatomy education. *Clinical Anatomy*, *32*(2), 238-243.

Mirghani, I., Mushtaq, F., Allsop, M. J., Al‐Saud, L. M., Tickhill, N., Potter, C., ... & Manogue, M. (2018). Capturing differences in dental training using a virtual reality simulator. *European Journal of Dental Education*, *22*(1), 67-71.

Nayar, S. K., Musto, L., Fernandes, R., & Bharathan, R. (2018). Validation of a virtual reality laparoscopic appendicectomy simulator: a novel process using cognitive task analysis. *Irish Journal of Medical Science (1971-)*, 1-9.

Nemani, A., Ahn, W., Cooper, C., Schwaitzberg, S., & De, S. (2018). Convergent validation and transfer of learning studies of a virtual reality-based pattern cutting simulator. *Surgical endoscopy*, *32*(3), 1265-1272.

Ng, D. S. C., Sun, Z., Young, A. L., Ko, S. T. C., Lok, J. K. H., Lai, T. Y. Y., ... & Tham, C. C. (2018). impact of virtual reality simulation on learning barriers of phacoemulsification perceived by residents. *Clinical ophthalmology (Auckland, NZ)*, *12*, 885.

Nomura, T., Matsutani, T., Hagiwara, N., Fujita, I., Nakamura, Y., Kanazawa, Y., ... & Uchida, E. (2018). Characteristics predicting laparoscopic skill in medical students: nine years’ experience in a single center. *Surgical endoscopy*, *32*(1), 96-104.

Perin, A., Galbiati, T. F., Gambatesa, E., Ayadi, R., Orena, E. F., Cuomo, V., . . . Group, E. N. S. S. (2018). Filling the gap between the OR and virtual simulation: a European study on a basic neurosurgical procedure. *Acta Neurochirurgica, 160*(11), 2087-2097.

Pulijala, Y., Ma, M., Pears, M., Peebles, D., & Ayoub, A. (2018). Effectiveness of immersive virtual reality in surgical training—A randomized control trial. *Journal of Oral and Maxillofacial Surgery*, *76*(5), 1065-1072.

Pulijala, Y., Ma, M., Pears, M., Peebles, D., & Ayoub, A. (2018). An innovative virtual reality training tool for orthognathic surgery. *International journal of oral and maxillofacial surgery*, *47*(9), 1199-1205.

Rai, A., Scovell, J. M., Xu, A., Balasubramanian, A., Siller, R., Kohn, T., ... & Link, R. E. (2018). Patient-specific Virtual Simulation—A State of the Art Approach to Teach Renal Tumor Localization. *Urology*, *120*, 42-48.

Raison, N., Ahmed, K., Abe, T., Brunckhorst, O., Novara, G., Buffi, N., ... & Dasgupta, P. (2018). Cognitive training for technical and non‐technical skills in robotic surgery: a randomised controlled trial. *BJU international*, *122*(6), 1075-1081.

Rojas-Muñoz, E., Cabrera, M. E., Andersen, D., Popescu, V., Marley, S., Mullis, B., ... & Wachs, J. (2018). Surgical Telementoring Without Encumbrance: A Comparative Study of See-through Augmented Reality-based Approaches. *Annals of surgery*.

Sgouros, N. P., Loukas, C., Koufi, V., Troupis, T. G., & Georgiou, E. (2018). An automated skills assessment framework for laparoscopic training tasks. *The International Journal of Medical Robotics and Computer Assisted Surgery*, *14*(1), e1853.

Sugiura, A., Kitama, T., Toyoura, M., & Mao, X. (2018). The Use of Augmented Reality Technology in Medical Specimen Museum Tours. *Anatomical sciences education*.

Won, T. B., Hwang, P., Lim, J. H., Cho, S. W., Paek, S. H., Losorelli, S., ... & Blevins, N. H. (2018, January). Early experience with a patient‐specific virtual surgical simulation for rehearsal of endoscopic skull‐base surgery. In *International forum of allergy & rhinology* (Vol. 8, No. 1, pp. 54-63).

Yiasemidou, M., Galli, R., Glassman, D., Tang, M., Aziz, R., Jayne, D., & Miskovic, D. (2018). Patient-specific mental rehearsal with interactive visual aids: a path worth exploring?. *Surgical endoscopy*, *32*(3), 1165-1173.

Yoganathan, S., Finch, D. A., Parkin, E., & Pollard, J. (2018). 360 virtual reality video for the acquisition of knot tying skills: a randomised controlled trial. *International Journal of Surgery*, *54*, 24-27.

**2019**

Alismail, A., Thomas, J., Daher, N. S., Cohen, A., Almutairi, W., Terry, M. H., . . . Tan, L. D. (2019). Augmented reality glasses improve adherence to evidence-based intubation practice. *Adv Med Educ Pract*, *10*, 279-286.

Bayram, S. B., & Caliskan, N. (2019). Effect of a game-based virtual reality phone application on tracheostomy care education for nursing students: A randomized controlled trial. *Nurse Educ Today*, *79*, 25-31.

Cold, K. M., Konge, L., Clementsen, P. F., & Nayahangan, L. J. (2019). Simulation-Based Mastery Learning of Flexible Bronchoscopy: Deciding Factors for Completion. *Respiration*, *97*(2), 160-167.

Cook, D. A., Aljamal, Y., Pankratz, V. S., Sedlack, R. E., Farley, D. R., & Brydges, R. (2019). Supporting self-regulation in simulation-based education: a randomized experiment of practice schedules and goals. *Adv Health Sci Educ Theory Pract*, *24*(2), 199-213.

Dardick, J., Allen, S., Scoco, A., Zampolin, R. L., & Altschul, D. J. (2019). Virtual reality simulation of neuroendovascular intervention improves procedure speed in a cohort of trainees. *Surg Neurol Int*, *10*, 184.

Erolin, C., Reid, L., & McDougall, S. (2019). Using virtual reality to complement and enhance anatomy education. *J Vis Commun Med*, *42*(3), 93-101.

Frendo, M., Thingaard, E., Konge, L., Sorensen, M. S., & Andersen, S. A. W. (2019). Decentralized virtual reality mastoidectomy simulation training: a prospective, mixed-methods study. *Eur Arch Otorhinolaryngol*, *276*(10), 2783-2789.

Gustafsson, A., Pedersen, P., Romer, T. B., Viberg, B., Palm, H., & Konge, L. (2019). Hip-fracture osteosynthesis training: exploring learning curves and setting proficiency standards. *Acta Orthop*, *90*(4), 348-353.

Hanson, J., Andersen, P., & Dunn, P. K. (2019). Effectiveness of three-dimensional visualisation on undergraduate nursing and midwifery students' knowledge and achievement in pharmacology: A mixed methods study. *Nurse Educ Today*, *81*, 19-25.

Jensen, K., Hansen, H. J., Petersen, R. H., Neckelmann, K., Vad, H., Moller, L. B., . . . Konge, L. (2019). Evaluating competency in video-assisted thoracoscopic surgery (VATS) lobectomy performance using a novel assessment tool and virtual reality simulation. *Surg Endosc*, *33*(5), 1465-1473.

Kim-Berman, H., Karl, E., Sherbel, J., Sytek, L., & Ramaswamy, V. (2019). Validity and User Experience in an Augmented Reality Virtual Tooth Identification Test. *J Dent Educ*, *83*(11), 1345-1352.

Kowalewski, K. F., Minassian, A., Hendrie, J. D., Benner, L., Preukschas, A. A., Kenngott, H. G., . . . Nickel, F. (2019). One or two trainees per workplace for laparoscopic surgery training courses: results from a randomized controlled trial. *Surg Endosc*, *33*(5), 1523-1531.

Liaw, S. Y., Soh, S. L., Tan, K. K., Wu, L. T., Yap, J., Chow, Y. L., . . . Wong, L. F. (2019). Design and evaluation of a 3D virtual environment for collaborative learning in interprofessional team care delivery. *Nurse Educ Today*, *81*, 64-71.

Lindquist, N. R., Leach, M., Simpson, M. C., & Antisdel, J. L. (2019). Evaluating Simulator-Based Teaching Methods for Endoscopic Sinus Surgery. *Ear Nose Throat J*, *98*(8), 490-495.

Lorenzo-Alvarez, R., Rudolphi-Solero, T., Ruiz-Gomez, M. J., & Sendra-Portero, F. (2019). Medical Student Education for Abdominal Radiographs in a 3D Virtual Classroom Versus Traditional Classroom: A Randomized Controlled Trial. *AJR Am J Roentgenol*, *213*(3), 644-650.

Makransky, G., Mayer, R. E., Veitch, N., Hood, M., Christensen, K. B., & Gadegaard, H. (2019). Equivalence of using a desktop virtual reality science simulation at home and in class. *PloS one*, *14*(4), e0214944.

Maresky, H. S., Oikonomou, A., Ali, I., Ditkofsky, N., Pakkal, M., & Ballyk, B. (2019). Virtual reality and cardiac anatomy: Exploring immersive three-dimensional cardiac imaging, a pilot study in undergraduate medical anatomy education. *Clin Anat*, *32*(2), 238-243.

Ochs, M., Mestre, D., de Montcheuil, G., Pergandi, J.-M., Saubesty, J., Lombardo, E., . . . Blache, P. (2019). Training doctors’ social skills to break bad news: evaluation of the impact of virtual environment displays on the sense of presence. *Journal on Multimodal User Interfaces*, *13*(1), 41-51.

Sattar, M. U., Palaniappan, S., Lokman, A., Hassan, A., Shah, N., & Riaz, Z. (2019). Effects of Virtual Reality training on medical students' learning motivation and competency. *Pak J Med Sci*, 35(3), 852-857.

Savran, M. M., Nielsen, A. B., Poulsen, B. B., Thorsen, P. B., & Konge, L. (2019). Using virtual-reality simulation to ensure basic competence in hysteroscopy. *Surg Endosc*, *33*(7), 2162-2168.

Schlosser, P. D., Grundgeiger, T., Sanderson, P. M., & Happel, O. (2019). An exploratory clinical evaluation of a head-worn display based multiple-patient monitoring application: impact on supervising anesthesiologists' situation awareness. *J Clin Monit Comput*, *33*(6), 1119-1127.

Schmidt, M. W., Kowalewski, K. F., Schmidt, M. L., Wennberg, E., Garrow, C. R., Paik, S., . . . Nickel, F. (2019). The Heidelberg VR Score: development and validation of a composite score for laparoscopic virtual reality training. *Surg Endosc*, *33*(7), 2093-2103.

Shirk, J. D., Kwan, L., & Saigal, C. (2019). The Use of 3-Dimensional, Virtual Reality Models for Surgical Planning of Robotic Partial Nephrectomy. *Urology*, 125, 92-97.

Sultan, L., Abuznadah, W., Al-Jifree, H., Khan, M. A., Alsaywid, B., & Ashour, F. (2019). An Experimental Study On Usefulness Of Virtual Reality 360 degrees In Undergraduate Medical Education. *Adv Med Educ Pract*, *10*, 907-916.

Takagi, D., Hayashi, M., Iida, T., Tanaka, Y., Sugiyama, S., Nishizaki, H., & Morimoto, Y. (2019). Effects of dental students’ training using immersive virtual reality technology for home dental practice. Educational *Gerontology*, *45*(11), 670-680.

Van der Heijden, L. L. M., Reijman, M., van der Steen, M. C. M., Janssen, R. P. A., & Tuijthof, G. J. M. (2019). Validation of Simendo Knee Arthroscopy Virtual Reality Simulator. *Arthroscopy*, 35(8), 2385-2390.

Vera, J., Diaz-Piedra, C., Jimenez, R., Sanchez-Carrion, J. M., & Di Stasi, L. L. (2019). Intraocular pressure increases after complex simulated surgical procedures in residents: an experimental study. *Surg Endosc*, *33*(1), 216-224.

Wilson, E., Janssens, S., McLindon, L. A., Hewett, D. G., Jolly, B., & Beckmann, M. (2019). Improved laparoscopic skills in gynaecology trainees following a simulation-training program using take-home box trainers. *Aust N Z J Obstet Gynaecol*, 59(1), 110-116.

Wong, D. T., Mehta, A., Singh, K. P., Leong, S. M., Ooi, A., Niazi, A., . . . Wong, J. (2019). The effect of virtual reality bronchoscopy simulator training on performance of bronchoscopic-guided intubation in patients: A randomised controlled trial. *Eur J Anaesthesiol*, 36(3), 227-233.

Zackoff, M. W., Real, F. J., Cruse, B., Davis, D., & Klein, M. (2019). Medical Student Perspectives on the Use of Immersive Virtual Reality for Clinical Assessment Training. *Acad Pediatr*, 19(7), 849-851.

Zare Bidaki, M., & Ehteshampour, A. (2019). Designing, Producing, Application, and Evaluation of Virtual Reality-Based Multimedia Clips for Learning Purposes of Medical and Nursing Students. *Chest*, 155(4), 166A.

Zhou, Z., Hu, S., Zhao, Y. Z., Zhu, Y. J., Wang, C. F., Gu, X., . . . He, S. S. (2019). Feasibility of Virtual Reality Combined with Isocentric Navigation in Transforaminal Percutaneous Endoscopic Discectomy: A Cadaver Study. *Orthop Surg*, *11*(3), 493-499.

Zhou, Z. Y., Jiang, S., Yang, Z. Y., & Zhou, L. (2019). Personalized planning and training system for brachytherapy based on virtual reality. *Virtual Reality*, *23*(4), 347-361.

**2020**

Aussedat, C., Robier, M., Aoustin, J. M., Parietti-Winkler, C., Lescanne, E., Bonnard, D., . . . Bakhos, D. (2020). Using virtual reality in audiological training: Our experience in 22 otolaryngology residents. *Clin Otolaryngol, 45*(4), 643-648.

Azzam, N., Khamis, N., Almadi, M., Batwa, F., Alsohaibani, F., Aljebreen, A., . . . Satava, R. M. (2020). Development and validation of metric-based-training to proficiency simulation curriculum for upper gastrointestinal endoscopy using a novel assessment checklist. *Saudi J Gastroenterol*.

Baran, B., Kaptanoglu, S. N., Esen, E., & Siyez, D. M. (2020). Reproductive System Augmented Reality Application for Sexual Health Classes. *International Journal of Sexual Health, 32*(4), 408-420.

Bartlett, J. D., Lawrence, J. E., Yan, M., Guevel, B., Stewart, M. E., Audenaert, E., & Khanduja, V. (2020). The learning curves of a validated virtual reality hip arthroscopy simulator. *Arch Orthop Trauma Surg, 140*(6), 761-767.

Berg, H., & Steinsbekk, A. (2020). Is individual practice in an immersive and interactive virtual reality application non-inferior to practicing with traditional equipment in learning systematic clinical observation? A randomized controlled trial. *BMC Med Educ, 20*(1), 123.

Bogomolova, K., van der Ham, I. J. M., Dankbaar, M. E. W., van den Broek, W. W., Hovius, S. E. R., van der Hage, J. A., & Hierck, B. P. (2020). The Effect of Stereoscopic Augmented Reality Visualization on Learning Anatomy and the Modifying Effect of Visual-Spatial Abilities: A Double-Center Randomized Controlled Trial. *Anat Sci Educ, 13*(5), 558-567.

Chen, S., Zhu, J., Cheng, C., Pan, Z., Liu, L., Du, J., . . . Pan, H. (2020). Can virtual reality improve traditional anatomy education programmes? A mixed-methods study on the use of a 3D skull model. *BMC Med Educ, 20*(1), 395.

Chheang, V., Fischer, V., Buggenhagen, H., Huber, T., Huettl, F., Kneist, W., . . . Hansen, C. (2020). Toward interprofessional team training for surgeons and anesthesiologists using virtual reality. *Int J Comput Assist Radiol Surg, 15*(12), 2109-2118.

Compton, E. C., Agrawal, S. K., Ladak, H. M., Chan, S., Hoy, M., Nakoneshny, S. C., . . . Lui, J. T. (2020). Assessment of a virtual reality temporal bone surgical simulator: a national face and content validity study. *J Otolaryngol Head Neck Surg, 49*(1), 17.

De Ponti, R., Marazzato, J., Maresca, A. M., Rovera, F., Carcano, G., & Ferrario, M. M. (2020). Pre-graduation medical training including virtual reality during COVID-19 pandemic: a report on students' perception. *BMC Med Educ, 20*(1), 332.

Du, Y. C., Fan, S. C., & Yang, L. C. (2020). The impact of multi-person virtual reality competitive learning on anatomy education: a randomized controlled study. *BMC Med Educ, 20*(1), 343.

Fairen, M., Moyes, J., & Insa, E. (2020). VR4Health: Personalized teaching and learning anatomy using VR. *J Med Syst, 44*(5), 94.

Gonzalez, A. A., Lizana, P. A., Pino, S., Miller, B. G., & Merino, C. (2020). Augmented reality-based learning for the comprehension of cardiac physiology in undergraduate biomedical students. *Adv Physiol Educ, 44*(3), 314-322.

Hanson, J., Andersen, P., & Dunn, P. K. (2020). The effects of a virtual learning environment compared with an individual handheld device on pharmacology knowledge acquisition, satisfaction and comfort ratings. *Nurse Educ Today, 92*, 104518.

Hardie, P., Darley, A., Carroll, L., Redmond, C., Campbell, A., & Jarvis, S. (2020). Nursing & Midwifery students' experience of immersive virtual reality storytelling: an evaluative study. *BMC Nurs, 19*, 78.

Hecht, R., Li, M., de Ruiter, Q. M. B., Pritchard, W. F., Li, X., Krishnasamy, V., . . . Wood, B. J. (2020). Smartphone Augmented Reality CT-Based Platform for Needle Insertion Guidance: A Phantom Study. *Cardiovasc Intervent Radiol, 43*(5), 756-764.

Henssen, D., van den Heuvel, L., De Jong, G., Vorstenbosch, M., van Cappellen van Walsum, A. M., Van den Hurk, M. M., . . . Bartels, R. (2020). Neuroanatomy Learning: Augmented Reality vs. Cross-Sections. *Anat Sci Educ, 13*(3), 353-365.

Hu, K. C., Salcedo, D., Kang, Y. N., Lin, C. W., Hsu, C. W., Cheng, C. Y., . . . Huang, W. C. (2020). Impact of virtual reality anatomy training on ultrasound competency development: A randomized controlled trial. *PloS one, 15*(11), e0242731.

Jaskiewicz, F., Kowalewski, D., Starosta, K., Cierniak, M., & Timler, D. (2020). Chest compressions quality during sudden cardiac arrest scenario performed in virtual reality: A crossover study in a training environment. *Medicine (Baltimore), 99*(48), e23374.

Katz, D., Shah, R., Kim, E., Park, C., Shah, A., Levine, A., & Burnett, G. (2020). Utilization of a Voice-Based Virtual Reality Advanced Cardiac Life Support Team Leader Refresher: Prospective Observational Study. *J Med Internet Res, 22*(3), e17425.

Kurul, R., Ogun, M. N., Neriman Narin, A., Avci, S., & Yazgan, B. (2020). An Alternative Method for Anatomy Training: Immersive Virtual Reality. *Anat Sci Educ, 13*(5), 648-656.

Lee, A. L., DeBest, M., Koeniger-Donohue, R., Strowman, S. R., & Mitchell, S. E. (2020). The feasibility and acceptability of using virtual world technology for interprofessional education in palliative care: a mixed methods study. *J Interprof Care, 34*(4), 461-471.

Liu, L., Zhou, R., Yuan, S., Sun, Z., Lu, X., Li, J., . . . Wang, L. (2020). Simulation training for ceramic crown preparation in the dental setting using a virtual educational system. *Eur J Dent Educ, 24*(2), 199-206.

Lo, S., Abaker, A. S. S., Quondamatteo, F., Clancy, J., Rea, P., Marriott, M., & Chapman, P. (2020). Use of a virtual 3D anterolateral thigh model in medical education: Augmentation and not replacement of traditional teaching? *J Plast Reconstr Aesthet Surg, 73*(2), 269-275.

Lohre, R., Bois, A. J., Athwal, G. S., Goel, D. P., Canadian, S., & Elbow, S. (2020). Improved Complex Skill Acquisition by Immersive Virtual Reality Training: A Randomized Controlled Trial. *J Bone Joint Surg Am, 102*(6), e26.

Lorenzo-Alvarez, R., Rudolphi-Solero, T., Ruiz-Gomez, M. J., & Sendra-Portero, F. (2020). Game-Based Learning in Virtual Worlds: A Multiuser Online Game for Medical Undergraduate Radiology Education within Second Life. *Anat Sci Educ, 13*(5), 602-617.

Mellum, M. L., Vestergaard, A. H., Grauslund, J., & Vergmann, A. S. (2020). Virtual vitreoretinal surgery: effect of distracting factors on surgical performance in medical students. *Acta Ophthalmol, 98*(4), 378-383.

Mills, B., Dykstra, P., Hansen, S., Miles, A., Rankin, T., Hopper, L., . . . Bartlett, D. (2020). Virtual Reality Triage Training Can Provide Comparable Simulation Efficacy for Paramedicine Students Compared to Live Simulation-Based Scenarios. *Prehosp Emerg Care, 24*(4), 525-536.

Mirchi, N., Bissonnette, V., Yilmaz, R., Ledwos, N., Winkler-Schwartz, A., & Del Maestro, R. F. (2020). The Virtual Operative Assistant: An explainable artificial intelligence tool for simulation-based training in surgery and medicine. *PloS one, 15*(2), e0229596.

Mladenovic, R., Dakovic, D., Pereira, L., Matvijenko, V., & Mladenovic, K. (2020). Effect of augmented reality simulation on administration of local anaesthesia in paediatric patients. *Eur J Dent Educ, 24*(3), 507-512.

Mu, Y., Hocking, D., Wang, Z. T., Garvin, G. J., Eagleson, R., & Peters, T. M. (2020). Augmented reality simulator for ultrasound-guided percutaneous renal access. *Int J Comput Assist Radiol Surg, 15*(5), 749-757.

Oussi, N., Enochsson, L., Henningsohn, L., Castegren, M., Georgiou, E., & Kjellin, A. (2020). Trainee Performance After Laparoscopic Simulator Training Using a Blackbox versus LapMentor. *J Surg Res, 250*, 1-11.

Pelanis, E., Kumar, R. P., Aghayan, D. L., Palomar, R., Fretland, A. A., Brun, H., . . . Edwin, B. (2020). Use of mixed reality for improved spatial understanding of liver anatomy. *Minim Invasive Ther Allied Technol, 29*(3), 154-160.

Peterson, E., Porter, M., & Calhoun, A. (2020). Mixed-Reality Simulation for a Pediatric Transport Team: A Pilot Study. *Air Med J, 39*(3), 173-177.

Richards, J. P., Done, A. J., Barber, S. R., Jain, S., Son, Y. J., & Chang, E. H. (2020). Virtual coach: the next tool in functional endoscopic sinus surgery education. *Int Forum Allergy Rhinol, 10*(1), 97-102.

Ruthberg, J. S., Tingle, G., Tan, L., Ulrey, L., Simonson-Shick, S., Enterline, R., . . . Wish-Baratz, S. (2020). Mixed reality as a time-efficient alternative to cadaveric dissection. *Med Teach, 42*(8), 896-901.

Sapkaroski, D., Mundy, M., & Dimmock, M. R. (2020). Virtual reality versus conventional clinical role-play for radiographic positioning training: A students' perception study. *Radiography (Lond), 26*(1), 57-62.

Schoeb, D. S., Schwarz, J., Hein, S., Schlager, D., Pohlmann, P. F., Frankenschmidt, A., . . . Miernik, A. (2020). Mixed reality for teaching catheter placement to medical students: a randomized single-blinded, prospective trial. *BMC Med Educ, 20*(1), 510.

Shao, X., Yuan, Q., Qian, D., Ye, Z., Chen, G., le Zhuang, K., . . . Qiang, D. (2020). Virtual reality technology for teaching neurosurgery of skull base tumor. *BMC Med Educ, 20*(1), 3.

Shorey, S., Ang, E., Ng, E. D., Yap, J., Lau, L. S. T., & Chui, C. K. (2020). Communication skills training using virtual reality: A descriptive qualitative study. *Nurse Educ Today, 94*, 104592.

Singh, A., Ferry, D., Ramakrishnan, A., & Balasubramanian, S. (2020). Using Virtual Reality in Biomedical Engineering Education. *J Biomech Eng, 142*(11).

Slamon, N., & Agasthya, N. (2020). 1057: Virtual Reality Simulation for Pediatric Airway Intubation Training. *Critical Care Medicine, 48*(1), 508-508.

Tran, C., Toth-Pal, E., Ekblad, S., Fors, U., & Salminen, H. (2020). A virtual patient model for students' interprofessional learning in primary healthcare. *PloS one, 15*(9), e0238797.

Vaughan, N., & Gabrys, B. (2020). Scoring and assessment in medical VR training simulators with dynamic time series classification. *Engineering Applications of Artificial Intelligence, 94*.

Vincent, M., Joseph, D., Amory, C., Paoli, N., Ambrosini, P., Mortier, E., & Tran, N. (2020). Contribution of Haptic Simulation to Analogic Training Environment in Restorative Dentistry. *J Dent Educ, 84*(3), 367-376.

Williams, D., Stephen, L. A., & Causton, P. (2020). Teaching interprofessional competencies using virtual simulation: A descriptive exploratory research study. *Nurse Educ Today, 93*, 104535.

Wilson, G., Zargaran, A., Kokotkin, I., Bhaskar, J., Zargaran, D., & Trompeter, A. (2020). Virtual Reality and Physical Models in Undergraduate Orthopaedic Education: A Modified Randomised Crossover Trial. *Orthop Res Rev, 12*, 97-104.

Wu, S. H., Huang, C. C., Huang, S. S., Yang, Y. Y., Liu, C. W., Shulruf, B., & Chen, C. H. (2020). Effect of virtual reality training to decreases rates of needle stick/sharp injuries in new-coming medical and nursing interns in Taiwan. *J Educ Eval Health Prof, 17*, 1.

Xin, B., Huang, X., Wan, W., Lv, K., Hu, Y., Wang, J., . . . Liu, T. (2020). The efficacy of immersive virtual reality surgical simulator training for pedicle screw placement: a randomized double-blind controlled trial. *Int Orthop, 44*(5), 927-934.

Zafar, S., & Zachar, J. J. (2020). Evaluation of HoloHuman augmented reality application as a novel educational tool in dentistry. *Eur J Dent Educ, 24*(2), 259-265.

Zhang, B., Li, S., Gao, S., Hou, M., Chen, H., He, L., . . . Zhang, K. (2020). Virtual versus jaw simulation in Oral implant education: a randomized controlled trial. *BMC Med Educ, 20*(1), 272.
